# Estimating the predictive power of silent mutations on cancer classification and prognosis

**DOI:** 10.1038/s41525-021-00229-1

**Published:** 2021-08-12

**Authors:** Tal Gutman, Guy Goren, Omri Efroni, Tamir Tuller

**Affiliations:** 1 Department of Biomedical Engineering, the Engineering Faculty, Tel Aviv University, Tel-Aviv, Israel; 2 Department of Electrical Engineering, the Engineering Faculty, Tel Aviv University, Tel-Aviv, Israel

**Keywords:** Genome informatics, Predictive markers, Systems biology, Gene expression

## Abstract

In recent years it has been shown that silent mutations, in and out of the coding region, can affect gene expression and may be related to tumorigenesis and cancer cell fitness. However, the predictive ability of these mutations for cancer type diagnosis and prognosis has not been evaluated yet. In the current study, based on the analysis of 9,915 cancer genomes and approximately three million mutations, we provide a comprehensive quantitative evaluation of the predictive power of various types of silent and non-silent mutations over cancer classification and prognosis. The results indicate that silent-mutation models outperform the equivalent null models in classifying all examined cancer types and in estimating the probability of survival 10 years after the initial diagnosis. Additionally, combining both non-silent and silent mutations achieved the best classification results for 68% of the cancer types and the best survival estimation results for up to nine years after the diagnosis. Thus, silent mutations hold considerable predictive power over both cancer classification and prognosis, most likely due to their effect on gene expression. It is highly advised that silent mutations are integrated in cancer research in order to unravel the full genomic landscape of cancer and its ramifications on cancer fitness.

## Introduction

The rapid developments of New Generation Sequencing (NGS) technologies and acceleration of computational abilities over the past few years have led to the availability of extensive genomic information^1–5^. Multiple research utilizing these high-dimensional data establish cancer as a group of highly heterogeneous genomic diseases, characterized by large inter-tumor and intra-tumor diversities^6–8^. Moreover, common genetic features were repeatedly identified among patients of different cancer types and significant diversities were found among patients diagnosed with the same cancer type^9,10^. These findings highlight the need for personalized, gene-targeted cancer treatments.

By now, hundreds of genes had been recognized as cancer drivers^11^ and many more are currently researched. Some, like *TP53*^12^, *BRAF*^13^, *EGFR*^14^, or *IDH1*^15^ have already been targeted for gene therapy. Nonetheless, there are still numerous obstacles to overcome in order to fully unravel the cancer genomic landscape. Currently, most contemporary research is based on data derived by Whole Exome Sequencing (WES)^2^. In addition, most studies focus exclusively or predominantly on non-silent mutations; alterations in the coding regions that cause a change in the amino-acid sequence of the produced protein. Silent mutations, such as modifications in the introns, the untranslated regions (UTR’5 and UTR’3), or even synonymous mutations in the coding region itself are by and large excluded from the analyses^16^.

Yet, cancerous silent mutations could have detrimental effects on gene expression^16–19^, which in some cases could even lead to consequences more significant than non-silent mutations. Mutations in regulatory regions, such as promoters or enhancers, can destruct or form new transcription-factor binding sites and cause changes in transcription regulation^20–23^. Mutations in the untranslated regions can affect translation regulation or modify microRNA binding sites and thus impact mRNA stability^24^. Synonymous mutations can alter all aspects of gene expression^25^, impacting translation rates^26,27^, protein-folding^28^, transcription^29–31^, mRNA stability^32^, and splicing^33,34^. Overall, silent mutations could modify all phases of the gene expression process, causing amplification or reduction in protein quantities. Hence, even though most silent mutations do not cause a change in protein functionality, they could dramatically change protein abundance and could therefore influence cancer fitness.

We believe that including these mutations in cancer research is imperative for acquiring a broader understanding of the genomic landscape profoundly linked with cancer development and progression. Specifically, we believe that silent mutations should be incorporated when building predictive models.

The incredible heterogeneity of cancerous genomes, even for patients who presumably possess the same cancer type, highly complicates predictive tasks. When examining only non-silent mutations we miss a large part of the complex mutational patterns of these cancerous genomes; considering the full patterns could improve predictions. Additionally, silent driver mutations, even though considered today as infrequent compared to non-silent drivers, could be highly influential^35^ and thus also beneficial for predictive models. Indeed, there are previous studies that have demonstrated that silent mutations or non-silent mutations that modulate gene expression can significantly affect the phenotype of the cancer cell and its survival^33,36–42^. Additionally, some contemporary studies identified silent mutations that are recurrent for specific cancer types and are possible drivers of cancer^20,23,35^. However, to the best of our knowledge, no previous study has performed a broad, quantitative comparison between the predictive abilities of various mutation types on cancer classification and progression. In this study, we explore silent and non-silent mutations, aiming to quantify the predictive ability of various types of silent mutations to perform cancer diagnosis and to estimate patients’ survival probabilities over time, while comparing it to the performance of non-silent mutations.

## Results

### Data processing and feature engineering

**Fig. 1:**
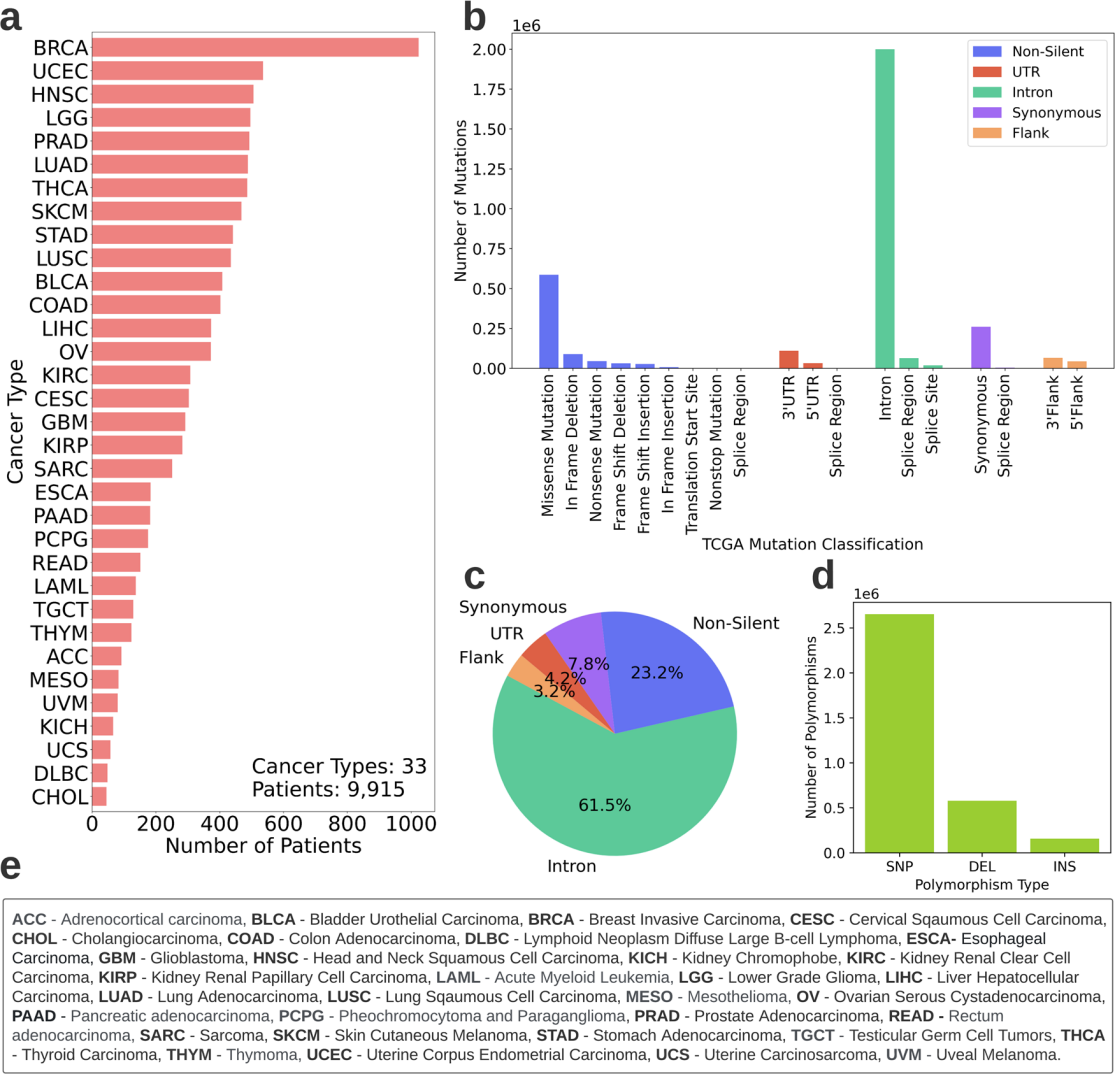
TCGA data characteristics. Description of the data retrieved from TCGA after initial preprocessing (discarding patients with missing genomic or clinical data and patients with multiple genomic samples). Overall, 9915 patients across 33 cancer types are included in the study. **a** Patient distribution across cancer types. **b** Sorting TCGA mutations to five categories for the study. The *x*-axis depicts the mutation classification according to TCGA*. The *y*-axis depicts the number of mutations in the TCGA mutation categories. The legend depicts the five categories to which the mutations are sorted for this study. *Note: In TCGA, Synonymous mutations are referred to as “Silent”. As the terms are in fact not interchangeable (synonymous mutations are a subcategory of silent mutations) we replace the term “Silent” with “Synonymous” where needed. **c** Mutation type distribution. The distribution includes all mutations of the 9915 patients. **d** Polymorphism type distribution. Mutations could be either Single Nucleotide Polymorphisms (SNP), Deletions (DEL) or Insertions (INS). The distribution includes all mutations of the 9915 patients, **e** Names and abbreviations of the 33 cancer types.

**Fig. 2:**
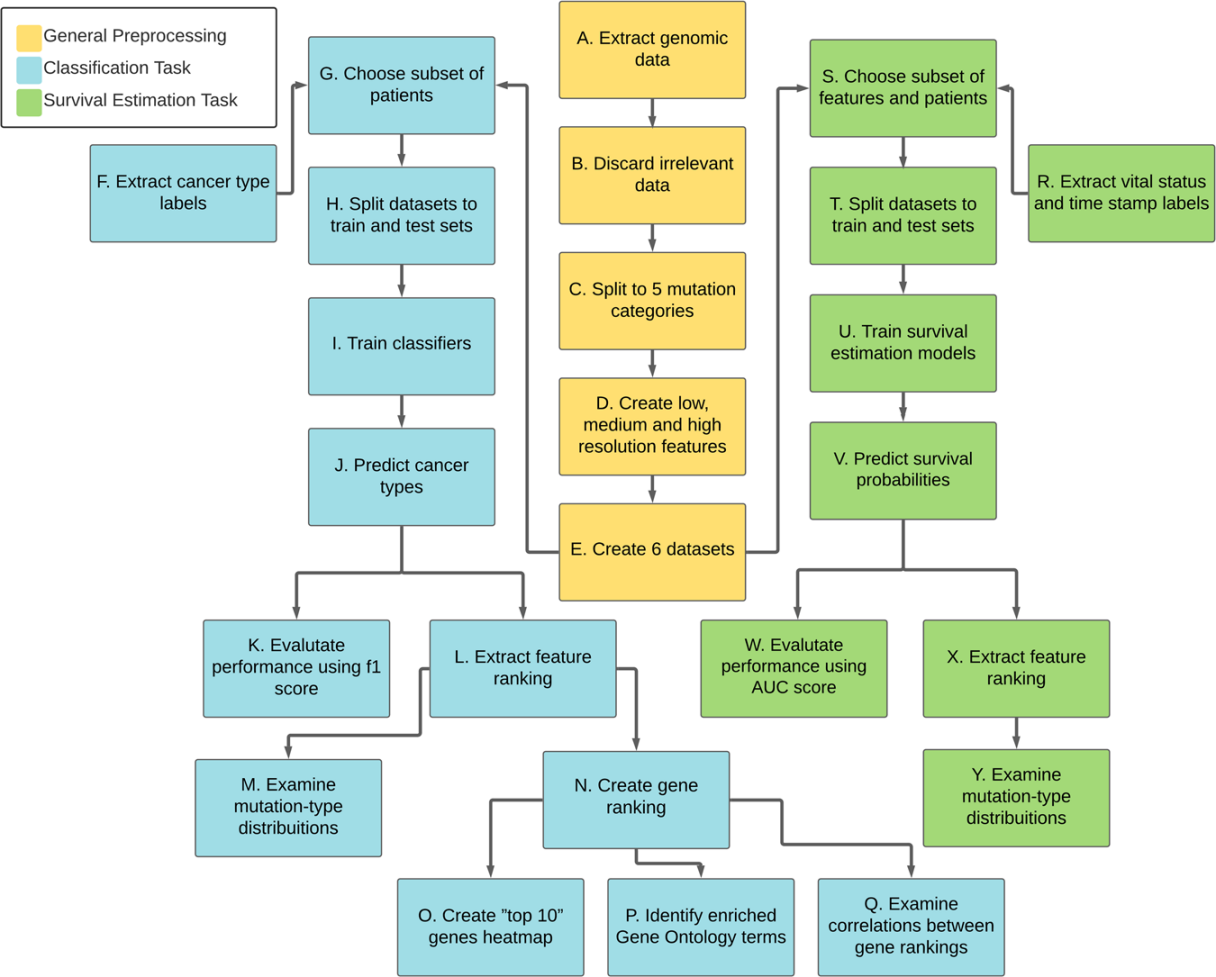
The flow chart of the study. Yellow boxes denote preprocessing steps performed for both tasks. Blue boxes denote steps performed for the cancer type classification task and green boxes denote steps performed for the survival probability estimation task.

Genomic and clinical data of 9915 patients across 33 cancer types were obtained from The Cancer Genome Atlas (TCGA)^43^. Data characteristics are described in Fig. 1. The genomic data consisted of detailed information about the patients’ DNA mutations while the clinical data held personal information such as patients’ vital status. These data were used to perform two tasks- patients’ cancer type classification and survival estimation. The full flow chart of the study is depicted in Fig. 2.

As Fig. 2 indicates, the genomic data was split into five categories. One category holds all non-silent mutations (amino-acid-altering exonic mutations). The other four categories consist of silent mutations from different regions within and adjacent to the genes; synonymous mutations (exonic mutations that do not directly affect the amino acids), mutations in introns, UTRs or flanking regions. It is important to note that a genomic position is considered mutated for a patient only if its nucleic acid content differs between the patient’s cancerous and healthy tissue samples.

**Fig. 3:**
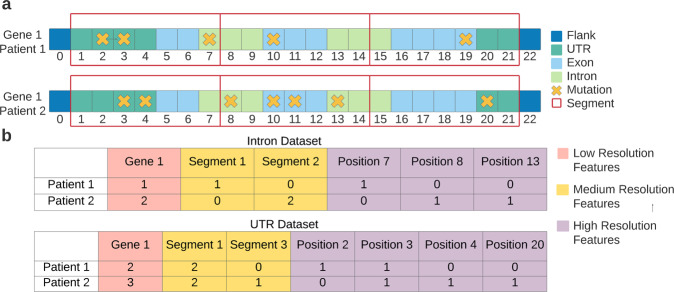
A simplified illustration of the feature extraction process. **a** A representation of the initial genomic information. The X’s denote mutations that two patients have in the same gene. The red rectangular frames represent the 50-nucleotide-long segments used for the medium-resolution features. **b** An example of the features that would have been extracted for the intron dataset and the UTR dataset according to the initial information shown in a.

In the next preprocessing step, for each category, the initial data were used to create three kinds of features (Fig. 3), representing different resolutions:

1. Low-resolution features—indicating the number of mutations each patient had in an entire gene.
2. Medium-resolution features—indicating the number of mutations each patient had in a 50-nucleotide-long gene segment.
3. High-resolution features—binary features indicating whether a specific mutation occurred or not, for each patient.

**Fig. 4:**
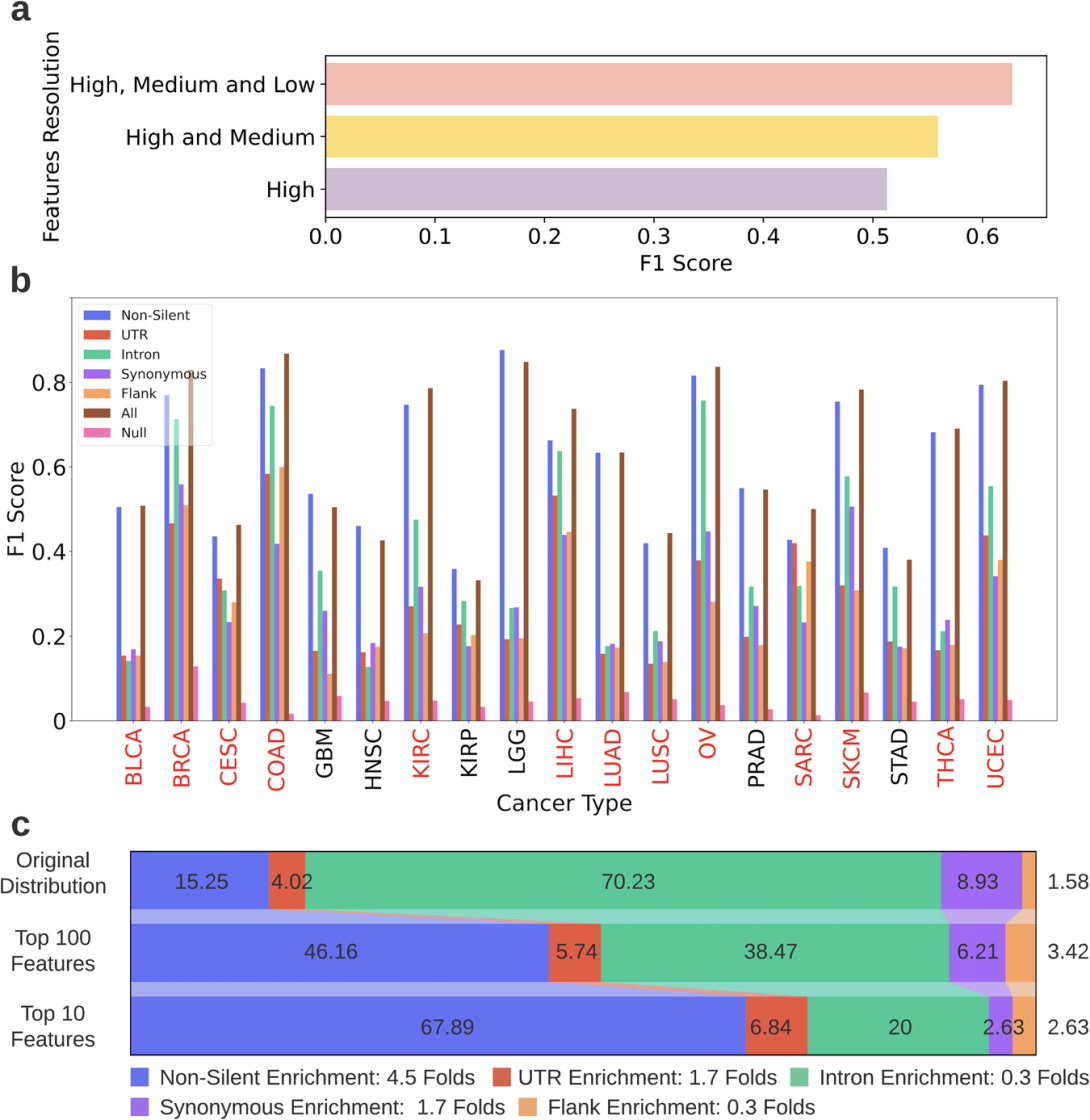
Classification task results. **a** The F1 scores achieved in the cancer type classification task when using only high-resolution features, high and medium-resolution features and all resolutions combined. The scores shown are the average F1 scores achieved by all features models across all cancer types. **b** The F1 scores achieved by the OVA models per cancer type, using features from all levels of resolution. The *x*-axis depicts the cancer types, the y-axis depicts the F1 scores achieved by the models. Each bar color denotes a different dataset. Cancer types for which the all features model outperformed the non-silent model are denoted in red. See Fig. 1e for the unabbreviated names of the cancer types. **c** Feature-type distribution of the all features dataset and of the top ranked features chosen in the classification task. Feature-type distribution of the all features dataset* (top row), top ranked 100 features (middle row) and top ranked 10 features (bottom row). The feature rankings were obtained from all features models classifying the 19 cancer types and were averaged across them. The legend (below the image) indicates the enrichment in the amount of each feature-type in the top 10 features compared to its original amount in the all features dataset (ratio between bottom and top row). *Note: The distribution depicted in the top row is the distribution of the all features dataset after it underwent preprocessing relevant for the classification task.

Analyzing features from multiple resolution levels improves the models’ results (Fig. 4a, Supplementary Table 1) and could also identify specific mutations, regulatory regions, and entire genes that are related to cancer fitness.

The features created for each of the five categories were used as five separate datasets (referred to as single-mutation-type datasets). A sixth dataset that combines features of all mutation types (referred to as all features dataset) was also created. The six datasets were used to perform cancer type diagnosis and patient survival estimation. Evaluating the performance of models trained on the six datasets enables us to compare the predictive ability of features derived from silent and non-silent mutations (referred to as silent features and non-silent features).

### For all cancer types, the silent features improved cancer classification in comparison to the null model

In the cancer type classification task, only cancer types with more than 200 patients were included (a total of 19 types). A one-vs-all (OVA), supervised learning model was created for every pair of cancer type and dataset (see Methods). Specifically, each model deployed the features in the dataset in order to predict whether patients suffered from the specific cancer type (classified as “Positive”) or suffered from any of the other types (classified as “Negative”, since the model predicts only the existence of the specific cancer). This section presents the results of this analysis.

As mentioned above, combining features from three levels of resolutions led to the best performance of cancer type classification. Figure 4b depicts the F1 scores (see Eq. (1) for the definition of the F1 score) obtained by the OVA models by using features from all levels of resolutions. The worst performing model, which used flanking-region features in order to diagnose Glioblastoma (GBM), was 1.9 folds better than the comparable null model (see Methods for details about the null models). The best performing model that used silent features was the intron model for diagnosing Ovarian Serous Cystadenocarcinoma (OV), and its F1 score was 20 folds higher than the comparable null model. Even though the non-silent models generally achieved better results than silent models, for several cancer types the performances were substantially similar. For example, for detection of Breast Invasive Carcinoma (BRCA), Liver Hepatocellular Carcinoma (LIHC) and OV the performance difference between the non-silent model and the intron model was less than 10%. For Sarcoma (SARC) diagnosis, the non-silent model outperformed the UTR model by a mere 2%, and the flank model was exceeded by only 12%. In addition, the all features models, which used both silent and non-silent features, obtained higher F1 scores than the non-silent models for 13 out of the 19 cancer types (denoted in red in Fig. 4b) and for the other cancer types, the performances were very similar.

To control for the number of features, the same analysis was conducted using balanced datasets as well (see Methods) and the results, shown in Supplementary Figure 1, accentuate the high diagnostic ability of silent mutations; In the balanced version, the Intron model outperformed the non-silent model for six cancer types and the UTR and flank models were superior to the non-silent model for two cancer types. Quite similarly to the unbalanced datasets, combining silent and non-silent mutations rather than solely using the latter improved classification results for 12 out of 19 cancer types (keeping in mind that the all features dataset had the same number of features as the non-silent dataset in this analysis). All these findings support the hypothesis that silent mutations do affect cancer mechanisms and hold additional predictive information that could not be obtained from non-silent mutations alone. Another confounder that could have influenced the classification results is the total mutational burden. To ensure that the improvement gained from adding silent features to non-silent features is not mainly due to the increase in the total mutational burden that occurs because of the addition, we examined how the increase in total mutational burden is correlated with the improvement in the F1 scores of the different cancer types (Supplementary Fig. 2). Results demonstrate a Pearson correlation of *R* = 0.38 (*p* = 0.1), indicating that only 14% of the change in the F1 score could be explained by the increase in mutational burden. So, even though the mutational burden does impact the results of classification, it is not the leading factor.

Another interesting phenomenon demonstrated in Fig. 4b is the considerable differences in the models’ ability to diagnose different cancer types. While the majority of the BRCA, LGG (Lower Grade Glioma) or COAD (Colon Adenocarcinoma) patients were correctly diagnosed (by at least one model), KIRP (Kidney Renal Papillary Cell Carcinoma) and STAD (Stomach Adenocarcinoma) patients were often poorly diagnosed. To explore the origin of this difference, we examined the similarity between genetic profiles of the different cancer types and assessed whether cancers with higher genetic similarity have higher misclassification rates: For every pair of cancer types, the correlation between their Jaccard similarity score and their misclassification rate was inspected (see Methods). The results (Supplementary Fig. 3) indicate a Spearman correlation coefficient of 0.72 (*p*-value <10^−28^), suggesting the similarity between genetic profiles of patients of different cancers is indeed a major cause for misclassifications. However, this is not the only cause as it only explains ~52% of the variance in their misclassification rate. Another factor that could lead to misclassifications is high mutation heterogeneity among patients of the same cancer type.

### Silent features comprise 32% of the 10 most predictive features for cancer classification, on average across cancer types

Each OVA model provides an importance ranking for all its features. Examining the ranking of silent features among all features is another way to evaluate their predictive power. Reviewing the feature importance ranking produced by the all features models, silent features comprised nearly half of the top ranked 100 features and a third of the top ranked 10 features (chosen from hundreds of thousands of features), when averaged across cancer types (Fig. 4c). However, the ranking of silent features varied substantially between cancer types (Supplementary Tables 2,3); while there were only non-silent features in the top 10 features of Lung Adenocarcinoma (LUAD), silent features constituted eight out of the top 10 features of Cervical Squamous Cell Carcinoma (CESC). Altogether, 18 out of the 19 cancer types had at least one silent feature in their top 10 features list, demonstrating their high significance. The analysis was repeated with balanced datasets and the results were similar (Supplementary Fig. 4).

When evaluating the influence of the polymorphism type (whether a mutation is an insertion, a deletion, or an SNP) on the importance ranking, it was seen that the presence of deletions in the highly ranked features was notably higher than their presence in the initial datasets (Supplementary Figure 5). In fact, their prevalence in the top 10 features was 2.9–6.8 folds higher than their prevalence in the initial datasets (varying between the different models). The presence of SNPs and insertions in the highly ranked features was lower than their presence in the initial datasets, with the exception of the UTR dataset, for which the insertions were 1.3 folds more common in the top 10 features lists than in the initial datasets, on average across cancer types.

### A gene’s predictive power for cancer type classification varies drastically when mutated by different types of mutations

Table 1 lists the 10 most predictive features of three of the 19 cancer types, as chosen by the all features models (Supplementary Data 1 holds the full feature importance rankings for classifying all cancer types). As seen in Table 1, some genes appeared in the top 10 ranked genes for multiple cancer types. *MUC4* was in the top 10 list for 16 out of the 19 cancer types and *TP53* was on 11 lists, suggesting these genes could play an essential role in cancer mechanisms. Interestingly, *MUC4* was predictive of many cancer types when it had either non-silent mutations or synonymous mutations. This last finding raises the following fundamental question: is the mutation type a determining factor in a gene’s ability to predict a cancer type? Or perhaps different kinds of alterations in various regions of the same gene would cause a similar loss or gain of function, leading to the same outcomes on cancer development?

**Table 1:** Examples of the top 10 ranked features for classifying various cancer types.

Cancer Type	Rank	Feature	Feature Type	Importance	Gene
CESC	0	MUC4 Non_Silent	Non-Silent	0.16	MUC4
	1	TP53 Non_Silent	Non-Silent	0.06	TP53
	2	PABPC1 UTR	UTR	0.04	PABPC1
	3	BCR UTR	UTR	0.03	BCR
	4	NF1 5602.0 UTR	UTR	0.01	NF1
	5	RGPD3 0.0 Flank	Flank	0.01	RGPD3
	6	CSF1 UTR	UTR	0.01	CSF1
	7	MUC4 Synonymous	Synonymous	0.01	MUC4
	8	SRGAP3 UTR	UTR	0.01	SRGAP3
	9	CARD11 793.0 Intron	Intron	0.01	CARD11
LIHC	0	MUC4 Non_Silent	Non-Silent	0.25	MUC4
	1	SET 210.0 Intron	Intron	0.08	SET
	2	PIK3CA Non_Silent	Non-Silent	0.03	PIK3CA
	3	ALB Intron	Intron	0.02	ALB
	4	240343-240343-chr5-Intron-DEL-T-T–	Intron	0.02	SDHA
	5	APC Non_Silent	Non-Silent	0.01	APC
	6	FAM46C UTR	UTR	0.01	FAM46C
	7	SRGAP3 UTR	UTR	0.01	SRGAP3
	8	MUC4 Synonymous	Synonymous	0.01	MUC4
	9	SEPT9 3283.0 Intron	Intron	0.01	SEPT1
THCA	0	140753336-140753336-chr7-Missense_Mutation-SNP-A-A-T	Non-Silent	0.18	BRAF
	1	BRAF 378.0 Non_Silent	Non-Silent	0.13	BRAF
	2	TP53 Non_Silent	Non-Silent	0.07	TP53
	3	MUC4 Non_Silent	Non-Silent	0.06	MUC4
	4	NRAS 189.0 Non_Silent	Non-Silent	0.02	NRAS
	5	MUC4 Silent	Synonymous	0.02	MUC4
	6	533874-533874-chr11-Missense_Mutation-SNP-T-T-C	Non-Silent	0.01	HRAS
	7	BRAF Non_Silent	Non-Silent	0.01	BRAF
	8	TP53 26.0 Non_Silent	Non-Silent	0.01	TP53
	9	LRP1B Intron	Intron	0.01	LRP1B

The top 10 feature rankings for CESC, LIHC, and THCA are shown. For each feature, the table holds its name, mutation type, its importance for classifying the specific cancer type and the gene to which it is related. The rankings were obtained from all features models.

**Fig. 5:**
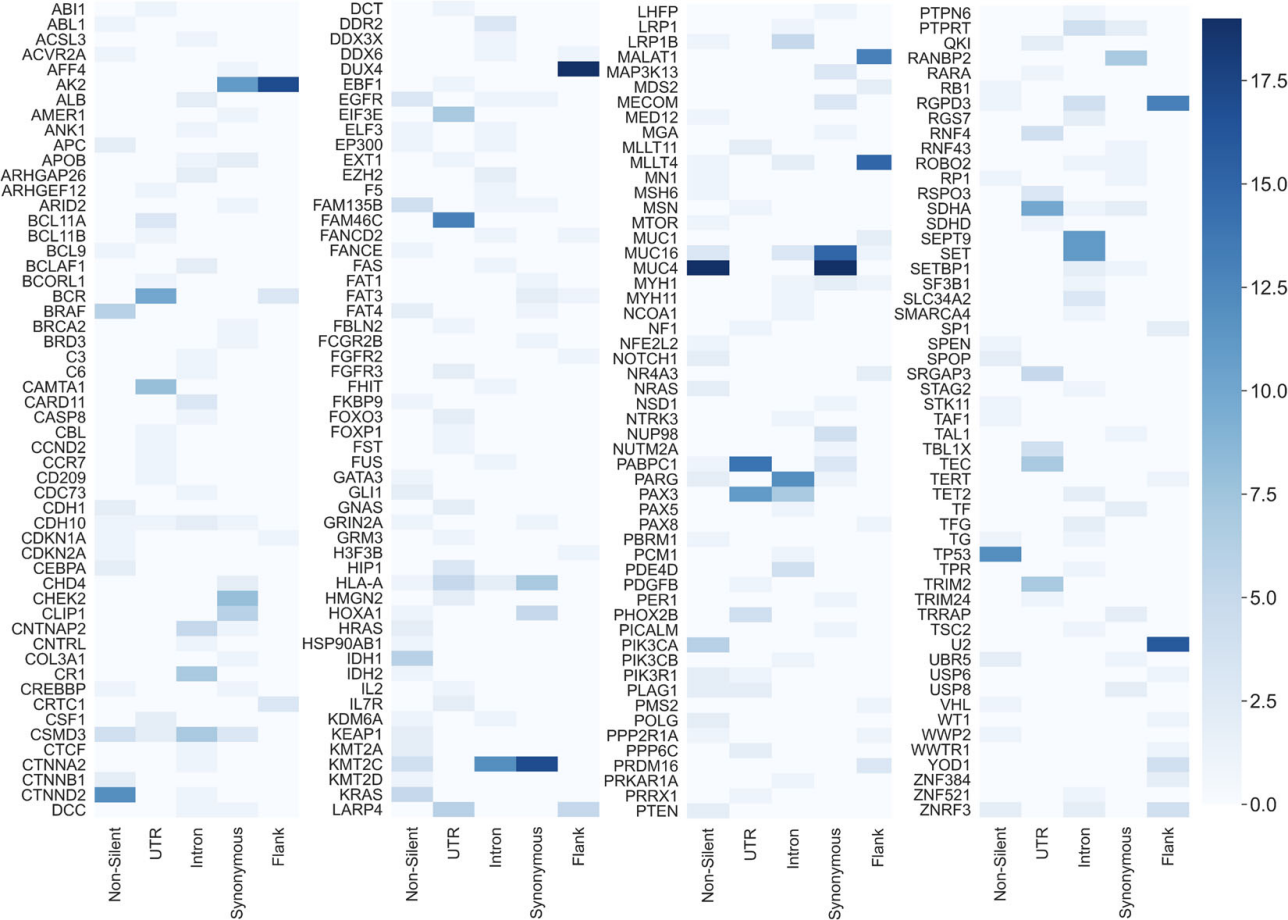
The number of top 10 ranked genes lists a gene had appeared in when it was mutated by a specific mutation type. The figure is constructed of four panels for readability purposes and is equivalent to a single long panel. Each row in a panel refers to a gene and every column in a panel refers to a mutation type. The results depicted in this figure were obtained from the five single-mutation-type models. Every gene in TCGA that is ranked in the top 10 genes list for at least one cancer type is presented in the figure (the figure includes a total of 216 genes). A lighter shade indicates that the gene was in the top 10 lists of a few cancer types and a darker shade indicates that the gene was in the top 10 lists of many cancer types. The minimum value possible is zero (the gene is not included in the top 10 genes list of any cancer type for that particular model) and the maximum is 19 (the gene is included in the top 10 genes lists for all examined cancers for that particular model).

To try and answer this question, the top 10 features list from every single-mutation-type OVA model was examined (all features models were excluded from this analysis). For each cancer, a top 10 genes list was derived from the top 10 features list (see Methods). Figure 5 depicts a heatmap, presenting the number of top 10 genes lists a gene has appeared in (19 meaning the gene appeared in the top 10 genes lists of all cancer types, and zero meaning it had appeared in none). As seen in Fig. 5, the number of appearances a gene has in the top 10 lists changes dramatically when it is mutated by mutations of different types. For example, the aforementioned *MUC4* gene appears in all 19 lists when it is mutated by non-silent mutations or synonymous mutations, but when it is mutated in the UTR, introns or flanks it loses its predictive significance and does not appear in any of the lists. In fact, it is evident that most genes are highly predictive of multiple cancer types only when mutated by a specific mutation type. For example, *MUC16* is highly predictive of 15 cancer types, but only if its mutations are synonymous. Altogether, it is evident that the mutation type does influence the predicative power a gene has on cancer diagnosis. Nonetheless, it can also be seen that for some genes, such as *AK2* or *KTM2C*, more than a single-mutation type leads to high predictivity of multiple cancers. So, even though it has been established that not all mutations cause the same effect, perhaps some lead to more similar consequences than others.

### Synonymous, non-silent and intronic mutations affect a gene’s predictive power on cancer type classification in a positively correlated manner

To assess whether some mutation types lead to similar consequences, every cancer type was separately examined. It was assumed that if two different mutation types have similar effects on a gene, then the predictive power of that gene for a specific cancer type would be similar when mutated by either one of them. Therefore, the gene’s importance in both models should be similar as well. Inferring to all genes, the gene importance ranking of both models should be correlated.

**Fig. 6:**
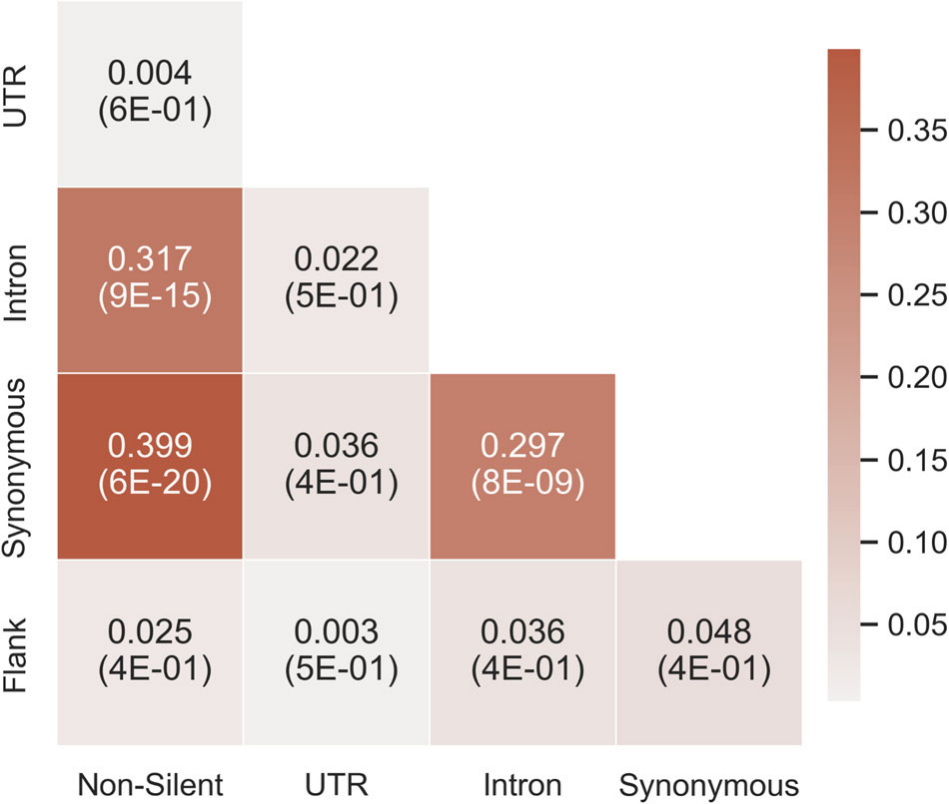
The average Spearman correlation of every pair of gene ranking lists of two models. For every cancer type, the correlation between the gene ranking lists of every pair of models was calculated. The average value across cancer types is shown. The respective average p-values are denoted in parentheses. The colors represent the correlation coefficient. A darker color indicates a higher correlation.

For every cancer type, a Spearman correlation was performed between every pair of gene ranking lists obtained from the five single-mutation-type models (see “Methods”). The correlation coefficients were then averaged across all cancer types (Supplementary Fig. 6 depicts the correlations obtained for each cancer type). The results (Fig. 6) indicate a significant 0.4 correlation between the gene ranking lists of the non-silent and synonymous models, a 0.32 correlation between the lists of the non-silent and intron models and a correlation of 0.3 between the lists of the synonymous and intron models. These three correlations obtained a *p*-value smaller than 8.5×10^−9^. Correlations between all other pairs of models were neither high nor significant. A possible reason for these results is a common mechanism shared by the different mutation types. For example, both synonymous and non-silent mutations may affect co-translational folding, and both synonymous and intronic mutations may influence splicing. Thus, it is conceivable that these mutations could have similar consequences over the gene’s expression or functionality.

### Combining both silent and non-silent features enables the detection of Gene Ontology terms that are not detected by non-silent features alone

Enrichment analysis was performed in order to examine whether genes that were considered important by the models are related to specific biological functions and processes. The affiliation of these genes to biological pathways could illuminate their contribution to the development and progression of the disease. The GOrilla^44,45^ and REVIGO^46^ tools were used to find non-redundant Gene Ontology terms (GO terms) that are enriched for any of the 19 cancer types. To find the terms, a gene ranking list was used as input for the GOrilla tool (see Methods). As demonstrated in Figs. 5, 6, different mutation types dramatically change the predictive power of genes and thus inputting gene rankings of the different models could illuminate different biological pathways.

**Fig. 7:**
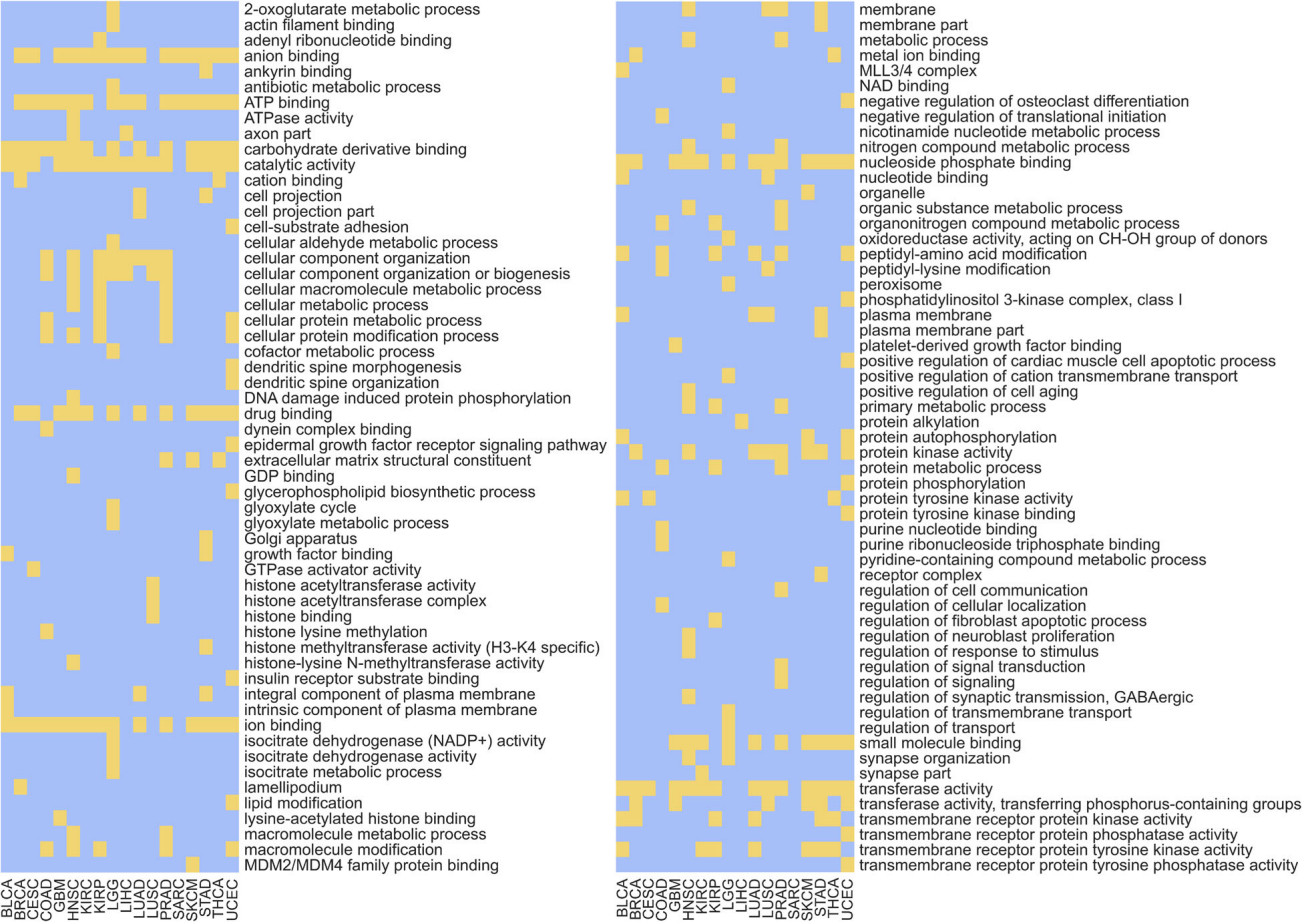
GO terms enrichment for the 19 cancer types. Received by using the gene rankings of all features models. The figure is constructed of two panels for readability purposes and is equivalent to a single long panel with 113 GO terms. Each row in a panel refers to a GO term and every column in a panel refers to a cancer type. Yellow positions indicate non-redundant enriched GO terms with a p-value smaller than 0.001 and a *q*-value (FDR correction) smaller than 0.05. Blue positions indicate GO terms that are not enriched under these requirements.

Figure 7 lists the GO terms that were enriched for the 19 cancer types when using the gene rankings from all features models. Examining these results, it can be seen that most GO terms that are repeatedly enriched across cancer types are related to DNA-protein bindings, to protein–protein bindings and to phosphorylation. As expected, these terms are associated with various regulation mechanisms of the gene expression process, such as transcription (interactions between transcription factors and RNA Polymerase, histone phosphorylation) or translation (attachment of ribosomes to the DNA sequence).

**Fig. 8:**
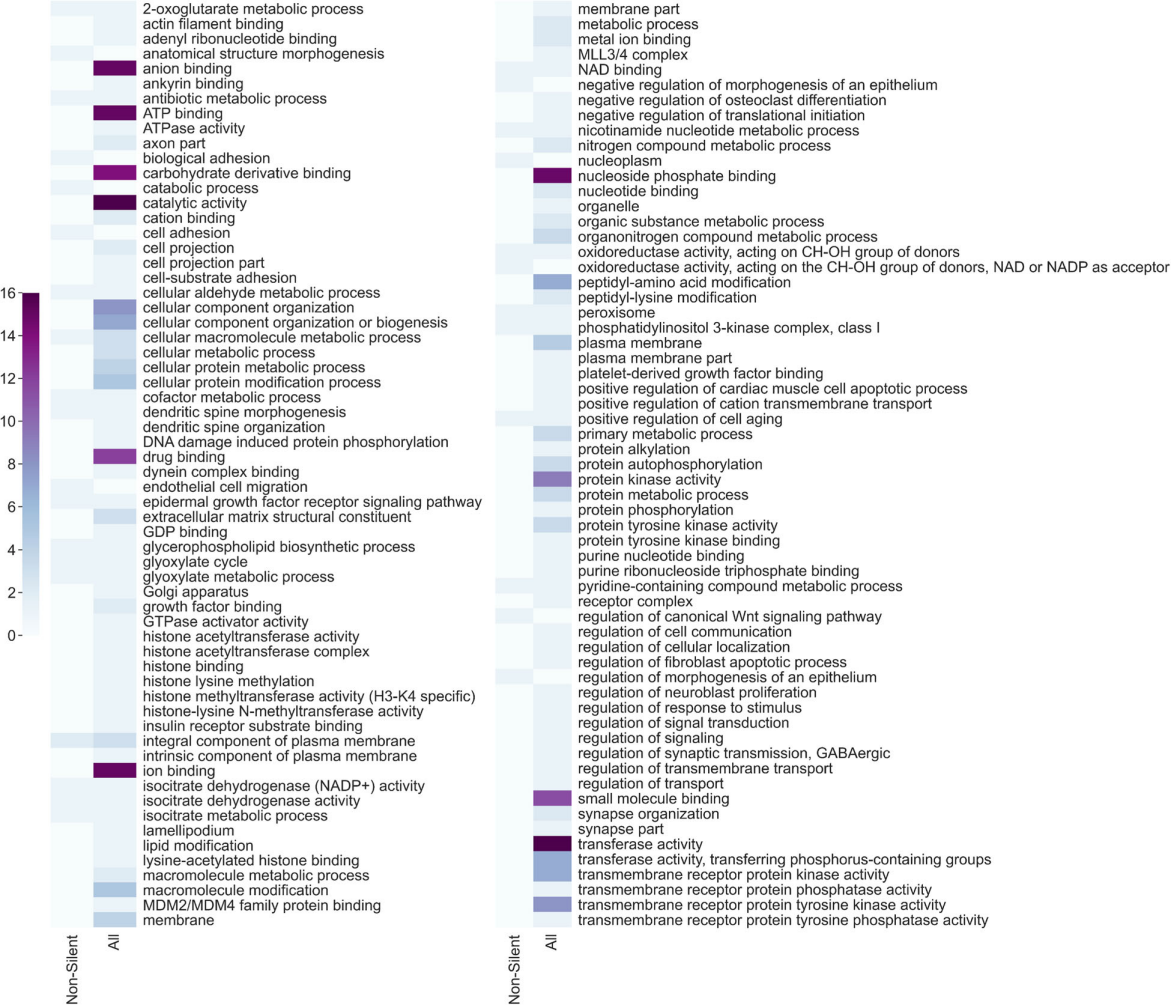
The number of cancer types for which a GO term was enriched using gene rankings from the non-silent models and all features models. The figure is constructed of two panels for readability purposes and is equivalent to a single long panel with 123 GO terms. Each row in a panel refers to a GO term and every column in a panel refers to a model from which the gene ranking list was used as input for the GOrilla tool.

As most research today encompasses mainly non-silent mutations, it is interesting to test whether the GO terms that were detected with the all features gene rankings are also detected with gene rankings obtained from non-silent models. Figure 8 depicts the number of cancer types for which a GO term was found significantly enriched when using the gene rankings from both models. It can be seen that most GO terms detected by the all features models across various cancer types are considerably less detected by the non-silent models. That is to say, adding silent features to non-silent features caused the gene ranking to encompass a broader biological significance and thus led to a more comprehensive detection of GO terms. Nonetheless, widening our prism involves a trade-off; 10 GO terms that were found significant by the non-silent model were missed by the all features model (in fact, eight of them were missed by all other models, making them unique to the non-silent model. See Supplementary Data 2). Among these terms are “endothelial cell migration” which is related to angiogenesis^47^ (a known cancer hallmark^48^), “negative regulation of morphogenesis of an epithelium” which is indeed effected in carcinoma development^49^ and “regulation of canonical Wnt signaling pathway” which is known to be profoundly related to cell tumorigenesis^50^. These terms were found significant only by the non-silent model and neither they nor semantically similar terms were detected by any other model. Even though the all features model missed these 10 terms, it did detect the other 21 terms that were found significant by the non-silent model, meaning that the majority of the information was preserved. Additionally, it detected 90 other significant GO terms that were not detected by the non-silent model. These include terms related to histone modifications (“histone binding”, “histone methyltransferase activity”, “histone acetyltransferase activity”), terms related to phosphorylation (“transmembrane receptor protein phosphatase activity”, “transmembrane receptor protein kinase activity”) and terms related to the binding of nucleic acids (“ATP binding”, “GDP binding”, “GTPase activator activity”). These biological functions and processes are known to have implications on tumorigenesis in various ways^51–54^ and none of them (or terms with similar semantic meanings) were detected by the non-silent model. We also performed pathway enrichment analysis using REACTOME^55^ (see Methods) and the results indicate that all features highly ranked genes are associated with multiple pathways related to the regulation of DNA damage. Pathways such as “Cell cycle checkpoints” (and specifically “G1/S DNA Damage Checkpoints”, “G2/M DNA damage checkpoint” and “p53-Dependent G1 DNA Damage Response”), “DNA double-strand break repair”, “SUMOylation of DNA damage response and repair proteins” and “TP53 Regulates Transcription of DNA Repair Genes” were enriched. These pathways, or any semantically similar pathways were not found enriched in the highly ranked genes of the non-silent models and are known to be profoundly related to tumorigenesis^56,57^. This further demonstrates the contribution of silent mutations to tumorigenesis and highlights the need to combine them in cancer research.

Examining the single-feature-type silent models (Supplementary Data 2), we can detect more GO terms that were unique to a specific model. For example, the term “poly(A) binding” was found significant only by the UTR model. This may suggest that poly(A) binding genes tend to undergo regulation and thus also cancer evolution through mutations in their 3’UTR which affect regulation via the changes in the poly(A) tail. The poly(A) tail is related to mRNA stability and translation regulation^58^ and alternative polyadenylation processes are known to be related to tumorigenesis^59^. Another example for a term that is unique for a specific model only is “O-glycan processing” which was found significant only by the synonymous model. The O-glycans are oligosaccharides that are a major component of mucins. The mucins function as a protective layer of the epithelium and changes in their O-glycans are related to tumorigenesis^60,61^.

The intron model also detected many significant GO terms for the various cancer types (80), only three of which (“cell adhesion”, “biological adhesion” and “integral component of plasma membrane”) are common with the non-silent model. Exactly half of the terms (40) were also detected by the all features model. To conclude, there is a trade-off in examining gene rankings obtained from single-feature-type models and models that combine several feature types. The all features model allows for a broader view of biological pathways but also misses terms that are highly specific of a certain mutation type. However, this analysis strongly indicates that searching for biological significance by only analyzing non-silent mutations is insufficient.

When examining the results depicted in Fig. 8, one must consider the uneven number of features in both models; all features models have almost seven times as many features as the non-silent models. Because the gene ranking is derived from the feature ranking it is bound to have some effect over the enrichment results. However, it is not the only determinant; if the silent features were unimportant for the model, adding them (even many of them) would not cause such a difference in the enrichment results. As the rank of a gene is derived from the rank of its most important feature (see Methods), unimportant silent features would have made a small impact on the gene ranking, leading to similar gene rankings of all features and non-silent models and thus to similar enrichment results. The fact that many more GO terms were found enriched by all features models demonstrates once again the importance of the silent features and the importance of examining the whole picture.

### All silent features models outperformed the null model in predicting survival probabilities for more than 10 years after an initial cancer diagnosis

The purpose of this analysis was to assess whether the survival probabilities of patients could be estimated solely based on their silent mutations, and to compare the estimations of the silent features models to the estimations of the non-silent and all features models. Similarly to the cancer type classification task, no additional information, such as patient’s age, sex, race, or treatment history was used. In this analysis, patients across all 33 cancer types were included and a Random Survival Forest (RSF)^62^ algorithm was utilized (see Methods). Due to the high computational requirements of the algorithm, only a subset of the features was chosen from each of the six initial datasets. The models were trained to predict patients’ survival probability at any time after an initial cancer diagnosis. Then, the models were used to estimate the survival probabilities of patients at 10 different time points. The estimations were evaluated using the Area Under the Curve (AUC)^63^ score and the results are presented in the following section.

**Fig. 9:**
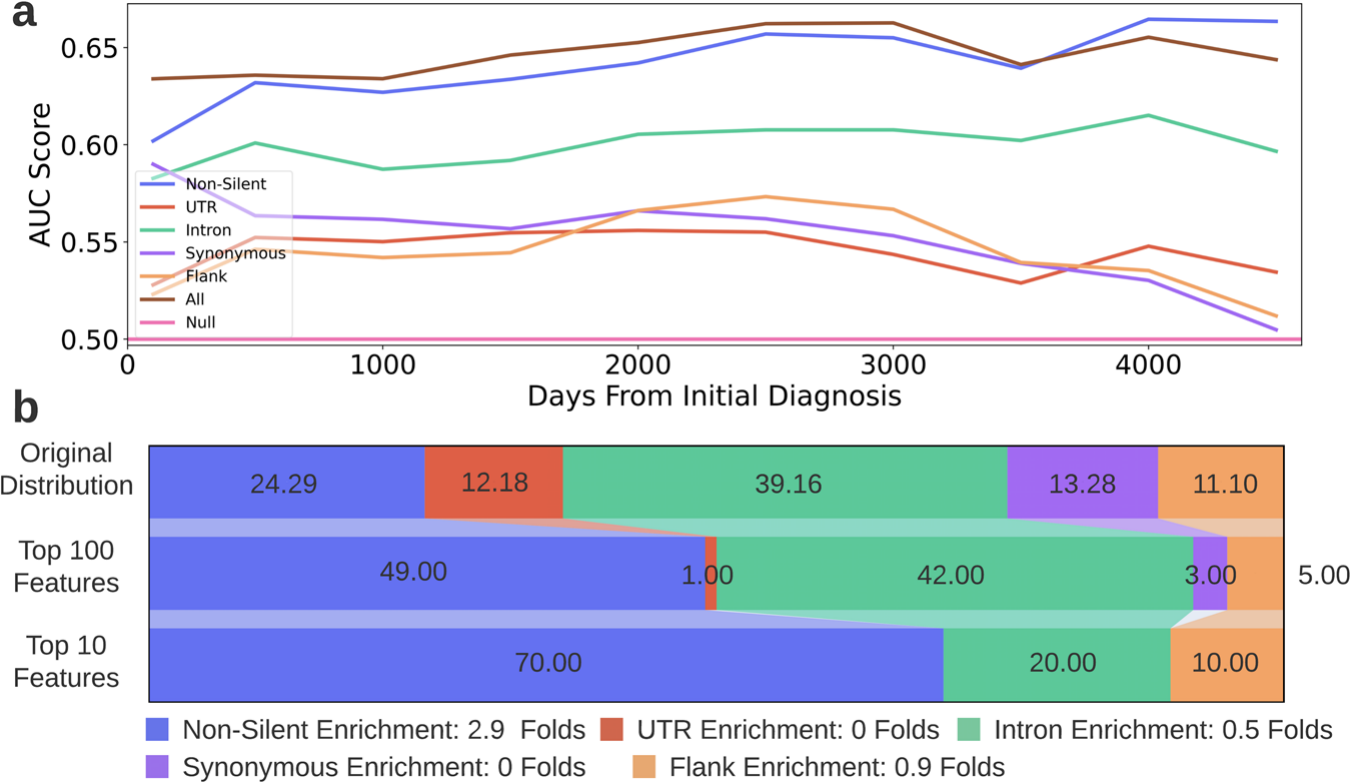
Survival estimation results. **a** AUC scores achieved by the six RSF models for various times after the initial cancer diagnosis. The *x*-axis depicts the days passed since the diagnosis and the *y*-axis depicts the AUC score achieved by the models. Each colored curve denotes a different dataset. The horizontal line depicts the AUC score of a null model. **b** Feature-type distribution of the all features dataset and of the top ranked features chosen in the survival probability estimation task. Feature-type distribution of the all features dataset* (top row), top ranked 100 features (middle row) and top ranked 10 features (bottom row). The feature rankings were obtained from the all features model. The legend indicates the enrichment in the amount of each feature-type in the top 10 features compared to its original amount in the all features dataset (ratio between bottom and top row). *Note: The distribution depicted in the top row is the distribution of the all features dataset after it underwent preprocessing relevant for the survival estimation task.

All the silent features models outperformed the null model for more than 10 years after the initial diagnosis (Fig. 9a*)*. Additionally, the all features model achieved the highest AUC score for more than nine years (3500 days) after the diagnosis. This demonstrates that the addition of silent features to non-silent features is superior to the use of non-silent features alone for survivability prediction.

### Silent features comprise 30% of the 10 most predictive features for survival estimation

Reviewing the feature importance ranking produced by the all features model for survival estimation, silent features comprised more than half of the top ranked 100 features and a third of the top ranked 10 features *(*Fig. 9a*)*. Table 2 holds the 10 most predictive features for survival estimation (and the full feature importance list is available at Supplementary Data 3). Note that due to technical reasons (see “Methods”) all patients are treated as a single cohort for the survival estimation (the cancer type of each patient is not considered by the model, only the patients’ genomic features and vital status at the last examination). If we were to perform a separate survival analysis for each cancer type as we did in the classification task, it is probable that the number of highly ranked silent mutations would vary significantly among the cancer types as seen in the previous task (Supplementary Tables 2,3). However, the fact that three of the 10 features that are most predictive of the survivability of the entire cohort are silent (even though thousands of non-silent features were available for the model’s usage), is another indicator of the strong predictive ability of silent mutations.

**Table 2:** The top 10 ranked features for estimating patients’ survival probability.

Rank	Feature	Feature type	Importance	Gene
0	TP53 Non_Silent	Non_Silent	0.0142	TP53
1	MUC4 Non_Silent	Non_Silent	0.0050	MUC4
2	57466291-57466292-chr12-Frame_Shift_Ins-INS—G	Non_Silent	0.0049	GLI1
3	143147221-143147222-chr5-Intron-INS—C	Intron	0.0035	ARHGAP26
4	140753336-140753336-chr7-Missense_Mutation-SNP-A-A-T	Non_Silent	0.0032	BRAF
5	NKX2-1 Flank	Flank	0.0031	NKX2-1
6	25743660-25743660-chr2-Missense_Mutation-SNP-T-T-G	Non_Silent	0.0027	ASXL2
7	EGFR Non_Silent	Non_Silent	0.0026	EGFR
8	92956452-92956452-chr15-Splice_Region-SNP-A-A-T	Intron	0.0026	CHD2
9	35457937-35457938-chr6-Frame_Shift_Ins-INS—C	Non_Silent	0.0026	FANCE

For each feature, the table holds its name, mutation type, its importance ranking, the gene to which it is related to and the gene’s product description. The ranking was obtained from the all features model.

## Discussion

It has been suggested that silent mutations could affect tumorigenesis and cancer cell fitness through changes in gene expression regulation^33,36–42^. However, to the best of our knowledge, this study provides the first quantitative assessment of the predictive power of silent mutations over cancer classification and prognosis in comparison to non-silent mutations.

The results demonstrate the predictive ability of silent mutations to perform both the classification and survival estimation tasks; we specifically show that for some cancer types, it is comparable to the performances of non-silent mutations. Moreover, combining both non-silent and silent mutations achieved the best classification results for 68% of the cancer types. When using the same number of features, a combination of silent and non-silent features was still superior to using only non-silent features for 63% of cancer types. Even though the survival estimation was not as comprehensive and precise as the classification task (as the patients were treated as a single cohort), the same conclusions are drawn from it; all silent feature models surpassed the null model for over ten years after an initial diagnosis and combining both silent and non-silent features led to the best survival estimations for more than 9 years. Additionally, silent features were highly ranked in both tasks, surpassing thousands of non-silent features. In fact, considering that numerous silent mutations (which affect gene expression regulation) were found highly predictive by the models and since protein functionality is quite robust to point mutations^64^, it is probable that some of the highly predictive non-silent mutations are such due to their impact on gene expression regulation rather than their impact on protein functionality. A recent study that has found similarities between the recurrency and distribution of synonymous and missense mutations also supports this claim^65^.

As shown in Fig. 4b, the predictive power of silent mutations varies significantly between cancer types. This could suggest that some cancers are more affected by changes in genes’ functionality caused mostly by non-silent mutations, while others are more affected by changes in gene expression levels, caused by both silent and non-silent mutations. The importance of different mutation types also varies when examining specific genes and pathways; the predictive power of a gene changes dramatically when it is mutated by different types of mutations. This suggests that a mutation that causes high predictivity changes the gene’s functionality or regulation in a way that is optimal for the fitness of the cancer.

Observing the feature rankings obtained by the different models, it can be seen that low-resolution features are generally ranked higher than high-resolution features (Supplementary Table 4), meaning that the number of mutations in an entire functional region of a gene was usually a better predictor than a single specific mutation. This phenomenon is noticed for both silent and non-silent features. A comprehensive understanding of the specific effect of all these mutations is a topic for future studies. However, here we provide few initial clues (see “Methods” for technical details regarding the analysis):

When examining the few silent high-resolution features that were highly ranked, we did not find that they significantly impact mRNA expression levels, splicing, or have other regulatory effects. However, when examining the low-resolution silent features that were highly ranked, we found that some contain genomic positions that are assumed to cause a disruption of regulation if mutated (Supplementary Table 5). For example, the amount of intronic mutations in the TP53 gene was the second most important feature in the all features model for detection of LUSC. We found an SNP mutation in the intronic region, 17: 7673610: T -> C, which annuls a splice site; this mutation was not highly ranked by itself, possibly due to its infrequency (present in only 0.7% of LUSC patients). A recent study showed that possible driver mutations could be missed if they are uncommon, even if they have a significant effect^35^. The TP53 gene is maybe the most known tumor suppressor^66^ and annulling of one of its splice sites could affect tumorigenesis. The number of mutations in the 3′UTR of the SRGAP3 gene was the fourth most important feature in the all features model for diagnosing SARC. We found two deletions, 3: 8985094–8985095: AT and 3: 8985094–8985097: ATAT, that both cause the formation of a new miRNA binding site. The first mutation is considerably more common than the second (present in 23.1% and 1.2% of SARC patients respectively) and was in fact the most important mutation in the entire SRGAP3 gene according to the model. The second mutation alone is ranked appreciably lower, unsurprisingly given its low prevalence. The SRGAP3 gene was also reported as a tumor suppressor gene^67^ and an addition of a new miRNA binding site could be related to tumorigenesis. The number of intronic mutations in the EGFR gene was ranked the fourth most important feature by the all features model diagnosing GBM. We found an insertion in the intronic region, 7: 55020559–55020560: ACACACAC, which causes a small but significant decrease of mRNA expression levels (0.7%). This mutation is also uncommon as it is present in only 0.7% of GBM patients. The mutations presented above affect different aspects of the regulation process of known tumor suppressors (TP53, SRGAP3) and oncogenes (EGFR), and could thus influence tumorigenesis. Generally, it seems like there could be many uncommon silent mutations with regulatory affects that are missed for lack of statistical power. With the accumulation of genomic data and improvement in computational methods, we expect that more uncommon, silent mutations that affect regulation and function will be identified. For the non-silent highly ranked features, we also did not find high-resolution features that directly affect gene expression regulation. We found only two mutations in highly ranked low-resolution features that form and revoke splice sites in the KRAS and the IDH1 genes (Supplementary Table 6).

When examining the results of this study, one should keep in mind some inherent biases of the data. For example, non-silent mutations are naturally about 20 times more frequent than synonymous mutations. Thus, even if the effect of a single mutation is similar for both types, non-silent mutations are expected to make a larger impact. Another bias originates from the source of the data; the genomic data in this study is derived using WES, which is highly biased towards exonic mutations. WES sequences the genome’s coding regions, ignoring most non-coding regions internal and external to genes^68^. In fact, an astonishing 98% of the genome is overlooked when performing WES, resulting in a narrow prism, heavily biased in favor of exonic mutations. Great efforts are made these days in order to provide data of whole genomes; The International Cancer Genome Consortium (ICGC) and The Cancer Genome Atlas (TCGA) has collaborated in the creation of the Pan-Cancer Analysis of Whole Genomes (PCAWG) and offer the ability to perform meta-analyses that includes silent mutations^35,69–74^. While it currently contains significantly smaller amounts of data and therefore a weaker statistical power compared to WES databases, it will undoubtably become a significant milestone in deciphering the contribution of silent mutations to cancer. An additional source of bias in our analyses is the varying quantity of mutations in different genes: The importance of a gene for the models is greatly influenced by the number of mutations it has in TCGA. Specifically, there is an average 0.72 Spearman correlation between the number of mutations that genes have in TCGA and the gene rankings obtained for the 19 cancer types (Supplementary Figure 7). Nonetheless, even though this correlation is high and significant, it also indicates that 52% of the variation in gene ranking could not be explained by the amount of mutations per gene in TCGA. In fact, some genes, such as *HRAS*, *YOD1*, *VHL,* and *CEBPA*, were among the most important genes for several cancer types even though their number of mutations in TCGA is very small compared to other genes (ranging from the 4^th^ to 16^th^ percentile). We expect that without these biases the significance of silent mutations in cancer diagnosis and survival prediction will be even higher than the results reported here.

Finally, this study provides a broad, statistical analysis of the predictive abilities of silent and non-silent mutations of various kinds. The results suggest that models based on silent mutations could be very useful in practice. For example, for analyzing liquid biopsy samples^75,76^ in order to perform cancer diagnosis or track cancer prognosis. Nevertheless, extensive work is required in order to expand and deepen our understanding of silent mutations and their ramifications on cancer development. For example, specific silent mutations that were chosen predictive by the models should be further investigated in order to ascertain which regulatory regions and mechanisms they impact. Novel databases containing information of silent mutations such as PCAWG and SynMICdb^65^ should be used to validate the conclusions of this study. Driver silent mutations should be distinguished from passenger silent mutations by assessing their impact on protein expression and estimating their time of occurrence. Classification should be performed on both healthy individuals and cancer patients to understand the full diagnostic ability of silent mutations. Classification should also be performed using genomic information obtained from blood samples to see whether the diagnostic ability is similar under these circumstances. Once sufficient amounts of data are available, the survival analysis should be performed again, separately for each cancer type. This is expected to improve the survival estimations and to provide greater comprehension of the silent and non-silent mutations that affect survivability. Finally, it will make sense to validate some of the mutations experimentally. All these research suggestions form the tip of the iceberg in an understudied field, full of clinical potential that is yet to be revealed.

## Methods

### Data extraction

The genomic and clinical data of patients across 33 cancer types were obtained from The Cancer Genome Atlas (TCGA)^43^. Patients with multiple genomic samples and patients with no genomic samples or clinical records were excluded, leaving a total of 9915 patients. The genomic data consists of the patients’ mutation information. A genomic position is considered mutated for a patient only if its nucleic acid content differs between the patient’s cancerous and healthy tissue samples.

### Feature engineering

Five categories of mutations were established:

1. Non-silent mutations (coding sequence mutations that cause a change in the protein’s amino-acid sequence).
2. Synonymous mutations (coding sequence mutations that do not cause a direct change in the protein’s amino-acid sequence).
3. Intronic mutations.
4. UTR mutations.
5. Flank mutations.

For each category, the genomic data obtained from TCGA was used to create three kinds of features, representing three levels of resolution (Fig. 3): low-resolution features, medium-resolution features, and high-resolution features. *Low-resolution features* count the number of mutations that appear in an entire gene. *Medium-resolution features* count the number of mutations that appear in a specific segment of a gene. Each gene is assembled from the 5′UTR, introns, exons and the 3′UTR. The flanking regions are adjacent to the gene from both ends. A gene is split to 50-nucleotide long segments and the medium-resolution features count the number of mutations in each segment. Two additional features count the number of mutations in the 5′ flanking regions (upstream to the gene) and in the 3′ flanking region (downstream to the gene). *High-resolution features* indicate whether a specific mutation occurred in a specific location in the gene (For example, an A to G SNP would be considered a different mutation than an A to C SNP, even if it had occurred in the same position). If the specific mutation occurred only for a single patient in the TCGA database, its respective feature was discarded. The features of each category were used as a separate dataset and they were also combined in order to create the sixth dataset- the all features dataset.

### One vs. all classifiers

One vs. all classifiers were chosen to perform the classification task. As our aim was to conduct a broad, quantitative comparison between various types of mutations, we chose a classic, robust, measurable, and interpretable supervised model, to lay the grounds for a fair comparison. Choosing multiple OVA classifiers, as opposed to a single multiclass classifier, enables us to easily explore which features are more closely related to which cancer type. Additionally, OVA classifiers are expected to perform better than a single multiclass classifier (as predicting a positive or negative verdict for a single cancer type is an easier task than predicting one cancer type out of 19 possibilities). Thus, if a doctor already suspects a certain cancer type, the suspicions could be validated by the relevant model with greater certainty.

To ensure enough training examples, only cancer types with more than 200 patients were included in the analysis, resulting in 8,364 patients spanning 19 cancer types. 114 OVA classifiers were generated and trained, one for each possible combination of cancer type (19) and dataset (6). The objective of each classifier was to distinguish a single cancer type from the rest. Specifically, predicting a “Positive” or “Negative” label for a particular cancer type. The OVA classifiers were constructed using the LightGBM^77^ python package. For each classifier, the patients were randomly split into stratified training and testing sets (0.7/0.3 respectively) for 10 times. A null classifier was also generated using scikit-learn’s Dummy Classifier^78^ for each cancer type; the null classifier randomly assigned labels to the test-set patients, only considering the label distribution of the training-set patients. The classifiers’ performance was evaluated with Accuracy, Recall, Precision, and F1 scores (Fig. 4b, Supplementary Table 7). Performances were averaged across the 10 splits. Precision is the fraction of correctly identified positive patients out of all patients that were identified as positive by the model. The recall is the fraction of correctly identified positive patients out of all the patients that are truly positive for the disease. The F1 score is a harmonic mean of precision and recall, taking both measures into account:

1 $${{{\mathrm{F}}}}1 = 2 \ast \frac{{P \ast R}}{{P + R}}$$

where *P* is Precision and R is Recall. The F1 score ranges from zero to one, one indicating perfect Precision and Recall scores and zero indicating that either the Precision or Recall are also zero.

### Gene ranking

Each classifier provides a feature ranking. First, features with zero importance were discarded. Then, a gene ranking was obtained by assigning the features (that can be mutations, segments, or entire genes) to the gene they are related to while keeping the original order. Finally, only the highest rank of each gene was kept. The most important gene is ranked “0” and as the numbers increase the importance decreases.

### Spearman correlation between gene rankings

Spearman correlations were conducted between gene rankings of pairs of classifiers detecting the same cancer type (Fig. 6). For every cancer type:

1. The all features classifier was excluded.
2. For each of the single-mutation-type classifiers, a gene ranking list was created as described above.
3. Every combination of two classifiers was examined; genes that were not in the intersection of both gene ranking lists were discarded. Spearman correlation was calculated between the revised gene ranking lists.

The results were averaged across the 19 cancer types.

### Gene Ontology enrichment

Enriched GO terms (molecular functions, biological processes and cellular components) were detected for the 19 cancer types using the gene rankings obtained from the different models. For every combination of cancer type and model:

1. The gene ranking list was created as described above.
2. The gene ranking list was used as input to the GOrilla tool^44,45^. The tool used maximum Hyper Geometric (mHG) statistics in order to report GO terms that are enriched in the top of the list compared to the rest of the list. The threshold for splitting the genes list to “top” and “rest” is dynamic and was chosen for each GO term individually by the tool.
3. The yielded terms are enriched with a p-value smaller than 0.001 and have passed an FDR correction of 0.05.
4. The yielded terms were used as input to the REVIGO^46^ tool, which removed terms with a semantic similarity score higher than 0.7. The similarity measure used was “SimRel”.

The enriched GO terms detected for the 19 cancer types when using the all features gene ranking are detailed in Fig. 7. A comparison between the GO terms that are detected when using the all features gene ranking or the non-silent gene ranking is seen in Fig. 8.

### Pathway enrichment

Enriched pathways were detected for the 19 cancer types using the gene rankings obtained from the different models. For every combination of cancer type and model:

1. The gene ranking list was created as described above.
2. The highest ranked 50 genes in the list were used as input to the REACTOME pathway enrichment analysis tool^55^. The number of genes was chosen considering both statistical power and the total length of the gene list.
3. The REACTOME yielded enriched pathways. An enriched pathway is a pathway for which the number of genes in the provided list that is associated to it is larger than expected by chance, considering both the total amount of genes known to be associated with the pathway and the number of gene in our list. The yielded pathways obtained an FDR value that is smaller than 0.01.

### Mutational burden

The analysis presented in Supplementary Figure 2 was conducted to evaluate whether the improvement in classification that was gained from adding silent features to non-silent features was obtained because of the additional mutational burden. For each cancer type:

1. The percent of improvement gained from adding silent features was calculated as shown in Eq. (2):
  2 $$F1_{improvement} = \frac{{F1_{all - features} - F1_{non - silent}}}{{F1_{non - silent}}} \ast 100$$
  where $F1_{all - features}$ is the F1 score of the all features model of the current cancer type.
2. The percent of mutational burden gained from adding silent features (an average across patients) was calculated as shown in Eq. (3):

3 $$MB_{increase} = \frac{{\mathop {\sum }\nolimits_{i = 1}^n \left( {\frac{{MB_{i,all - features} - MB_{i,non - silent}}}{{MB_{i,non - silent}}} \ast 100} \right)}}{n}$$

Where $MB_{i,all - features}$ is the mutational burden (number of mutations) that the $i^{th}$ patient in the all features dataset has and *n* is the number of patients of the current cancer type.

We then examined the correlation between $F1_{improvement}$ and $MB_{increase}$ among the cancer types.

### Spearman correlation between Jaccard similarity scores and misclassification rates

A Spearman correlation was conducted in order to evaluate the influence of genetic profile similarity on misclassification rates among pairs of cancer types. For this analysis binary versions of the features were used, meaning that rather than indicating how many mutations occur in genes and segments the features indicate whether any mutations had occurred or not (high-resolution features were originally binary and thus do not change). Calculating the Jaccard similarity scores for every pair of cancer types was performed in the following manner:

1. 100 patients were randomly selected from each type, forming two equally sized groups of patients (groups A and B).
2. A Jaccard score was calculated for every patient in the group A with every patient in group B. The average score was considered the Jaccard score between the groups. The calculation was performed as shown in Eq. (4):
  4 $$J_{A,B} = \frac{{\mathop {\sum}\nolimits_{a = 1}^{100} {\mathop {\sum}\nolimits_{b = 1}^{100} {} } \frac{{\left| {F_a\,\mathop { \cap }\nolimits^ F_b} \right|}}{{\left| {F_a} \right|\, +\, \left| {F_b} \right| \,-\, \left| {F_a\,\mathop { \cap }\nolimits^ F_b} \right|}}}}{{100 \ast 100}}$$
  Where *F*_*a*_ is the binary feature set of patient *a* from group A and *F*_*b*_ is the binary feature set of patient *b* from group B. $\left| {F_a} \right|$ is the number of features equal to “1” for patient *a* from group A (indicating all positions, segments and entire genes that were mutated). $J_{A,B}$ is the average Jaccard similarity score between group A and group B.
3. The random sampling process was repeated 5 times. The final Jaccard score for a pair of cancer types was the average of the five repetitions.

Calculating the mistake rate for every pair of cancer types was performed in the following manner:

1. 250 patients were randomly selected from each type (groups A and B).
2. The patients were stratified split to train and test sets (the training-set contained 70% of patients from each cancer types).
3. An OVA model was fit on the training-set patients.
4. The model was used to classify the test-set patients to one of the two cancer types.
5. The misclassification rate between the groups was calculated as shown in Eq. (5):
  5 $$M_{A,B} = \frac{{\left| {AB} \right| + \left| {BA} \right|}}{{\left| {AA} \right| + \left| {BB} \right| + \left| {AB} \right| + \left| {BA} \right|}}$$
  Where $\left| {AB} \right|$ is the number of group-A-patients that were classified as group-B-patients. *M*_*A*,*B*_ is the misclassification rate between groups A and B.
6. The random sampling process was repeated 10 times. The misclassification rate between the pair of cancer types was the average of the 10 repetitions.

### Balanced datasets

To evaluate whether the results are significantly influenced by the imbalance between the mutation categories, balanced datasets were created for the two analyses depicted in Fig. 4b and c. To maintain the balance, only high-resolution features were used in these datasets. Six same-size datasets were needed for the balanced version of Fig. 4b. For every cancer type:

1. The patients were split to two equally sized groups. The first for feature selection and creation of the balanced datasets and the second for training models on the balanced datasets and evaluating the results.
2. For creating the balanced datasets six OVA models (one per dataset) were trained using the first group of patients and all their features were ranked. For every model, the highest ranked 8,296 features were chosen as the new dataset. This step resulted in six balanced datasets per cancer type, each containing 8,296 features. (The number of features was derived from the number of features in the smallest category, the flanking region mutations).
3. The six OVA models (one per dataset) were trained using the second group of patients and the balanced datasets. The models were trained for 10 rounds, whereby on each round a stratified random 0.7/0.3 split was performed. The performance was evaluated using the same measures as the imbalanced version of this analysis.

For the balanced version of Fig. 4c an all features dataset with an internal balance between mutation types was needed. For every cancer type, the 8,296 features that were chosen from each of the five mutation categories were combined in order to create the internally balanced all features dataset. Then, an OVA model was trained using the balanced dataset and the second group of patients. The model was trained for 10 rounds, whereby on each round a stratified random 0.7/0.3 split was performed. The mutation-types distribution among the top 10 and top 100 features chosen by the classifiers were averaged across cancer types.

### Random survival forest models

A random survival forest model is an adaptation of the random forest model, modified to perform survival estimations^62^. Its performance is comparable and sometimes better than classic survival models such as Cox regression^79–82^. The RSF is a non-parametric data-driven approach that is independent of model assumptions. It was chosen for our survival estimation task because it is known to perform well specifically with high-dimensional datasets, compared to traditional approaches (for example, Cox regression relies on several assumptions that are usually violated in high-dimensional datasets)^83^.

Patients spanning all 33 cancer types were included in this analysis (as this is not a classification task and there was no need to remove small cohorts). Patients with no available information after the date of diagnosis and patients who passed away less than 20 days after their diagnosis were not included. Overall, 9,551 patients were incorporated in the analysis. The patients are treated as a single cohort and the model is oblivious of their cancer type. Unlike the classification task, this analysis is not performed separately for each cancer type because it requires more data (e.g. while the OVA model that diagnose BRCA trains on both BRCA-positive and BRCA-negative patients, the RSF model that estimates the survival of BRCA patients only trains on BRCA-positive patients while aiming at estimating an entire survival curve, and thus has a much smaller patient cohort to train on). The vital status (alive or deceased) and appropriate time stamp were extracted from the clinical data and used as labels. A subset of features was chosen for each mutation category- all low-resolution features and 5,000 high-resolution features. The high-resolution features were selected based on mutation prevalence in TCGA; the features corresponding to the 5,000 most prevalent mutations were selected.

A model was generated and trained for each one of the six datasets (non-silent, UTR, intron, synonymous, flank and all features). The objective of a model was to predict the probability of a patient to survive on a given time after its initial cancer diagnosis. The models were constructed using the Pysurvival^84^ Python package. 60 trees were grown with a maximal depth of 32 splits. At each split, Kaplan–Meier estimators and the log-rank test were used to find the feature that is the best separator. For each model, the patients were randomly split into training and testing sets (0.7/0.3 respectively). The model was trained using the training-set patients and then tested on the patients of the test set, which the model has never encountered before. To avoid biases introduced by a specific split, the process was repeated five times and the survival probability estimation is the average of the 5 repetitions.

The models’ performances on the test set patients were evaluated using the Area Under the Curve (AUC) score for various times (100, 500, 1000, 1500, 2000, 2500, 3000, 3500, 4000, and 4500 days) after the initial cancer diagnosis. After 4500 days the data is scarce, as most patients have stopped attending follow-ups or have passed away. Thus, the analysis was terminated at this point.

### Predicting the regulatory effects of highly ranked features

Predictive models were used to assess the influence of mutations spanned by the top ten ranked features of each cancer type (whether they are of low, medium or high resolution) on splice sites (using SpliceAI^85^), miRNA binding sites (using cnnMirTarget^86^), mRNA expression levels (using Xpresso^87^), polyadenylation (using SANPolyA^88^), 3D folding (using Akita^89^) and several protein-mRNA binding sites (using DeepCLIP^90^).

### Approval for study of human subjects

The need for Institutional Review Board Approval at our institution (Tel Aviv University) was waived for this study as all data used for this project had previously been generated as part of The Cancer Genome Atlas Project and none of the results reported in this manuscript can be used to identify individual patients.

### Reporting summary

Further information on research design is available in the Nature Research Reporting Summary linked to this article.

## Supplementary information

Supplementary Information

Supplementary Data 1

Supplementary Data 2

Supplementary Data 3

Reporting Summary

## Data Availability

The Clinical data and simple nucleotide variation (SNV) data that were used in this study were generated by The Cancer Genome Atlas (https://www.cancer.gov/tcga). Specifically, data of the following projects were used for the classification task: TCGA-BRCA (*n* = 1023), TCGA-UCEC (*n* = 536), TCGA-HNSC (*n* = 506), TCGA-LGG (*n* = 496), TCGA-PRAD (*n* = 493), TCGA-LUAD (*n* = 488), TCGA-THCA (*n* = 486), TCGA-SKCM (*n* = 468), TCGA-STAD (*n* = 441), TCGA-LUSC (*n* = 435), TCGA-BLCA (*n* = 408), TCGA-COAD (*n* = 402), TCGA-LIHC (*n* = 373), TCGA-OV (*n* = 372), TCGA-KIRC (*n* = 308), TCGA-CESC (*n* = 303), TCGA-GBM (*n* = 292), TCGA-KIRP (*n* = 283) and TCGA-SARC (*n* = 251). Data of these aforementioned projects and the following projects were used for the survival estimation task: TCGA-ESCA (*n* = 183), TCGA-PAAD (*n* = 182), TCGA-PCPG (*n* = 175), TCGA-READ (*n* = 151), TCGA-THYM (*n* = 123), TCGA-ACC (*n* = 92), TCGA-MESO (*n* = 83), TCGA-UVM (*n* = 80), TCGA-KICH (*n* = 66), TCGA-UCS (*n* = 57), TCGA-DLBC (*n* = 48) and TCGA-CHOL (*n* = 45). The data were downloaded from the Genomic Data Commons (https://portal.gdc.cancer.gov/) in December 2018.

## Code availability

The code will be provided upon a reasonable request for noncommercial usage.
