# Supplementary material for: Estimating the predictive power of silent mutations on cancer classification and prognosis
Source: NPJ Genom Med. 2021 Aug 12;6:67. doi: 10.1038/s41525-021-00229-1 (PMC8361094; doi:10.1038/s41525-021-00229-1)
Supplement: Supplementary file 1 — Supplementary Information [file 41525_2021_229_MOESM1_ESM.pdf]

Supplementary Figures

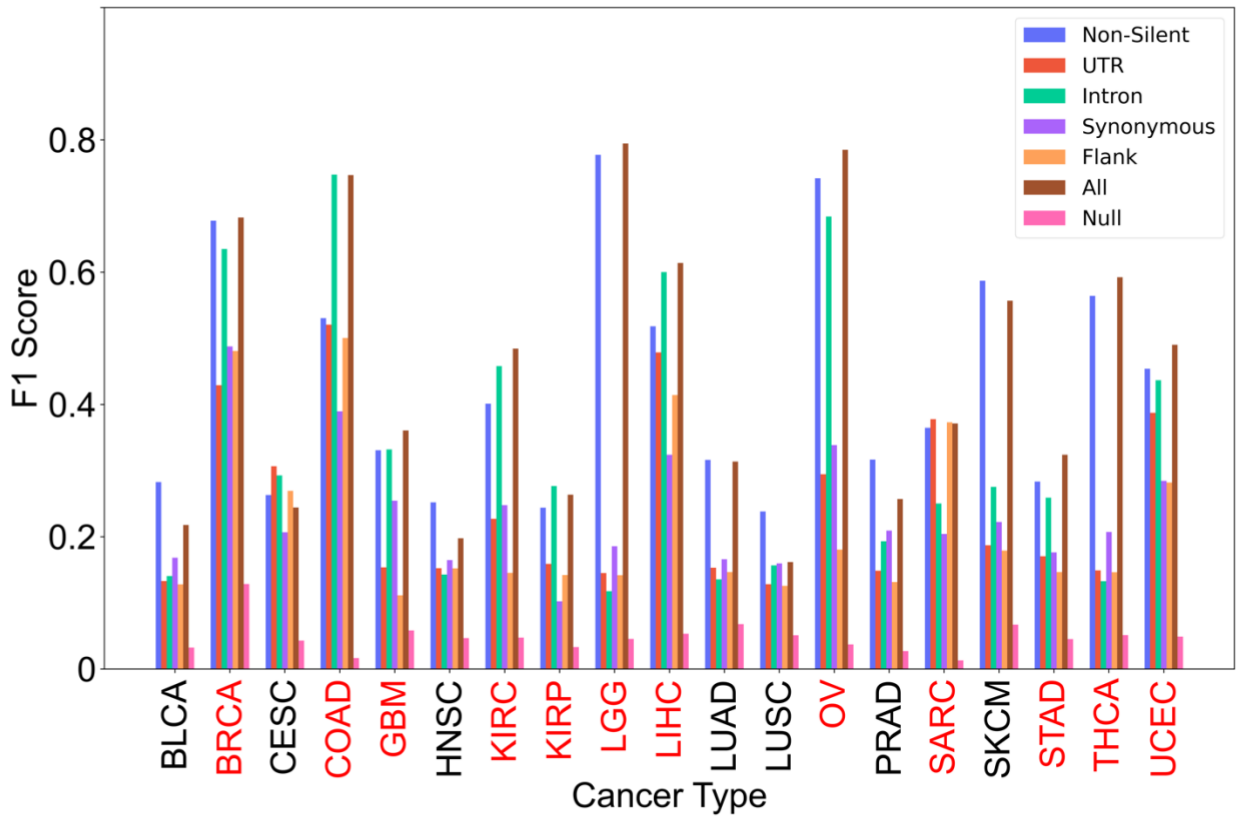

Supplementary Figure 1: The F1 scores achieved by the OVA models using the balanced datasets. Features from all levels of resolution were used. Each dataset contained 8,296 features. The x axis depicts the cancer types, the y-axis depicts the F1 scores achieved by the models. Each bar color denotes a different dataset. Cancer types for which the all-features classifier outperformed the non-silent classifier are denoted on red.

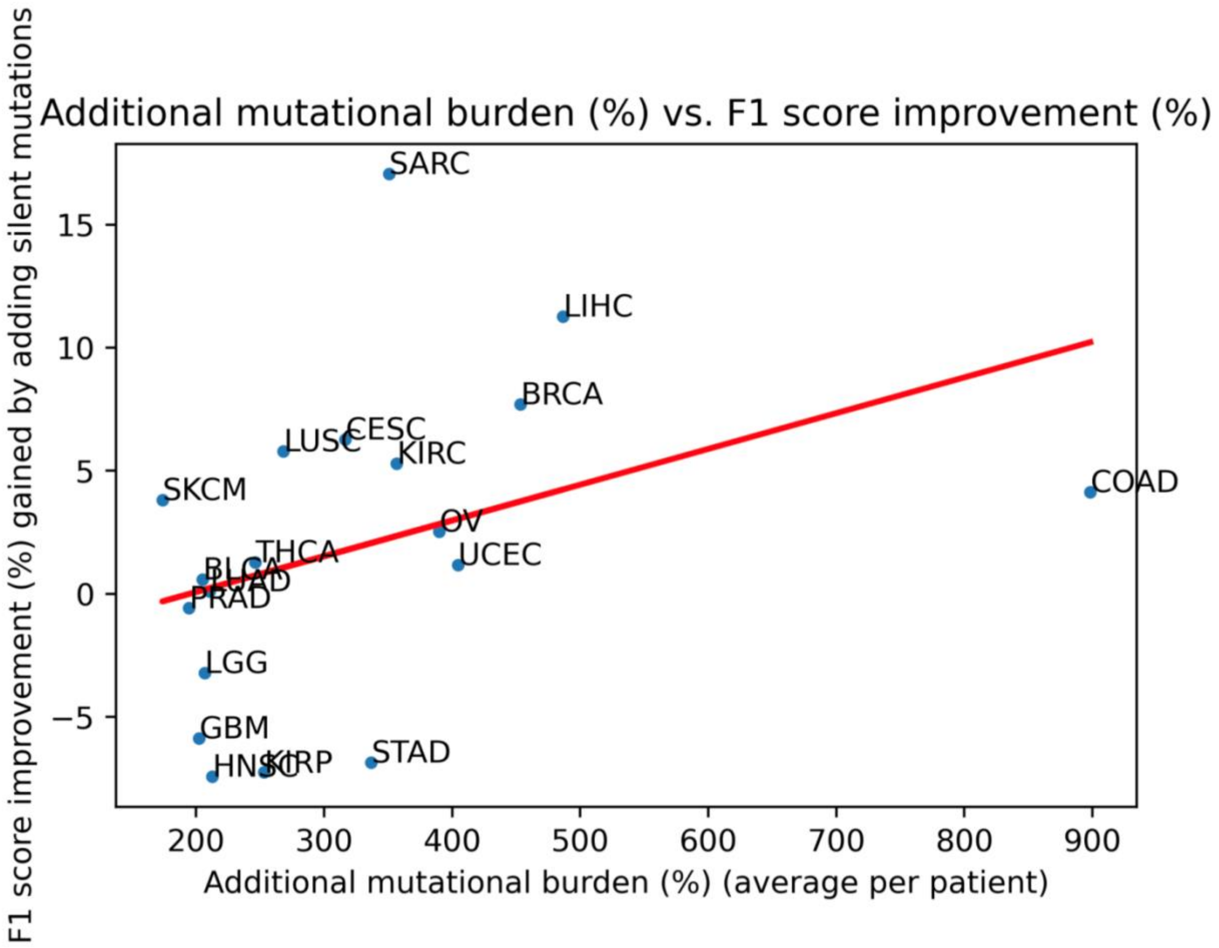

Supplementary Figure 2: The correlation between the increase in mutational burden and the F1 score improvement obtained by adding silent features to non-silent features. The x-axis depicts the percentage of additional mutational burden that was added on average per patient when adding silent features to non-silent features. The y-axis depicts the percent of improvement gained in F1 score by adding silent features to non-silent features. Every dot represents a single cancer type.

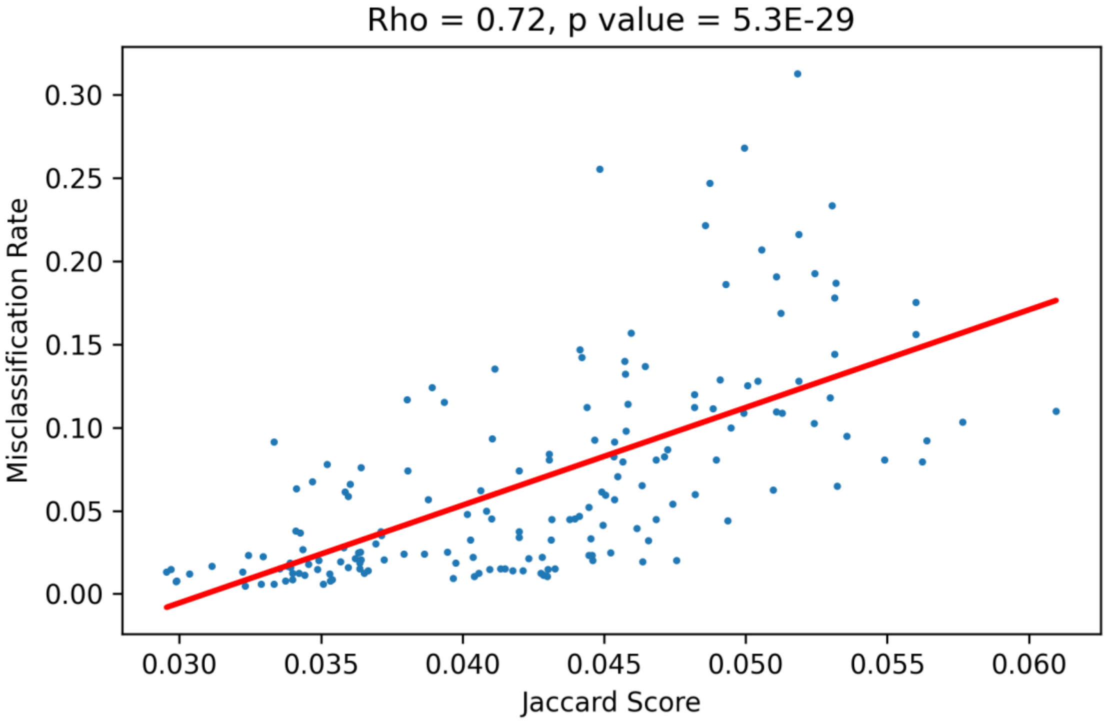

Supplementary Figure 3: Spearman correlation between Jaccard similarity scores and misclassification rates of pairs of cancer types. Every dot represents a pair of cancer types. The x axis denotes the pair's Jaccard similarity score and the y axis denotes their misclassification rate. The Spearman coefficient (Rho) and respective p value are noted above each graph.

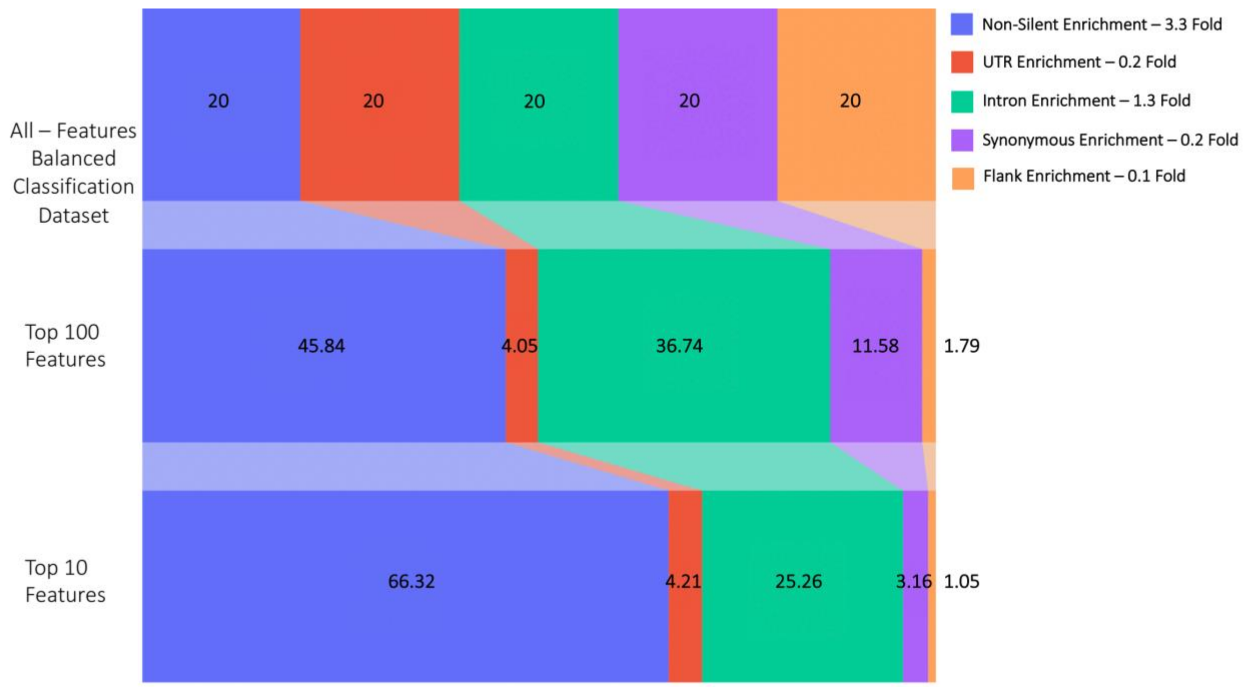

Supplementary Figure 4: Feature-type distribution of the balanced all-features dataset and of the top ranked features for the classification task. Feature-type distribution of the all-features dataset (top row), top ranked 100 features (middle row) and top ranked 10 features (bottom row). The feature rankings were obtained from the all-features models and were averaged across cancer types. The legend indicates the enrichment in the amount of each feature-type in the top 10 features when compared to its original amount in the balanced all-features dataset (ratio between bottom and top row).

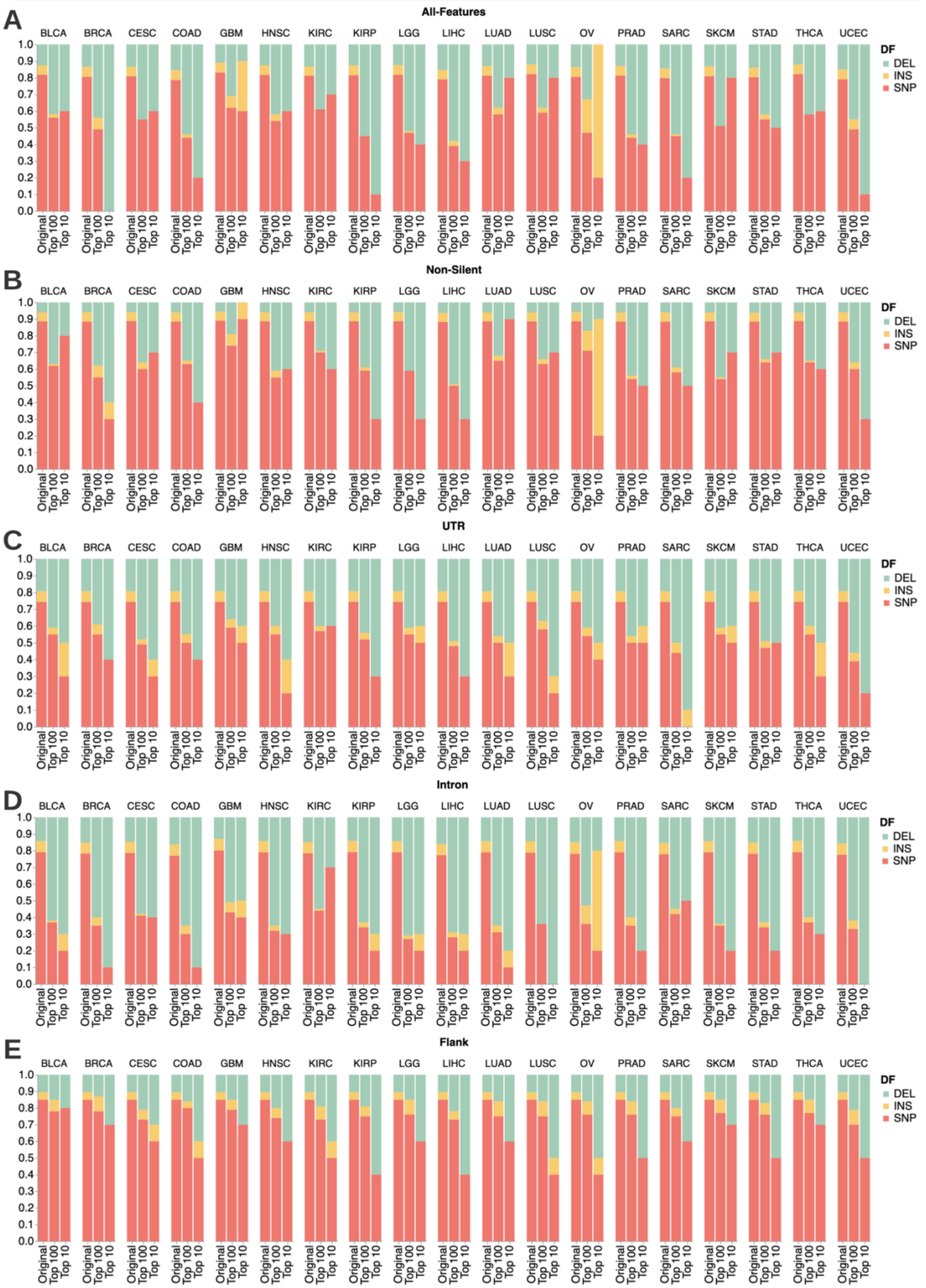

A
All-Features
B
Non-Silent
C
UTR
D
Intron
E
Flank
BLCA
BRCA
CESC
COAD
GBM
HNSC
KIRC
KIRP
LGG
LIHC
LUAD
LUSC
OV
PRAD
SARC
SKCM
STAD
THCA
UCEC
Original
Top 100
Top 10
DF
DEL
INS
SNP

Supplementary Figure 5: **Polymorphism type distributions in the initial datasets, top 100 features and top 10 features obtained from the OVA models.** Each sub-figure (a-b) denotes a model. Within a sub-figure, every three clustered columns represent the distribution of the initial dataset (left column), top 100 features (middle column) and top 10 features (right column) of a single cancer type. The analysis was conducted using the feature importance rankings that were obtained from the balanced datasets. The Synonymous models contain only SNPs and thus are excluded from this analysis.

a.

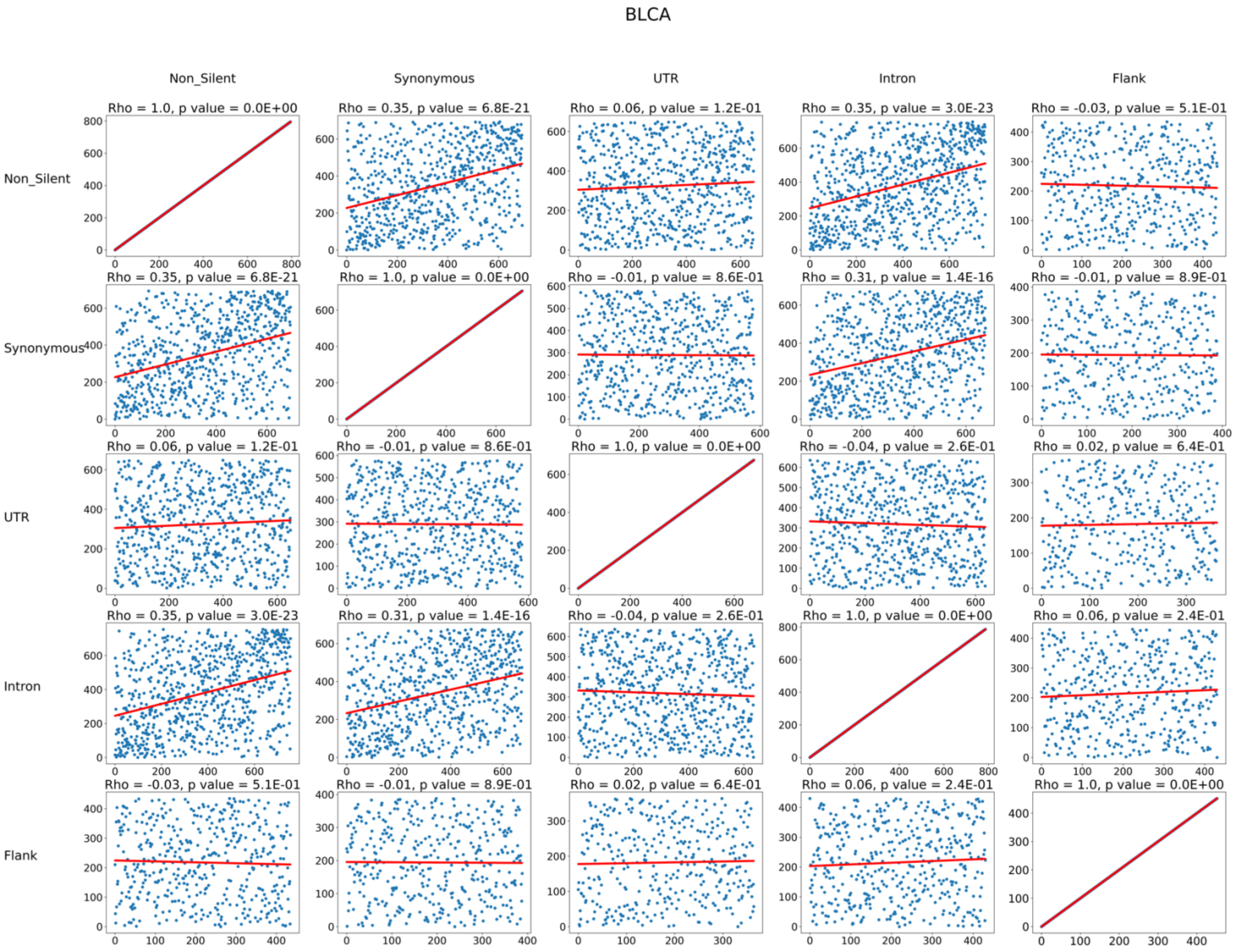

b.

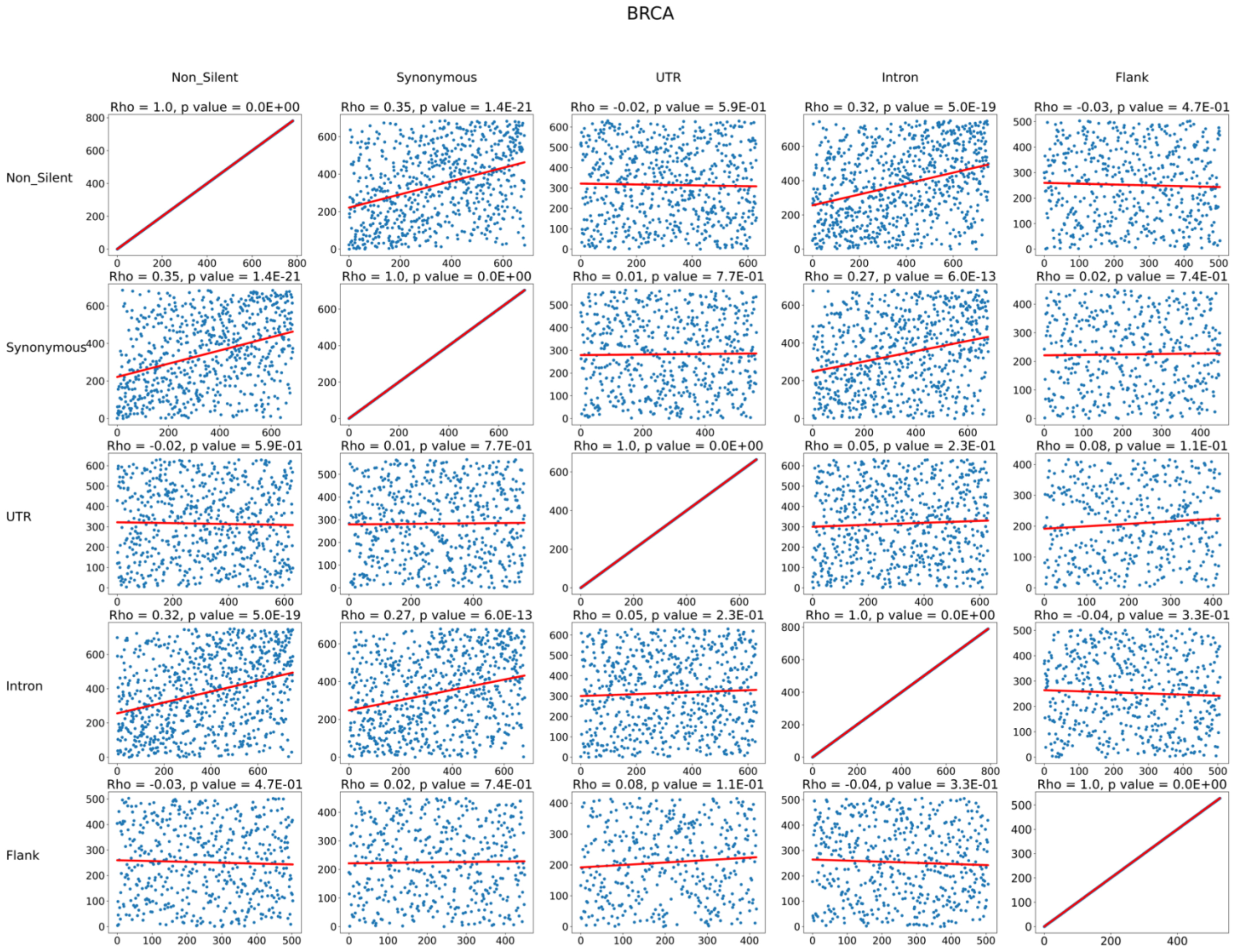
BRCA
Non_Silent
Synonymous
UTR
Intron
Flank
Rho = 1.0, p value = 0.0E+00
Rho = 0.35, p value = 1.4E-21
Rho = -0.02, p value = 5.9E-01
Rho = 0.32, p value = 5.0E-19
Rho = -0.03, p value = 4.7E-01
Rho = 0.35, p value = 1.4E-21
Rho = 1.0, p value = 0.0E+00
Rho = 0.01, p value = 7.7E-01
Rho = 0.27, p value = 6.0E-13
Rho = 0.02, p value = 7.4E-01
Rho = -0.02, p value = 5.9E-01
Rho = 0.01, p value = 7.7E-01
Rho = 1.0, p value = 0.0E+00
Rho = 0.05, p value = 2.3E-01
Rho = 0.08, p value = 1.1E-01
Rho = 0.32, p value = 5.0E-19
Rho = 0.27, p value = 6.0E-13
Rho = 0.05, p value = 2.3E-01
Rho = 1.0, p value = 0.0E+00
Rho = -0.04, p value = 3.3E-01
Rho = -0.03, p value = 4.7E-01
Rho = 0.02, p value = 7.4E-01
Rho = 0.08, p value = 1.1E-01
Rho = -0.04, p value = 3.3E-01
Rho = 1.0, p value = 0.0E+00

c.

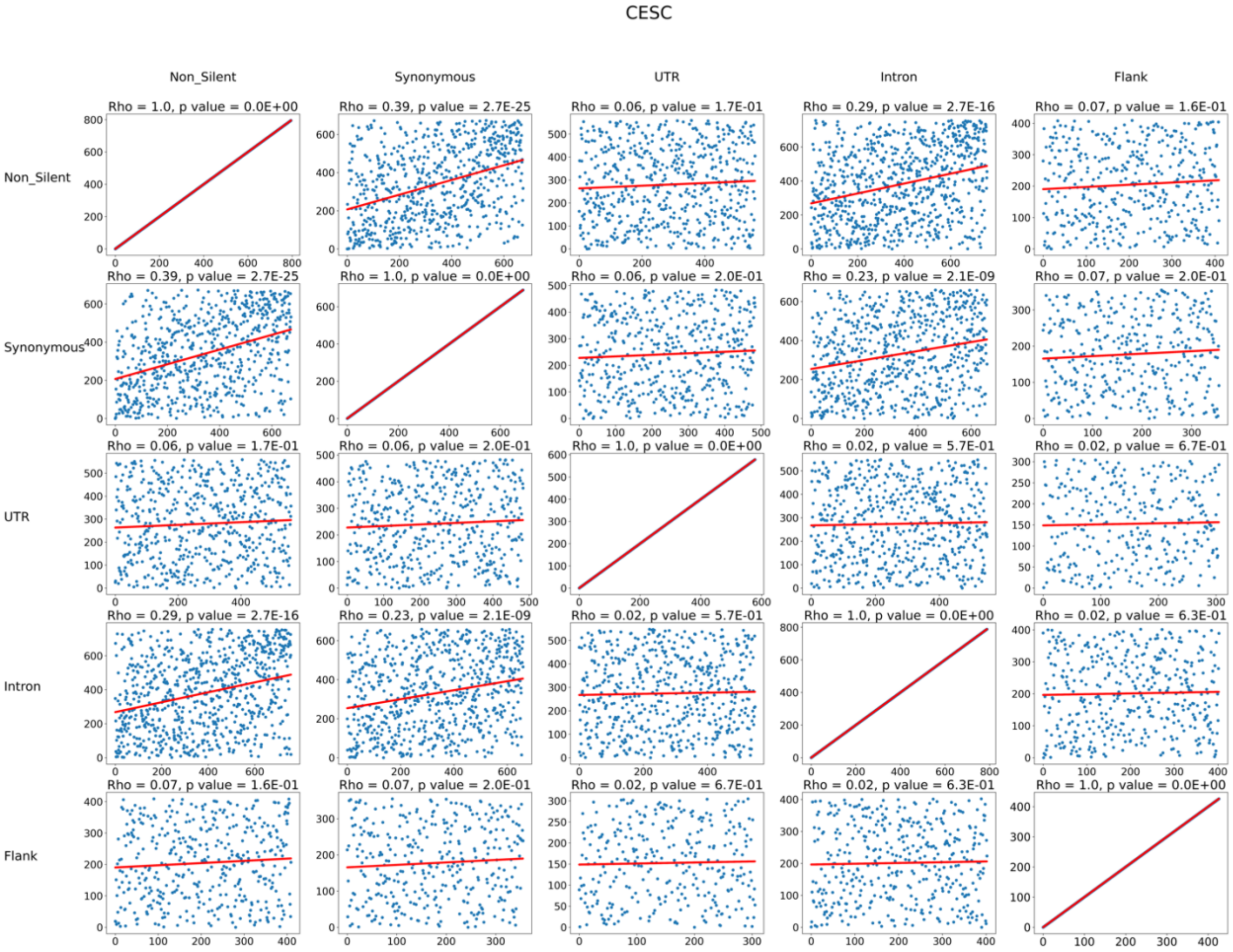
CESC
Non_Silent
Synonymous
UTR
Intron
Flank
Rho = 1.0, p value = 0.0E+00
Rho = 0.39, p value = 2.7E-25
Rho = 0.06, p value = 1.7E-01
Rho = 0.29, p value = 2.7E-16
Rho = 0.07, p value = 1.6E-01
Rho = 0.39, p value = 2.7E-25
Rho = 1.0, p value = 0.0E+00
Rho = 0.06, p value = 2.0E-01
Rho = 0.23, p value = 2.1E-09
Rho = 0.07, p value = 2.0E-01
Rho = 0.06, p value = 1.7E-01
Rho = 0.06, p value = 2.0E-01
Rho = 1.0, p value = 0.0E+00
Rho = 0.02, p value = 5.7E-01
Rho = 0.02, p value = 6.7E-01
Rho = 0.29, p value = 2.7E-16
Rho = 0.23, p value = 2.1E-09
Rho = 0.02, p value = 5.7E-01
Rho = 1.0, p value = 0.0E+00
Rho = 0.02, p value = 6.3E-01
Rho = 0.07, p value = 1.6E-01
Rho = 0.07, p value = 2.0E-01
Rho = 0.02, p value = 6.7E-01
Rho = 0.02, p value = 6.3E-01
Rho = 1.0, p value = 0.0E+00

d.

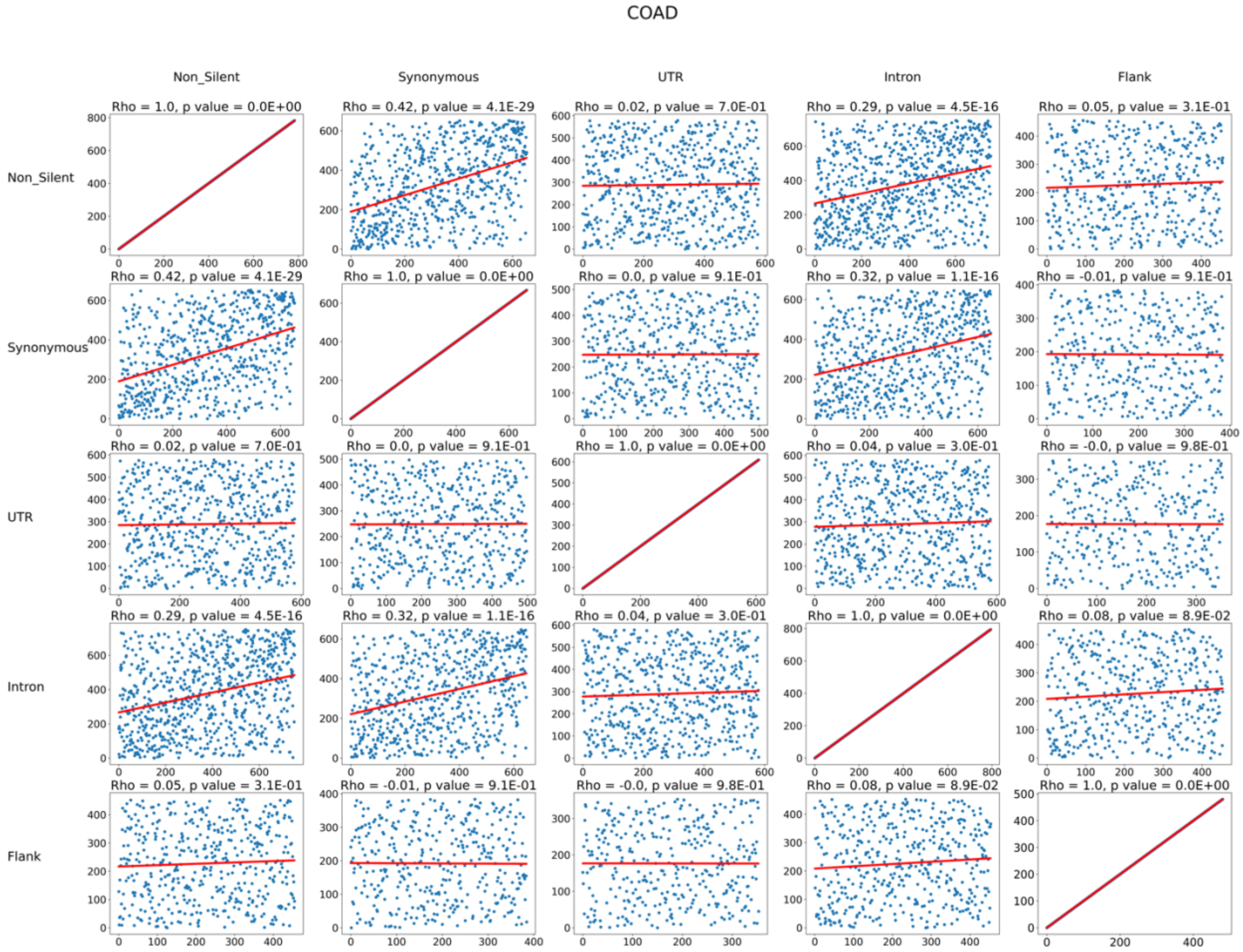
COAD
Non_Silent
Synonymous
UTR
Intron
Flank
Rho = 1.0, p value = 0.0E+00
Rho = 0.42, p value = 4.1E-29
Rho = 0.02, p value = 7.0E-01
Rho = 0.29, p value = 4.5E-16
Rho = 0.05, p value = 3.1E-01
Rho = 0.42, p value = 4.1E-29
Rho = 1.0, p value = 0.0E+00
Rho = 0.0, p value = 9.1E-01
Rho = 0.32, p value = 1.1E-16
Rho = -0.01, p value = 9.1E-01
Rho = 0.02, p value = 7.0E-01
Rho = 0.0, p value = 9.1E-01
Rho = 1.0, p value = 0.0E+00
Rho = 0.04, p value = 3.0E-01
Rho = -0.0, p value = 9.8E-01
Rho = 0.29, p value = 4.5E-16
Rho = 0.32, p value = 1.1E-16
Rho = 0.04, p value = 3.0E-01
Rho = 1.0, p value = 0.0E+00
Rho = 0.08, p value = 8.9E-02
Rho = 0.05, p value = 3.1E-01
Rho = -0.01, p value = 9.1E-01
Rho = -0.0, p value = 9.8E-01
Rho = 0.08, p value = 8.9E-02
Rho = 1.0, p value = 0.0E+00

e.

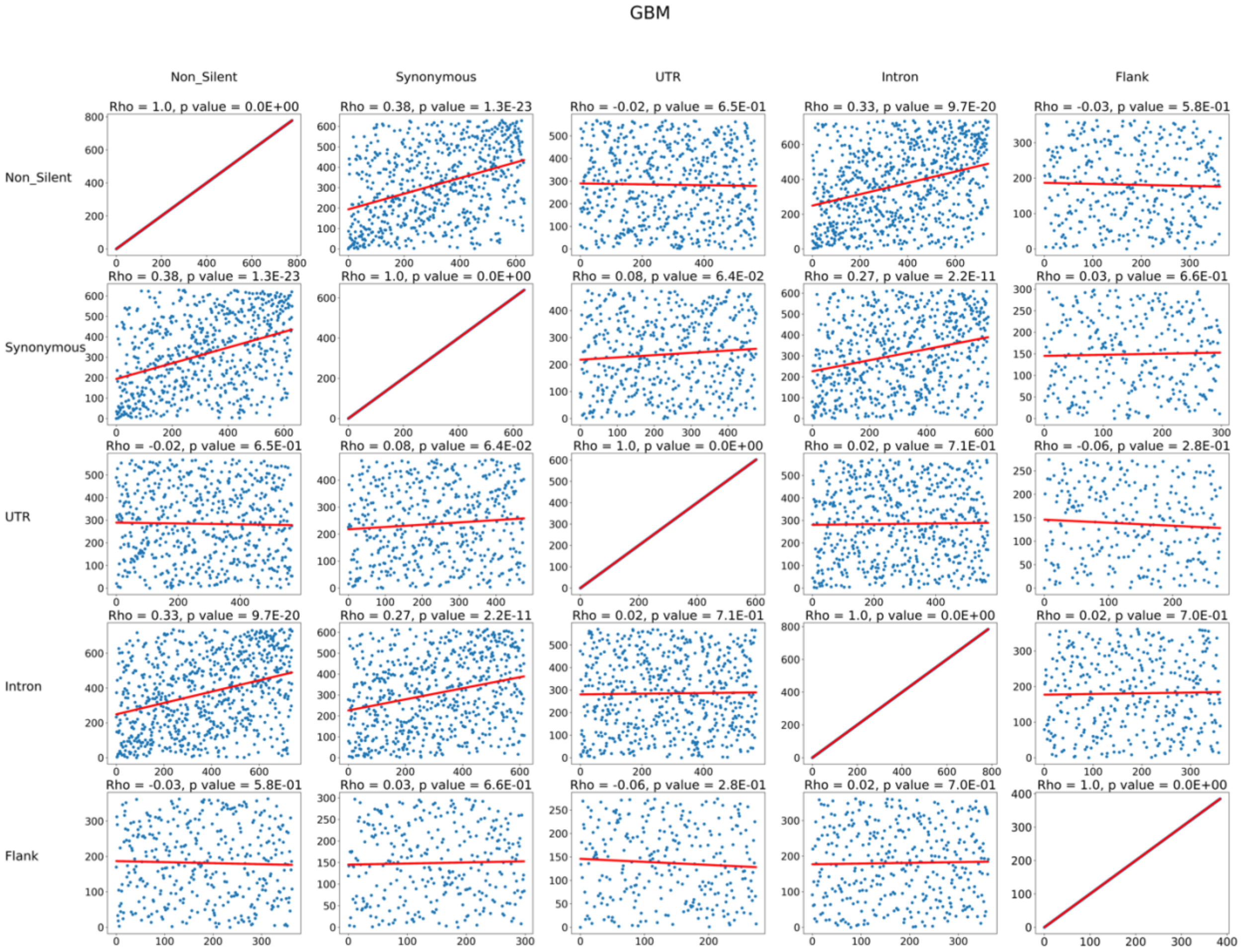
GBM
Non_Silent
Synonymous
UTR
Intron
Flank
Rho = 1.0, p value = 0.0E+00
Rho = 0.38, p value = 1.3E-23
Rho = -0.02, p value = 6.5E-01
Rho = 0.33, p value = 9.7E-20
Rho = -0.03, p value = 5.8E-01
Rho = 0.38, p value = 1.3E-23
Rho = 1.0, p value = 0.0E+00
Rho = 0.08, p value = 6.4E-02
Rho = 0.27, p value = 2.2E-11
Rho = 0.03, p value = 6.6E-01
Rho = -0.02, p value = 6.5E-01
Rho = 0.08, p value = 6.4E-02
Rho = 1.0, p value = 0.0E+00
Rho = 0.02, p value = 7.1E-01
Rho = -0.06, p value = 2.8E-01
Rho = 0.33, p value = 9.7E-20
Rho = 0.27, p value = 2.2E-11
Rho = 0.02, p value = 7.1E-01
Rho = 1.0, p value = 0.0E+00
Rho = 0.02, p value = 7.0E-01
Rho = -0.03, p value = 5.8E-01
Rho = 0.03, p value = 6.6E-01
Rho = -0.06, p value = 2.8E-01
Rho = 0.02, p value = 7.0E-01
Rho = 1.0, p value = 0.0E+00

f.

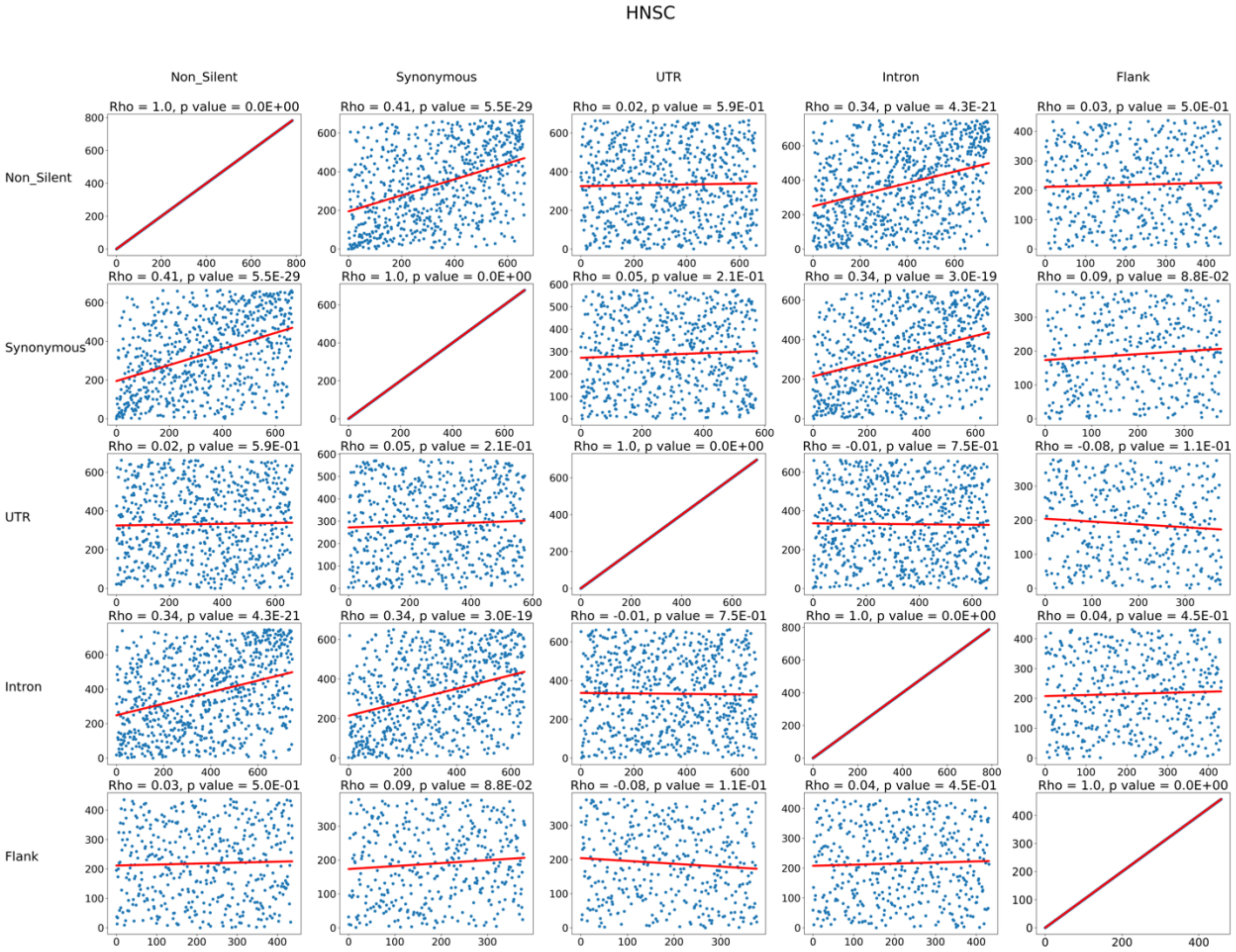

HNSC
Non_Silent
Synonymous
UTR
Intron
Flank
Rho = 1.0, p value = 0.0E+00
Rho = 0.41, p value = 5.5E-29
Rho = 0.02, p value = 5.9E-01
Rho = 0.34, p value = 4.3E-21
Rho = 0.03, p value = 5.0E-01
Rho = 0.41, p value = 5.5E-29
Rho = 1.0, p value = 0.0E+00
Rho = 0.05, p value = 2.1E-01
Rho = 0.34, p value = 3.0E-19
Rho = 0.09, p value = 8.8E-02
Rho = 0.02, p value = 5.9E-01
Rho = 0.05, p value = 2.1E-01
Rho = 1.0, p value = 0.0E+00
Rho = -0.01, p value = 7.5E-01
Rho = -0.08, p value = 1.1E-01
Rho = 0.34, p value = 4.3E-21
Rho = 0.34, p value = 3.0E-19
Rho = -0.01, p value = 7.5E-01
Rho = 1.0, p value = 0.0E+00
Rho = 0.04, p value = 4.5E-01
Rho = 0.03, p value = 5.0E-01
Rho = 0.09, p value = 8.8E-02
Rho = -0.08, p value = 1.1E-01
Rho = 0.04, p value = 4.5E-01
Rho = 1.0, p value = 0.0E+00

g.

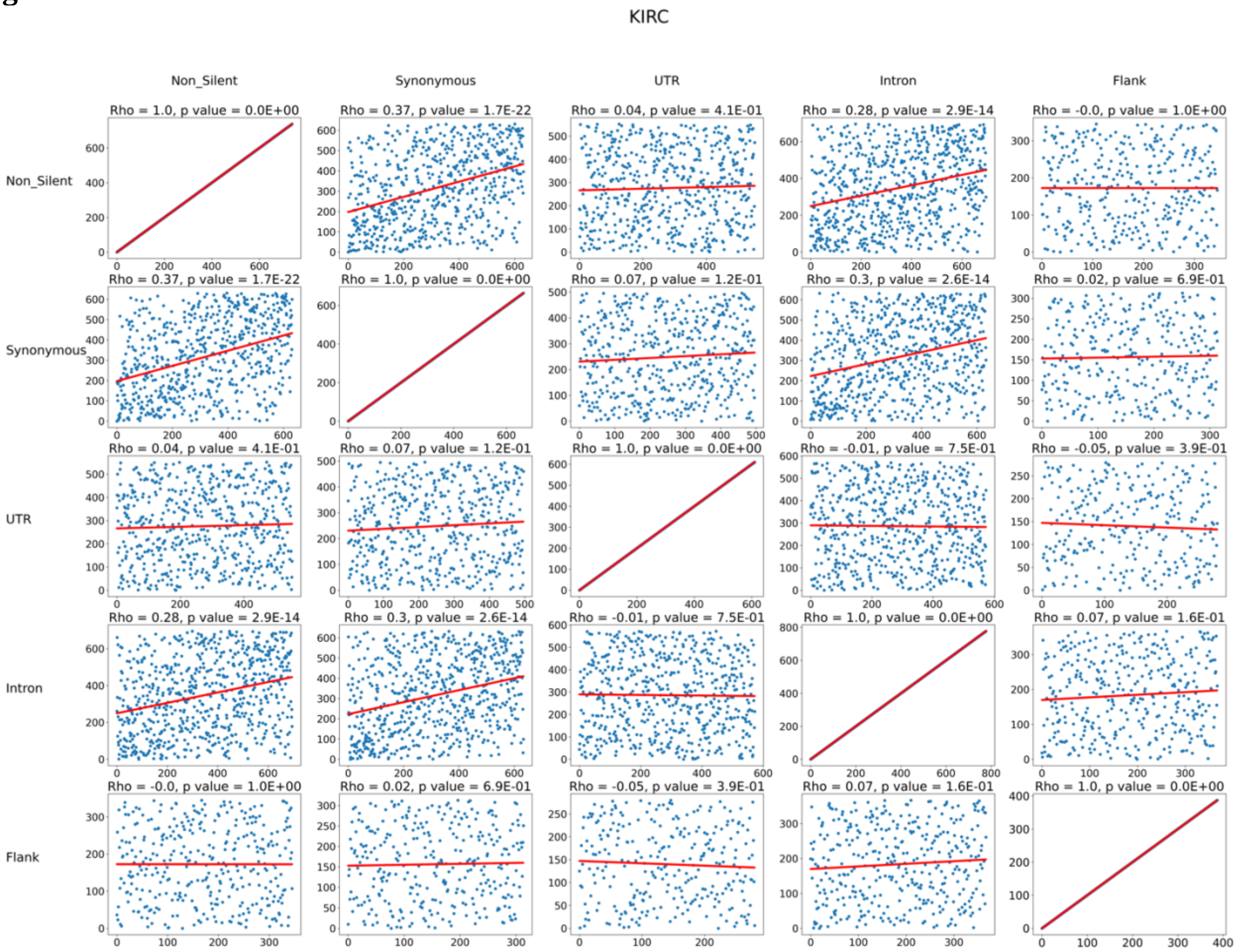
KIRC
Non_Silent
Synonymous
UTR
Intron
Flank
Rho = 1.0, p value = 0.0E+00
Rho = 0.37, p value = 1.7E-22
Rho = 0.04, p value = 4.1E-01
Rho = 0.28, p value = 2.9E-14
Rho = -0.0, p value = 1.0E+00
Rho = 0.37, p value = 1.7E-22
Rho = 1.0, p value = 0.0E+00
Rho = 0.07, p value = 1.2E-01
Rho = 0.3, p value = 2.6E-14
Rho = 0.02, p value = 6.9E-01
Rho = 0.04, p value = 4.1E-01
Rho = 0.07, p value = 1.2E-01
Rho = 1.0, p value = 0.0E+00
Rho = -0.01, p value = 7.5E-01
Rho = -0.05, p value = 3.9E-01
Rho = 0.28, p value = 2.9E-14
Rho = 0.3, p value = 2.6E-14
Rho = -0.01, p value = 7.5E-01
Rho = 1.0, p value = 0.0E+00
Rho = 0.07, p value = 1.6E-01
Rho = -0.0, p value = 1.0E+00
Rho = 0.02, p value = 6.9E-01
Rho = -0.05, p value = 3.9E-01
Rho = 0.07, p value = 1.6E-01
Rho = 1.0, p value = 0.0E+00

h.

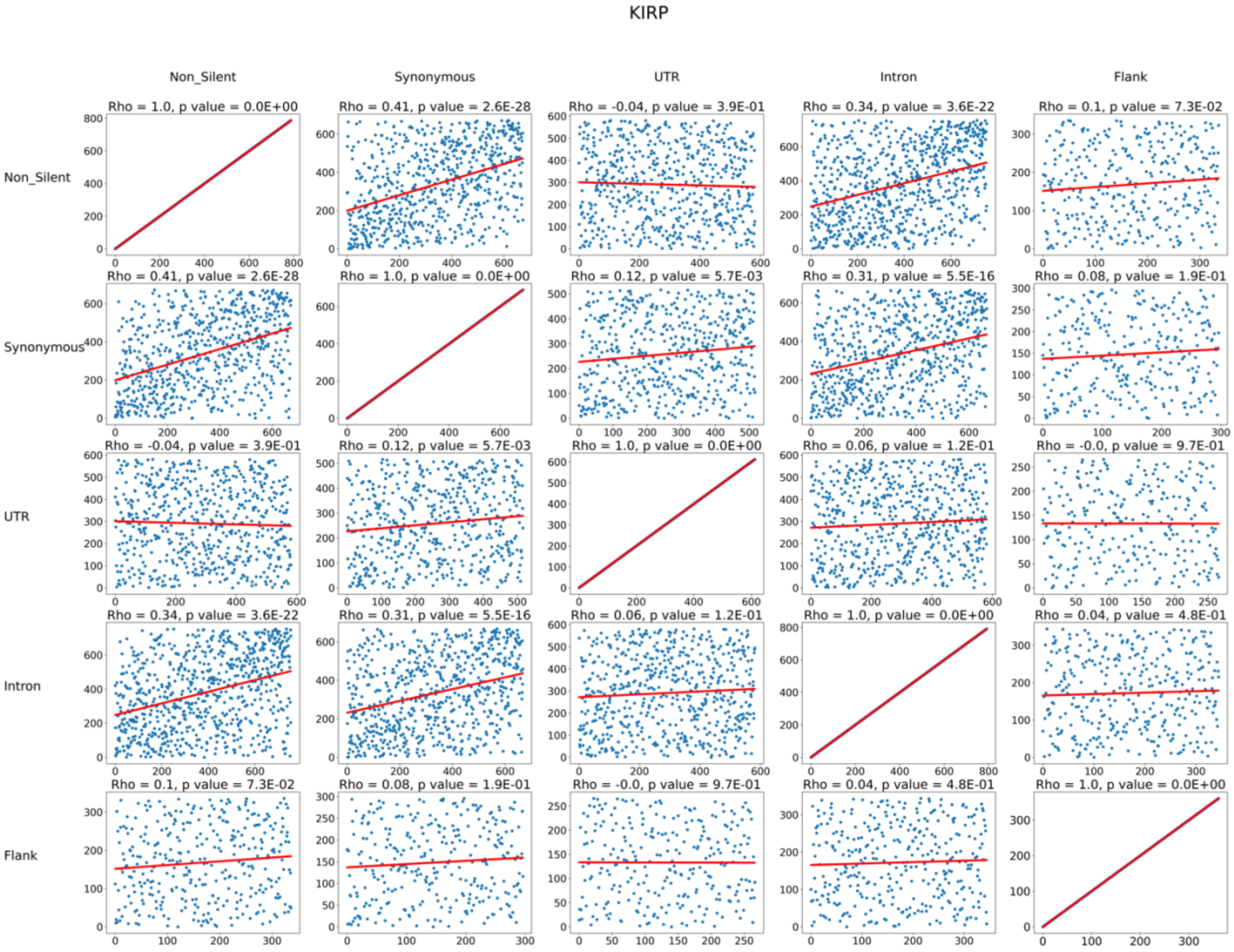
KIRP
Non_Silent
Synonymous
UTR
Intron
Flank
Rho = 1.0, p value = 0.0E+00
Rho = 0.41, p value = 2.6E-28
Rho = -0.04, p value = 3.9E-01
Rho = 0.34, p value = 3.6E-22
Rho = 0.1, p value = 7.3E-02
Rho = 0.41, p value = 2.6E-28
Rho = 1.0, p value = 0.0E+00
Rho = 0.12, p value = 5.7E-03
Rho = 0.31, p value = 5.5E-16
Rho = 0.08, p value = 1.9E-01
Rho = -0.04, p value = 3.9E-01
Rho = 0.12, p value = 5.7E-03
Rho = 1.0, p value = 0.0E+00
Rho = 0.06, p value = 1.2E-01
Rho = -0.0, p value = 9.7E-01
Rho = 0.34, p value = 3.6E-22
Rho = 0.31, p value = 5.5E-16
Rho = 0.06, p value = 1.2E-01
Rho = 1.0, p value = 0.0E+00
Rho = 0.04, p value = 4.8E-01
Rho = 0.1, p value = 7.3E-02
Rho = 0.08, p value = 1.9E-01
Rho = -0.0, p value = 9.7E-01
Rho = 0.04, p value = 4.8E-01
Rho = 1.0, p value = 0.0E+00

i.

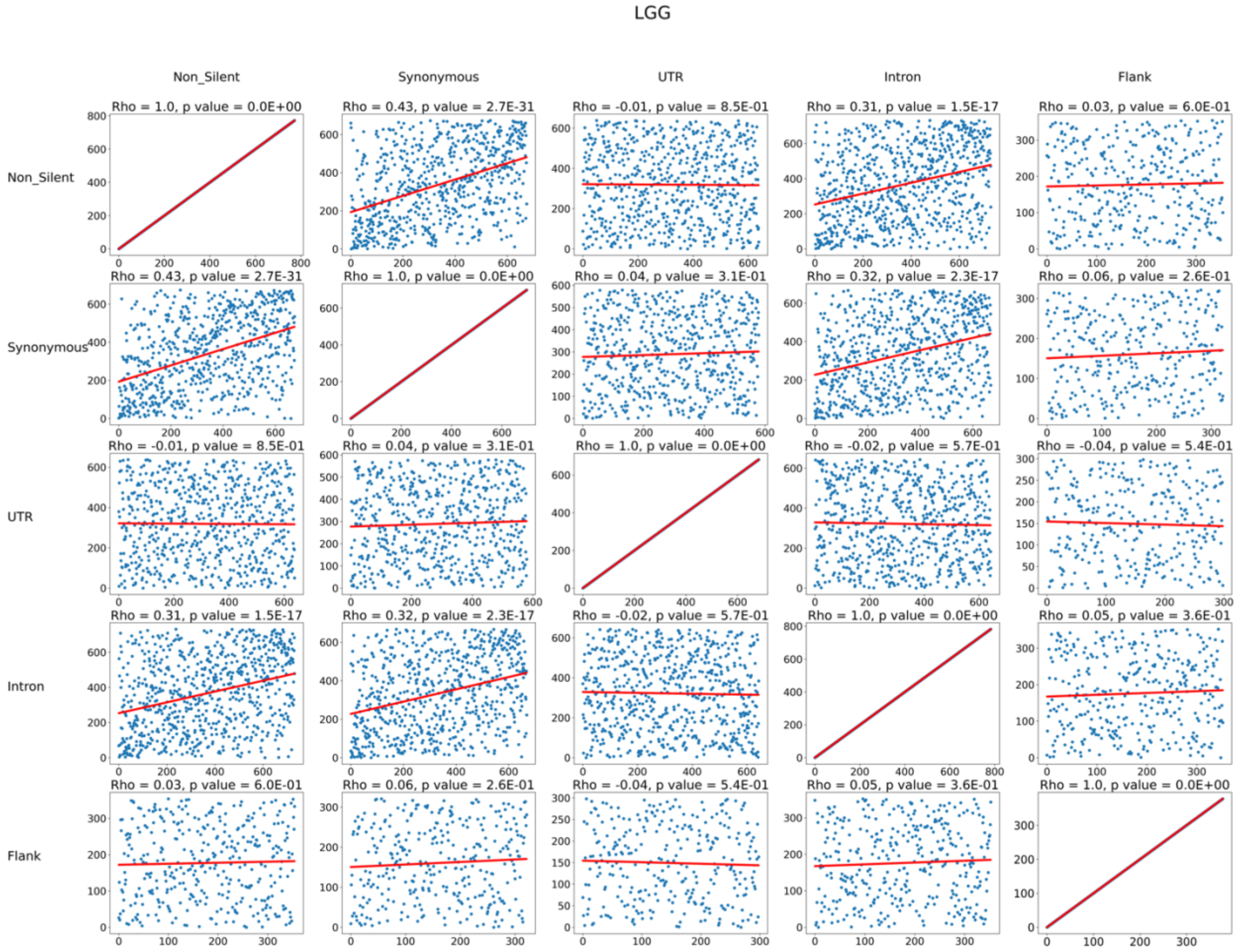
LGG
Non_Silent
Synonymous
UTR
Intron
Flank
Rho = 1.0, p value = 0.0E+00
Rho = 0.43, p value = 2.7E-31
Rho = -0.01, p value = 8.5E-01
Rho = 0.31, p value = 1.5E-17
Rho = 0.03, p value = 6.0E-01
Rho = 0.43, p value = 2.7E-31
Rho = 1.0, p value = 0.0E+00
Rho = 0.04, p value = 3.1E-01
Rho = 0.32, p value = 2.3E-17
Rho = 0.06, p value = 2.6E-01
Rho = -0.01, p value = 8.5E-01
Rho = 0.04, p value = 3.1E-01
Rho = 1.0, p value = 0.0E+00
Rho = -0.02, p value = 5.7E-01
Rho = -0.04, p value = 5.4E-01
Rho = 0.31, p value = 1.5E-17
Rho = 0.32, p value = 2.3E-17
Rho = -0.02, p value = 5.7E-01
Rho = 1.0, p value = 0.0E+00
Rho = 0.05, p value = 3.6E-01
Rho = 0.03, p value = 6.0E-01
Rho = 0.06, p value = 2.6E-01
Rho = -0.04, p value = 5.4E-01
Rho = 0.05, p value = 3.6E-01
Rho = 1.0, p value = 0.0E+00

j.

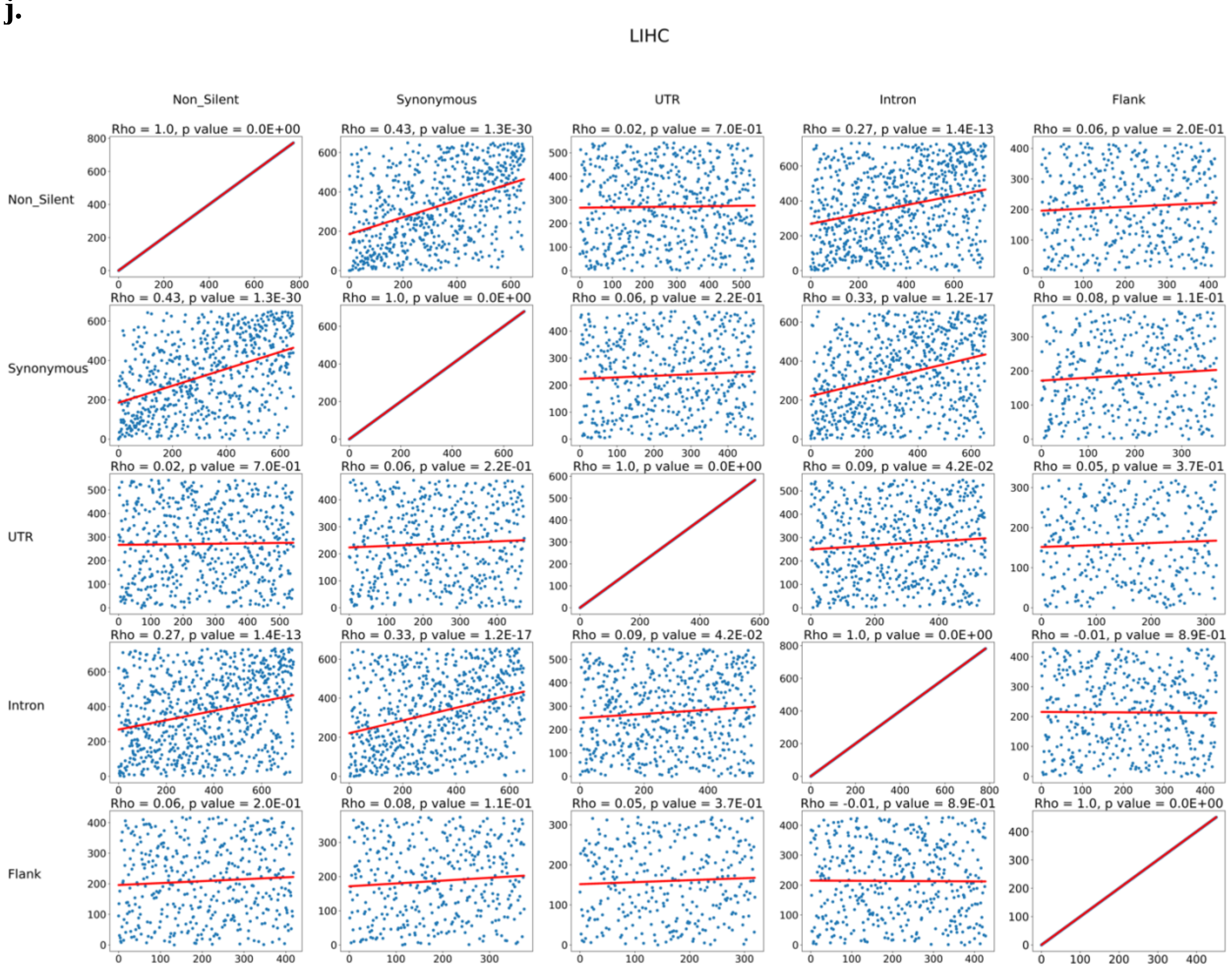
LIHC
Non_Silent
Synonymous
UTR
Intron
Flank
Rho = 1.0, p value = 0.0E+00
Rho = 0.43, p value = 1.3E-30
Rho = 0.02, p value = 7.0E-01
Rho = 0.27, p value = 1.4E-13
Rho = 0.06, p value = 2.0E-01
Rho = 0.43, p value = 1.3E-30
Rho = 1.0, p value = 0.0E+00
Rho = 0.06, p value = 2.2E-01
Rho = 0.33, p value = 1.2E-17
Rho = 0.08, p value = 1.1E-01
Rho = 0.02, p value = 7.0E-01
Rho = 0.06, p value = 2.2E-01
Rho = 1.0, p value = 0.0E+00
Rho = 0.09, p value = 4.2E-02
Rho = 0.05, p value = 3.7E-01
Rho = 0.27, p value = 1.4E-13
Rho = 0.33, p value = 1.2E-17
Rho = 0.09, p value = 4.2E-02
Rho = 1.0, p value = 0.0E+00
Rho = -0.01, p value = 8.9E-01
Rho = 0.06, p value = 2.0E-01
Rho = 0.08, p value = 1.1E-01
Rho = 0.05, p value = 3.7E-01
Rho = -0.01, p value = 8.9E-01
Rho = 1.0, p value = 0.0E+00

k.

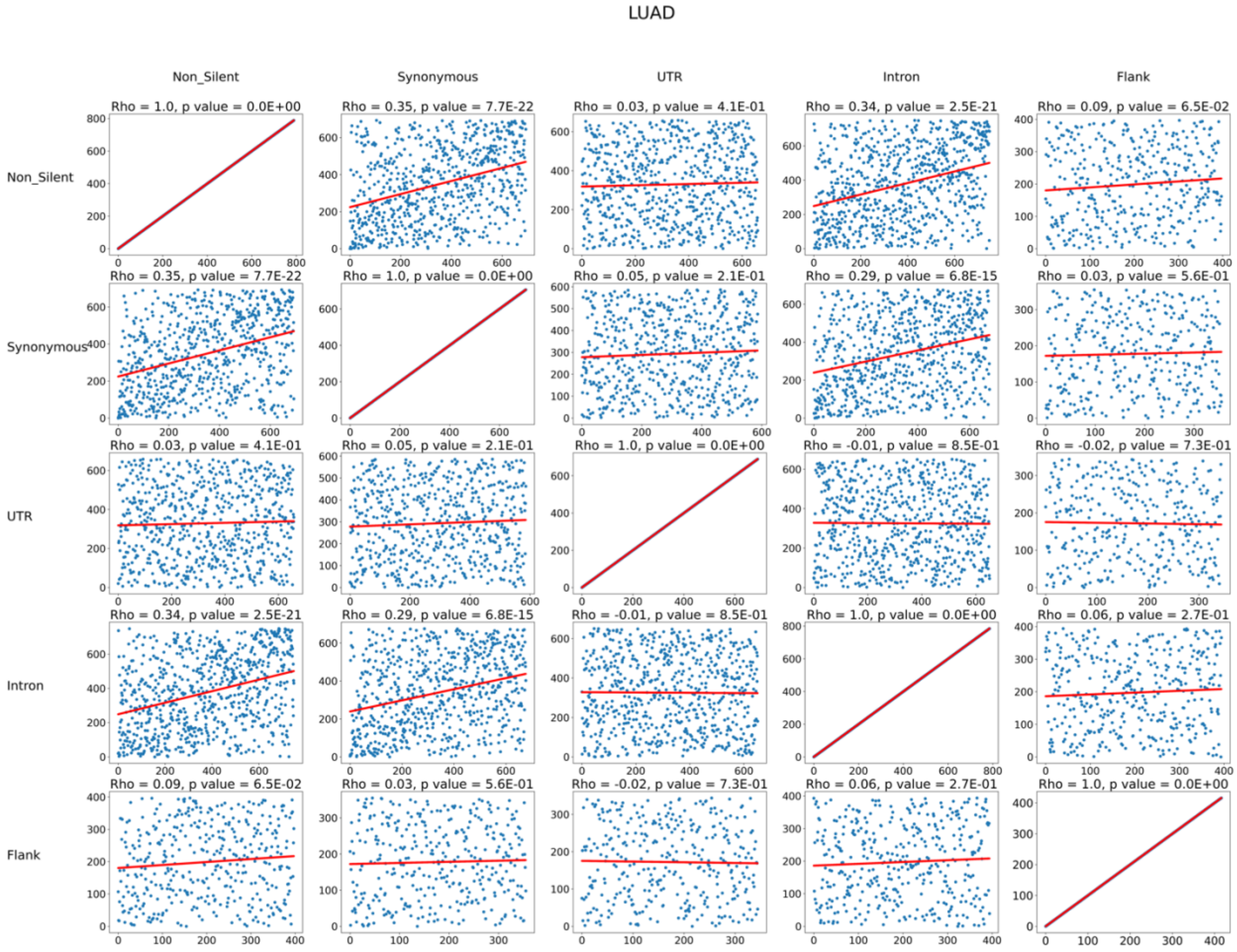
LUAD
Non_Silent
Synonymous
UTR
Intron
Flank
Non_Silent
Synonymous
UTR
Intron
Flank
Rho = 1.0, p value = 0.0E+00
Rho = 0.35, p value = 7.7E-22
Rho = 0.03, p value = 4.1E-01
Rho = 0.34, p value = 2.5E-21
Rho = 0.09, p value = 6.5E-02
Rho = 0.35, p value = 7.7E-22
Rho = 1.0, p value = 0.0E+00
Rho = 0.05, p value = 2.1E-01
Rho = 0.29, p value = 6.8E-15
Rho = 0.03, p value = 5.6E-01
Rho = 0.03, p value = 4.1E-01
Rho = 0.05, p value = 2.1E-01
Rho = 1.0, p value = 0.0E+00
Rho = -0.01, p value = 8.5E-01
Rho = -0.02, p value = 7.3E-01
Rho = 0.34, p value = 2.5E-21
Rho = 0.29, p value = 6.8E-15
Rho = -0.01, p value = 8.5E-01
Rho = 1.0, p value = 0.0E+00
Rho = 0.06, p value = 2.7E-01
Rho = 0.09, p value = 6.5E-02
Rho = 0.03, p value = 5.6E-01
Rho = -0.02, p value = 7.3E-01
Rho = 0.06, p value = 2.7E-01
Rho = 1.0, p value = 0.0E+00

l.

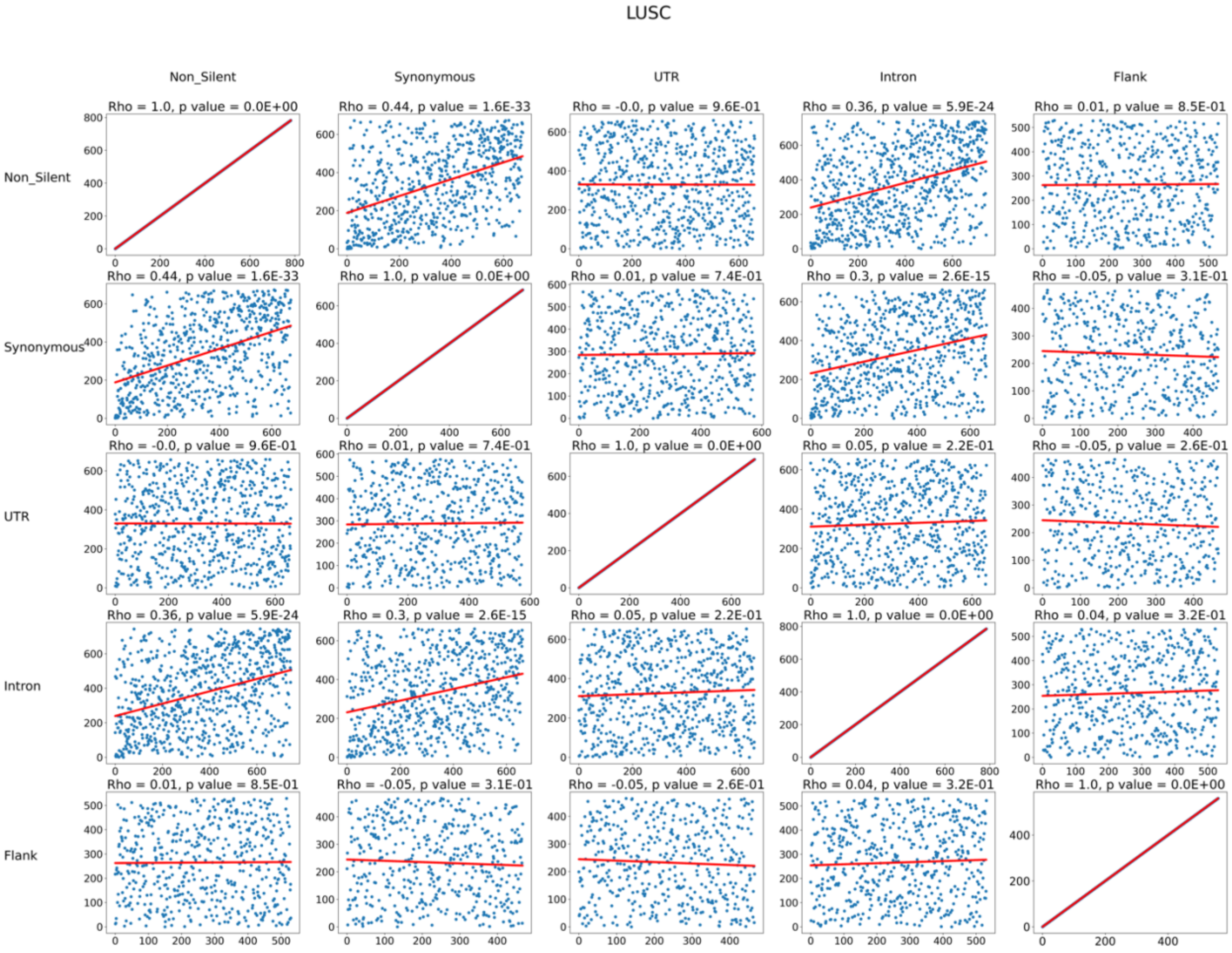
LUSC
Non_Silent
Synonymous
UTR
Intron
Flank
Rho = 1.0, p value = 0.0E+00
Rho = 0.44, p value = 1.6E-33
Rho = -0.0, p value = 9.6E-01
Rho = 0.36, p value = 5.9E-24
Rho = 0.01, p value = 8.5E-01
Rho = 0.44, p value = 1.6E-33
Rho = 1.0, p value = 0.0E+00
Rho = 0.01, p value = 7.4E-01
Rho = 0.3, p value = 2.6E-15
Rho = -0.05, p value = 3.1E-01
Rho = -0.0, p value = 9.6E-01
Rho = 0.01, p value = 7.4E-01
Rho = 1.0, p value = 0.0E+00
Rho = 0.05, p value = 2.2E-01
Rho = -0.05, p value = 2.6E-01
Rho = 0.36, p value = 5.9E-24
Rho = 0.3, p value = 2.6E-15
Rho = 0.05, p value = 2.2E-01
Rho = 1.0, p value = 0.0E+00
Rho = 0.04, p value = 3.2E-01
Rho = 0.01, p value = 8.5E-01
Rho = -0.05, p value = 3.1E-01
Rho = -0.05, p value = 2.6E-01
Rho = 0.04, p value = 3.2E-01
Rho = 1.0, p value = 0.0E+00

m.

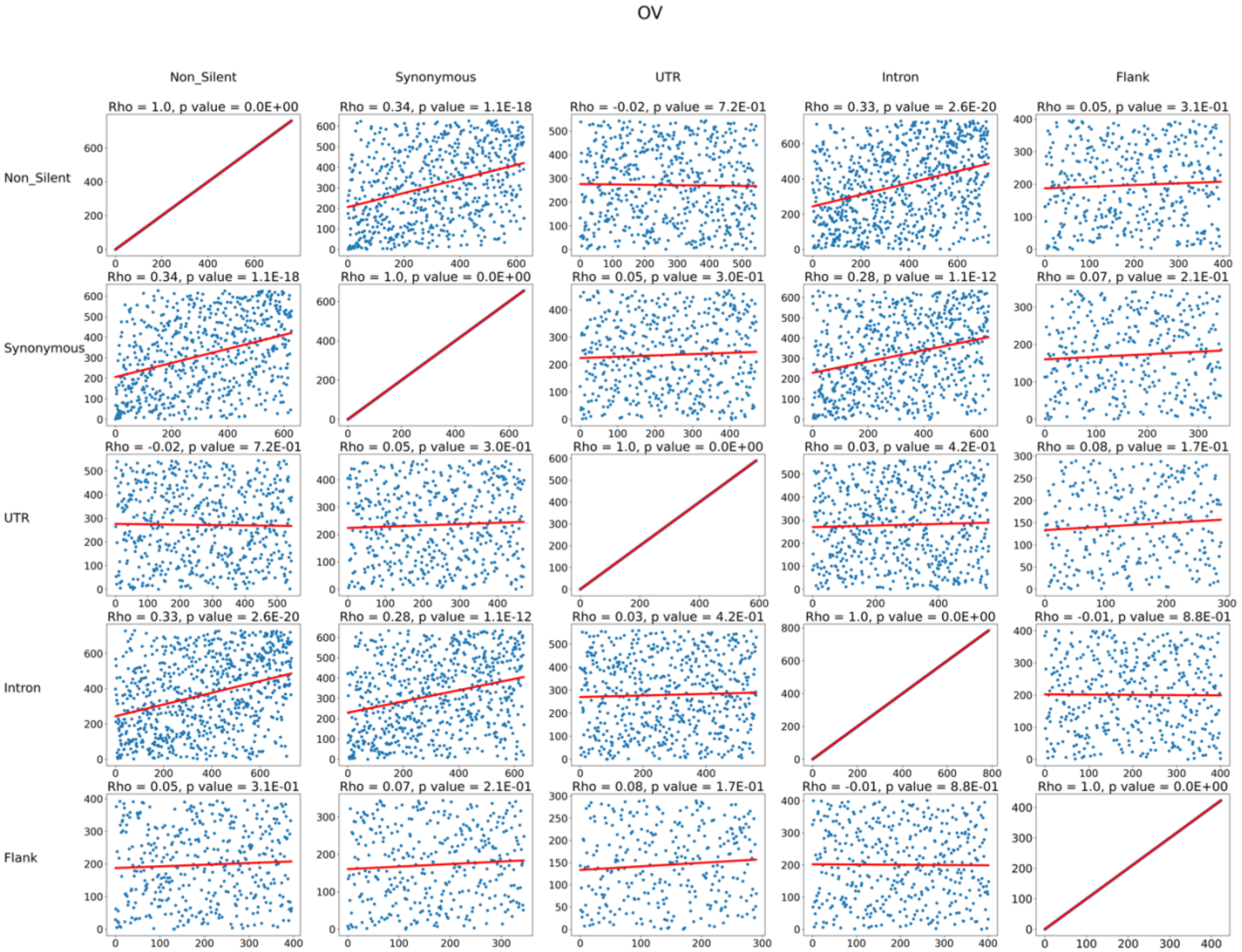

OV
Non_Silent
Synonymous
UTR
Intron
Flank
Rho = 1.0, p value = 0.0E+00
Rho = 0.34, p value = 1.1E-18
Rho = -0.02, p value = 7.2E-01
Rho = 0.33, p value = 2.6E-20
Rho = 0.05, p value = 3.1E-01
Rho = 0.34, p value = 1.1E-18
Rho = 1.0, p value = 0.0E+00
Rho = 0.05, p value = 3.0E-01
Rho = 0.28, p value = 1.1E-12
Rho = 0.07, p value = 2.1E-01
Rho = -0.02, p value = 7.2E-01
Rho = 0.05, p value = 3.0E-01
Rho = 1.0, p value = 0.0E+00
Rho = 0.03, p value = 4.2E-01
Rho = 0.08, p value = 1.7E-01
Rho = 0.33, p value = 2.6E-20
Rho = 0.28, p value = 1.1E-12
Rho = 0.03, p value = 4.2E-01
Rho = 1.0, p value = 0.0E+00
Rho = -0.01, p value = 8.8E-01
Rho = 0.05, p value = 3.1E-01
Rho = 0.07, p value = 2.1E-01
Rho = 0.08, p value = 1.7E-01
Rho = -0.01, p value = 8.8E-01
Rho = 1.0, p value = 0.0E+00

n.

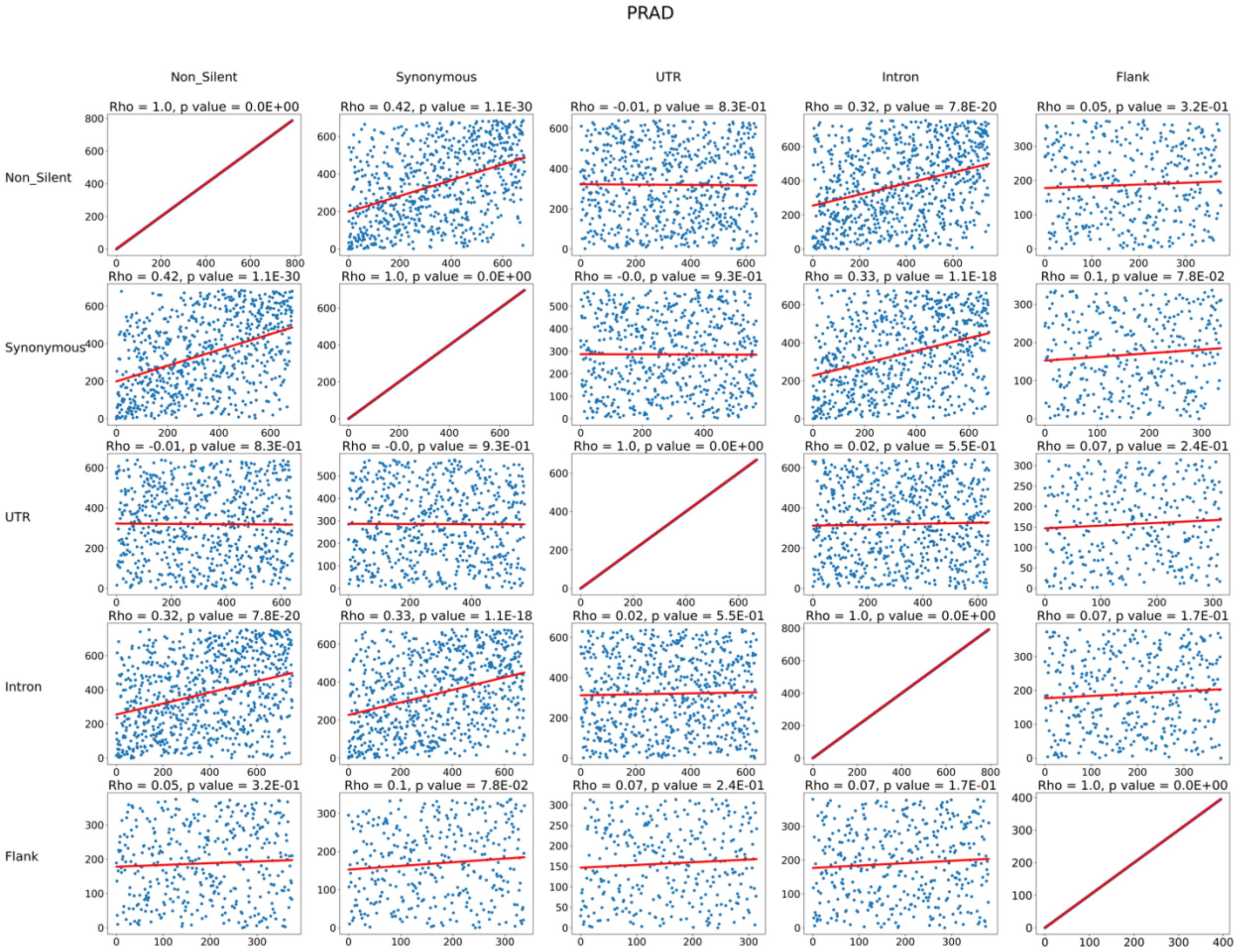
PRAD
Non_Silent
Synonymous
UTR
Intron
Flank
Rho = 1.0, p value = 0.0E+00
Rho = 0.42, p value = 1.1E-30
Rho = -0.01, p value = 8.3E-01
Rho = 0.32, p value = 7.8E-20
Rho = 0.05, p value = 3.2E-01
Rho = 0.42, p value = 1.1E-30
Rho = 1.0, p value = 0.0E+00
Rho = -0.0, p value = 9.3E-01
Rho = 0.33, p value = 1.1E-18
Rho = 0.1, p value = 7.8E-02
Rho = -0.01, p value = 8.3E-01
Rho = -0.0, p value = 9.3E-01
Rho = 1.0, p value = 0.0E+00
Rho = 0.02, p value = 5.5E-01
Rho = 0.07, p value = 2.4E-01
Rho = 0.32, p value = 7.8E-20
Rho = 0.33, p value = 1.1E-18
Rho = 0.02, p value = 5.5E-01
Rho = 1.0, p value = 0.0E+00
Rho = 0.07, p value = 1.7E-01
Rho = 0.05, p value = 3.2E-01
Rho = 0.1, p value = 7.8E-02
Rho = 0.07, p value = 2.4E-01
Rho = 0.07, p value = 1.7E-01
Rho = 1.0, p value = 0.0E+00

o.

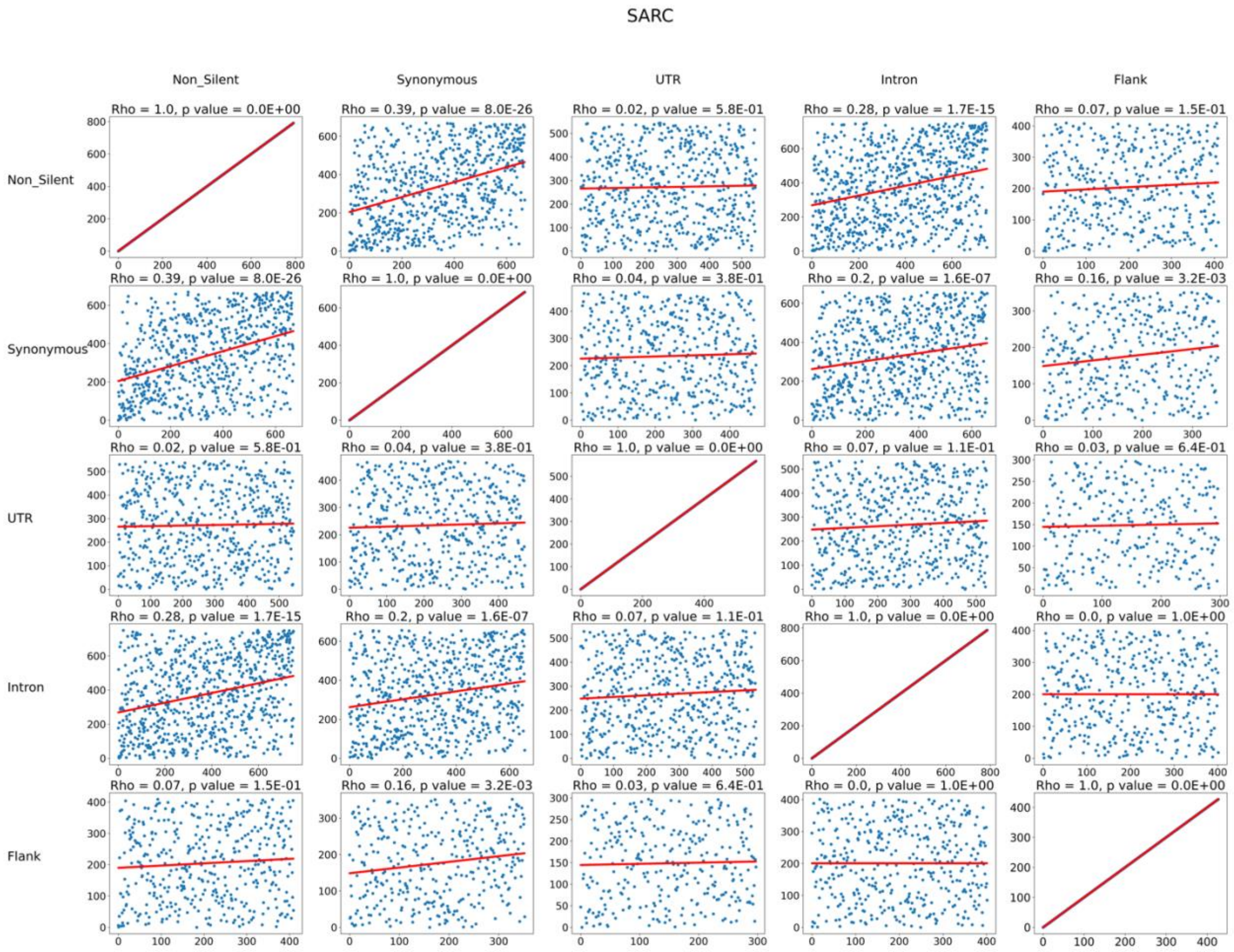
SARC
Non_Silent
Synonymous
UTR
Intron
Flank
Rho = 1.0, p value = 0.0E+00
Rho = 0.39, p value = 8.0E-26
Rho = 0.02, p value = 5.8E-01
Rho = 0.28, p value = 1.7E-15
Rho = 0.07, p value = 1.5E-01
Rho = 0.39, p value = 8.0E-26
Rho = 1.0, p value = 0.0E+00
Rho = 0.04, p value = 3.8E-01
Rho = 0.2, p value = 1.6E-07
Rho = 0.16, p value = 3.2E-03
Rho = 0.02, p value = 5.8E-01
Rho = 0.04, p value = 3.8E-01
Rho = 1.0, p value = 0.0E+00
Rho = 0.07, p value = 1.1E-01
Rho = 0.03, p value = 6.4E-01
Rho = 0.28, p value = 1.7E-15
Rho = 0.2, p value = 1.6E-07
Rho = 0.07, p value = 1.1E-01
Rho = 1.0, p value = 0.0E+00
Rho = 0.0, p value = 1.0E+00
Rho = 0.07, p value = 1.5E-01
Rho = 0.16, p value = 3.2E-03
Rho = 0.03, p value = 6.4E-01
Rho = 0.0, p value = 1.0E+00
Rho = 1.0, p value = 0.0E+00

p.

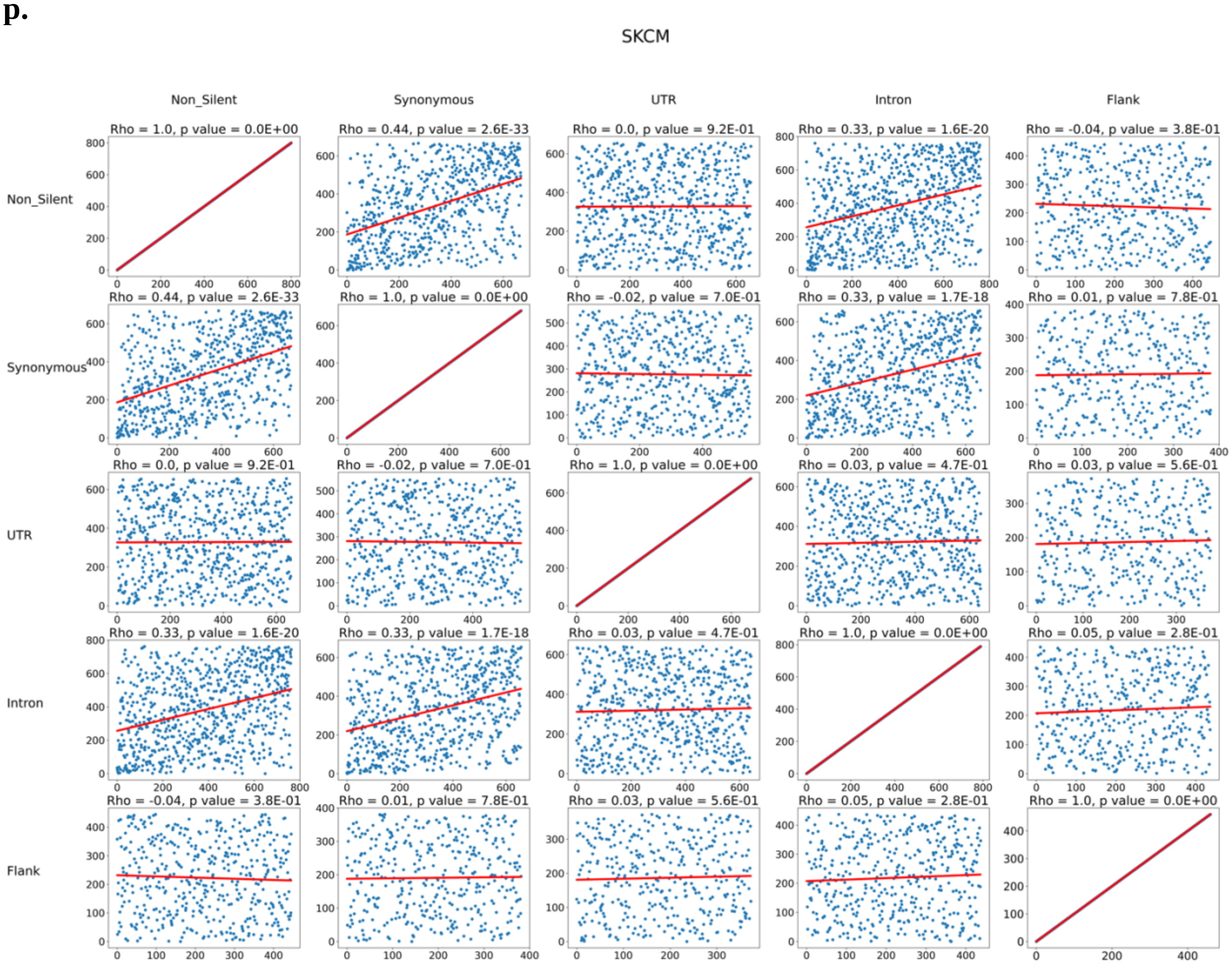
SKCM
Non_Silent
Synonymous
UTR
Intron
Flank
Rho = 1.0, p value = 0.0E+00
Rho = 0.44, p value = 2.6E-33
Rho = 0.0, p value = 9.2E-01
Rho = 0.33, p value = 1.6E-20
Rho = -0.04, p value = 3.8E-01
Rho = 0.44, p value = 2.6E-33
Rho = 1.0, p value = 0.0E+00
Rho = -0.02, p value = 7.0E-01
Rho = 0.33, p value = 1.7E-18
Rho = 0.01, p value = 7.8E-01
Rho = 0.0, p value = 9.2E-01
Rho = -0.02, p value = 7.0E-01
Rho = 1.0, p value = 0.0E+00
Rho = 0.03, p value = 4.7E-01
Rho = 0.03, p value = 5.6E-01
Rho = 0.33, p value = 1.6E-20
Rho = 0.33, p value = 1.7E-18
Rho = 0.03, p value = 4.7E-01
Rho = 1.0, p value = 0.0E+00
Rho = 0.05, p value = 2.8E-01
Rho = -0.04, p value = 3.8E-01
Rho = 0.01, p value = 7.8E-01
Rho = 0.03, p value = 5.6E-01
Rho = 0.05, p value = 2.8E-01
Rho = 1.0, p value = 0.0E+00

q.

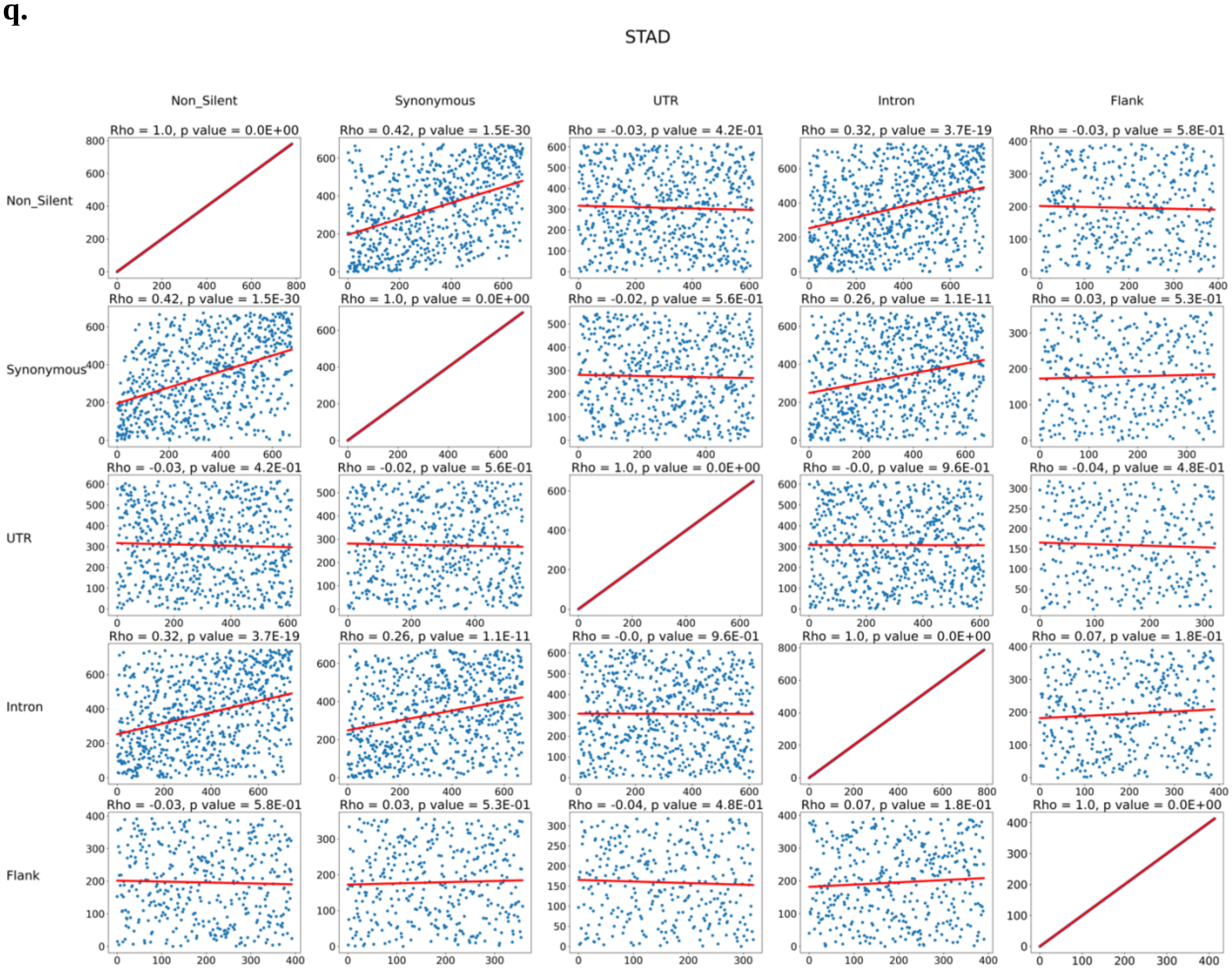
STAD
Non_Silent
Synonymous
UTR
Intron
Flank
Rho = 1.0, p value = 0.0E+00
Rho = 0.42, p value = 1.5E-30
Rho = -0.03, p value = 4.2E-01
Rho = 0.32, p value = 3.7E-19
Rho = -0.03, p value = 5.8E-01
Rho = 0.42, p value = 1.5E-30
Rho = 1.0, p value = 0.0E+00
Rho = -0.02, p value = 5.6E-01
Rho = 0.26, p value = 1.1E-11
Rho = 0.03, p value = 5.3E-01
Rho = -0.03, p value = 4.2E-01
Rho = -0.02, p value = 5.6E-01
Rho = 1.0, p value = 0.0E+00
Rho = -0.0, p value = 9.6E-01
Rho = -0.04, p value = 4.8E-01
Rho = 0.32, p value = 3.7E-19
Rho = 0.26, p value = 1.1E-11
Rho = -0.0, p value = 9.6E-01
Rho = 1.0, p value = 0.0E+00
Rho = 0.07, p value = 1.8E-01
Rho = -0.03, p value = 5.8E-01
Rho = 0.03, p value = 5.3E-01
Rho = -0.04, p value = 4.8E-01
Rho = 0.07, p value = 1.8E-01
Rho = 1.0, p value = 0.0E+00

r.

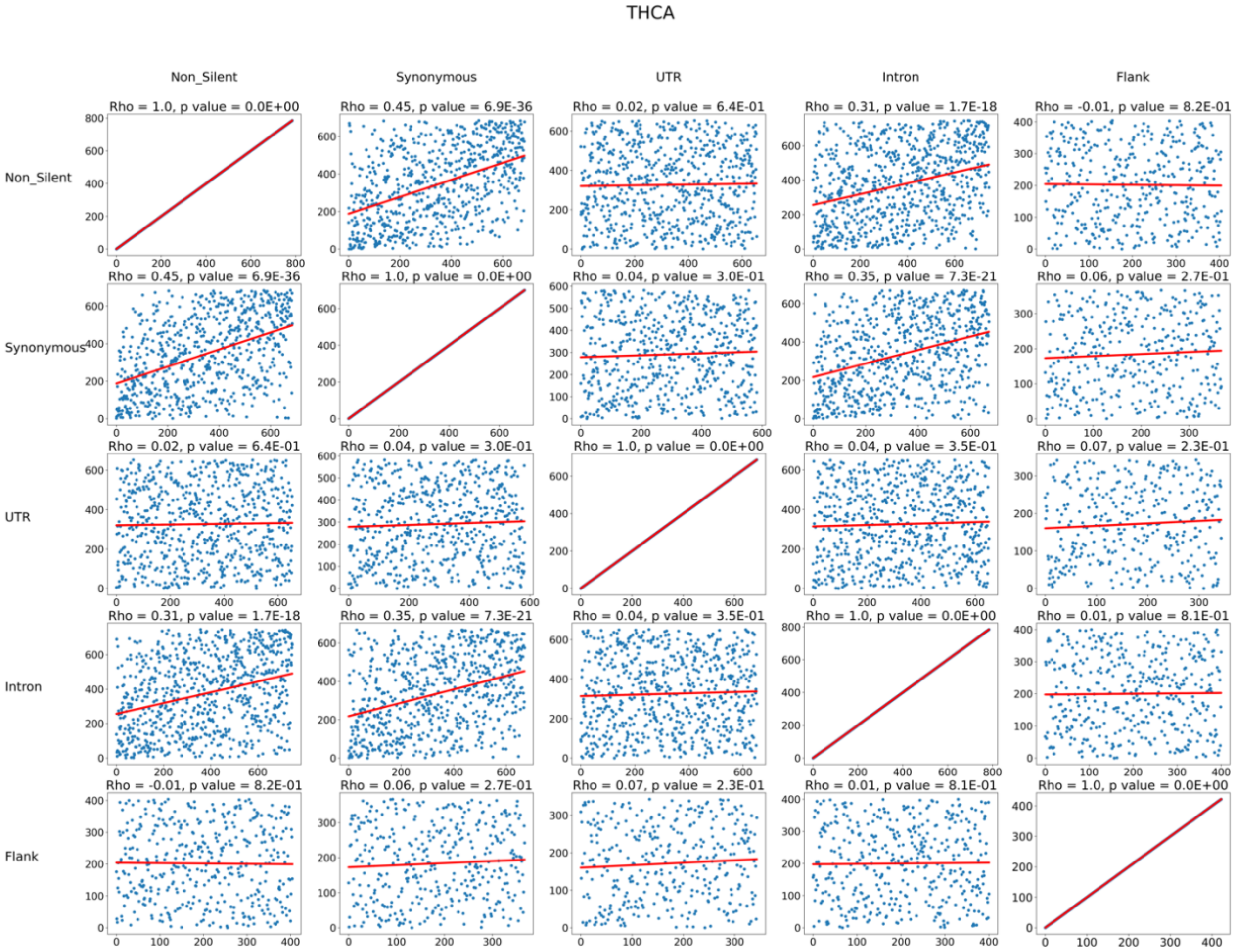
THCA
Non_Silent
Synonymous
UTR
Intron
Flank
Rho = 1.0, p value = 0.0E+00
Rho = 0.45, p value = 6.9E-36
Rho = 0.02, p value = 6.4E-01
Rho = 0.31, p value = 1.7E-18
Rho = -0.01, p value = 8.2E-01
Rho = 0.45, p value = 6.9E-36
Rho = 1.0, p value = 0.0E+00
Rho = 0.04, p value = 3.0E-01
Rho = 0.35, p value = 7.3E-21
Rho = 0.06, p value = 2.7E-01
Rho = 0.02, p value = 6.4E-01
Rho = 0.04, p value = 3.0E-01
Rho = 1.0, p value = 0.0E+00
Rho = 0.04, p value = 3.5E-01
Rho = 0.07, p value = 2.3E-01
Rho = 0.31, p value = 1.7E-18
Rho = 0.35, p value = 7.3E-21
Rho = 0.04, p value = 3.5E-01
Rho = 1.0, p value = 0.0E+00
Rho = 0.01, p value = 8.1E-01
Rho = -0.01, p value = 8.2E-01
Rho = 0.06, p value = 2.7E-01
Rho = 0.07, p value = 2.3E-01
Rho = 0.01, p value = 8.1E-01
Rho = 1.0, p value = 0.0E+00

s.

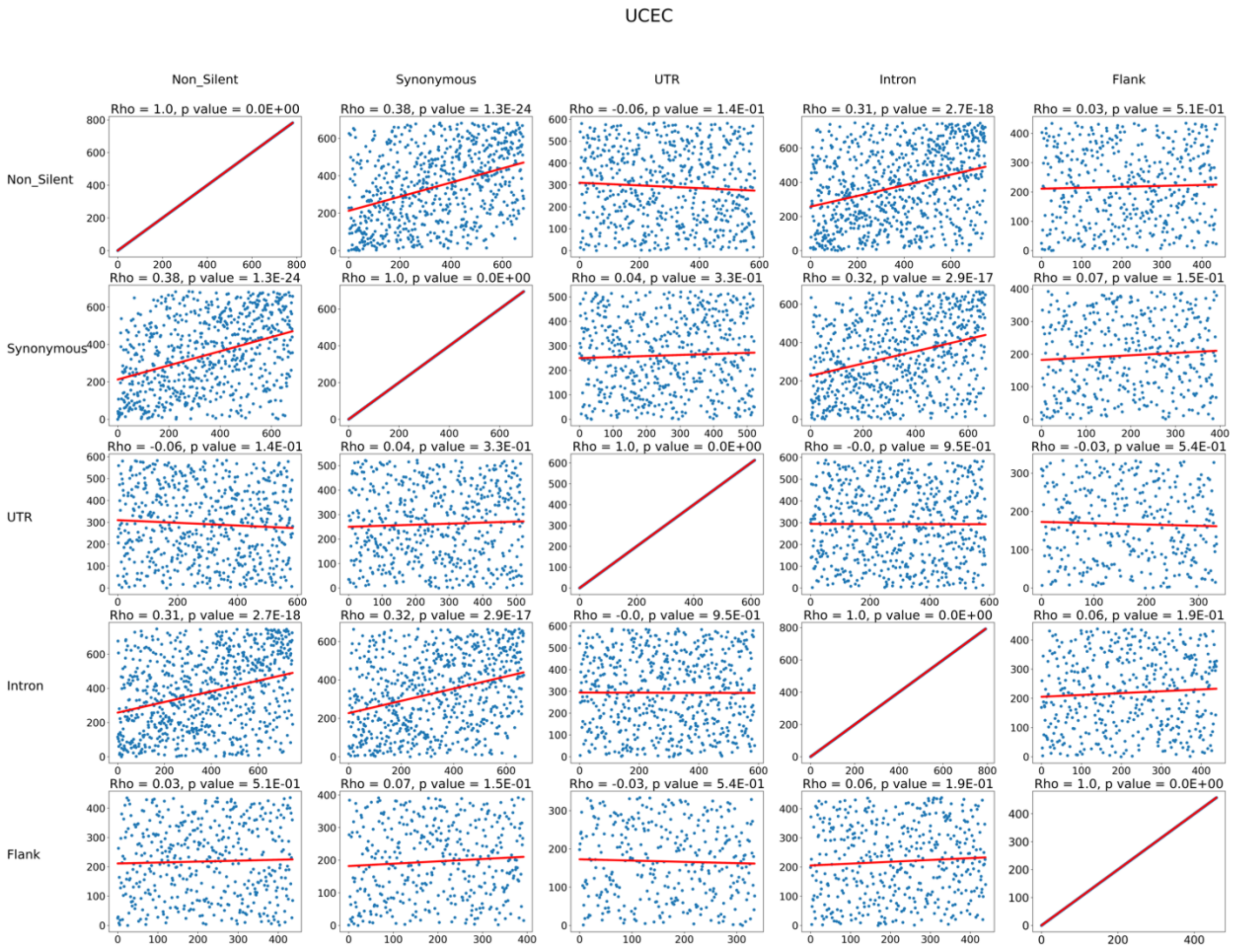

Supplementary Figure 6: Spearman correlations between gene rankings of pairs of models per cancer type. The all-features model was excluded from the analysis. Every subplot (a-s) represents a single cancer type. Within a subplot, every graph depicts the correlation between two models. A dot in the graph represents a gene. The x axis denotes the gene's rank given by the first model and the y axis denotes its rank given by the second model. The Spearman coefficient (Rho) and respective p value are noted above each graph.

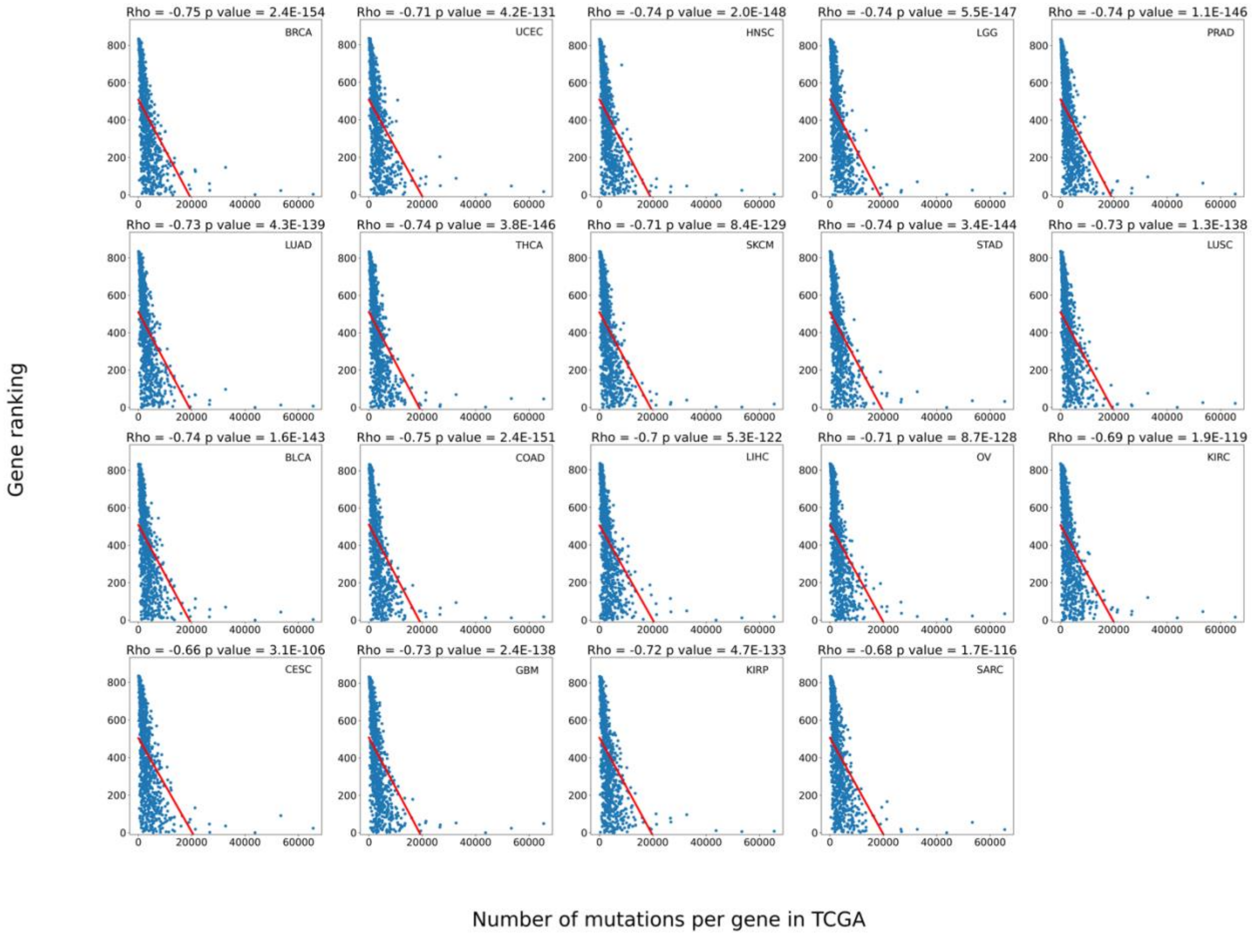

Supplementary Figure 7: Spearman correlation between the number of mutations documented per gene in the TCGA database and the gene's ranking obtained from the all-features models of the 19 cancer types. Each graph represents a single cancer type. A dot represents a single gene. The x axis denotes the number of mutations documented in TCGA for the gene and the y axis denotes the gene's ranking obtained from the all-features model. The Spearman coefficient (Rho) and respective p value are noted above each graph.

Supplementary Tables

Supplementary Table 1: F1 score improvement gained from adding lower resolution features

Cancer Type	High Resolution	High and Medium Resolutions	High, Medium and Low Resolutions	Total Improvement (%)
BRCA	0.72	0.76	0.83	15.27
UCEC	0.58	0.73	0.80	37.99
HNSC	0.31	0.34	0.43	38.59
LGG	0.80	0.85	0.85	5.79
PRAD	0.34	0.43	0.55	59.18
LUAD	0.39	0.52	0.63	61.38
THCA	0.63	0.64	0.69	10.22
SKCM	0.66	0.68	0.78	17.79
STAD	0.32	0.35	0.38	20.48
LUSC	0.28	0.29	0.44	60.61
BLCA	0.32	0.36	0.51	57.63
COAD	0.78	0.83	0.87	10.84
LIHC	0.64	0.69	0.74	15.92
OV	0.81	0.81	0.84	2.70
KIRC	0.57	0.68	0.79	38.37
CESC	0.37	0.41	0.46	25.46
GBM	0.46	0.45	0.50	10.09
KIRP	0.32	0.34	0.33	3.93
SARC	0.44	0.47	0.50	12.67

The F1 score improvement per cancer type that was achieved by adding medium resolution features and then low resolution features. The results were obtained using the all-features models.

Supplementary Table 2: Feature type distribution among the top 100 ranked features for each cancer type

Cancer\ Mutation Category	Non-Silent (%)	Intron (%)	UTR (%)	Flank (%)	Synonymous (%)
BLCA	64	33	0	2	1
BRCA	51	36	1	5	7
CESC	27	37	21	8	7
COAD	34	42	11	9	4
GBM	58	38	0	0	4
HNSC	53	39	1	2	5
KIRC	49	38	3	4	6
KIRP	53	37	5	4	1
LGG	48	47	0	0	5
LIHC	25	43	19	8	5

LUAD	48	40	0	2	10
LUSC	45	34	2	3	16
OV	44	45	6	2	3
PRAD	52	43	0	2	3
SARC	23	34	31	6	6
SKCM	48	36	0	2	14
STAD	53	37	2	0	8
THCA	59	29	0	1	11
UCEC	43	43	7	5	2

Feature rankings were obtained from the all-features models.

Supplementary Table 3: Feature type distribution among the top 10 ranked features for each cancer type

Cancer\ Mutation Category	Non-Silent (%)	Intron (%)	UTR (%)	Flank (%)	Synonymous (%)
BLCA	80	10	0	10	0
BRCA	60	30	0	10	0
CESC	20	10	50	10	10
COAD	40	10	0	10	0
GBM	70	30	0	0	0
HNSC	80	10	0	10	0
KIRC	60	40	0	0	0
KIRP	60	40	0	0	0
LGG	80	20	0	0	0
LIHC	30	40	20	0	10
LUAD	100	0	0	0	0
LUSC	90	10	0	0	0
OV	80	20	0	0	0
PRAD	80	20	0	0	0
SARC	30	10	60	0	0
SKCM	90	0	0	0	10
STAD	80	20	0	0	0
THCA	80	10	0	0	10
UCEC	80	10	0	0	10

Feature rankings were obtained from the all-features models.

Supplementary Table 4: Feature resolution distribution in the top 10 features of the models of the classification task

Cancer\ Mutation Category	All	Non-Silent	Intron	UTR	Flank	Synonymous
BLCA						
Low	7	6	7	8	4	6
Medium	1	2	3	2	6	3
High	2	2	0	0	0	1
BRCA						
Low	6	6	1	2	4	5
Medium	3	3	8	1	3	1
High	1	1	1	7	3	4
CESC						
Low	7	6	2	9	5	4
Medium	3	3	7	1	5	3
High	0	1	1	0	0	3
COAD						
Low	2	5	2	4	3	4
Medium	4	2	6	3	4	2
High	4	3	2	3	3	4
GBM						
Low	6	6	5	6	3	4
Medium	3	3	2	1	5	2
High	1	1	3	3	2	4
HNSC						
Low	7	5	5	7	4	5
Medium	2	3	3	3	5	3
High	1	2	2	0	1	2
KIRC						
Low	3	5	1	3	3	4
Medium	1	1	3	2	5	3
High	6	4	6	5	2	3
KIRP						
Low	6	5	6	4	3	8
Medium	3	4	3	2	6	2
High	1	1	1	4	1	0
LGG						
Low	7	6	8	10	4	7
Medium	2	3	2	0	6	2
High	1	1	0	0	0	1
LIHC						
Low	7	5	5	3	4	4
Medium	2	4	4	4	4	4
High	1	1	1	3	2	2
LUAD						
Low	7	7	8	7	3	6
Medium	2	2	2	3	5	1

High	1	1	0	0	2	3
LUSC						
Low	7	7	7	8	3	6
Medium	3	3	2	2	6	2
High	0	0	1	0	1	2
OV						
Low	3	4	1	4	3	3
Medium	2	2	3	5	3	4
High	5	4	6	1	4	3
PRAD						
Low	7	6	9	6	3	8
Medium	1	1	1	3	6	1
High	2	3	0	1	1	1
SARC						
Low	7	4	2	7	3	6
Medium	3	5	8	3	7	1
High	0	1	0	0	0	3
SKCM						
Low	7	7	9	8	2	10
Medium	2	2	1	1	7	0
High	1	1	0	1	1	0
STAD						
Low	3	1	4	4	3	6
Medium	5	1	5	6	5	2
High	2	4	1	0	2	2
THCA						
Low	5	5	7	9	4	6
Medium	3	2	2	1	6	0
High	2	3	1	0	0	4
UCEC						
Low	7	7	5	6	3	2
Medium	2	2	4	2	5	6
High	1	1	1	2	2	2

Supplementary Table 5: Mutations within genomic positions spanned by the silent features in the 10 top ranked features list of the all-features model that were found to have an impact on regulation

Chr	Gene	Cancer	S_Pos	E_Pos	V_Classification	Mut	V_Type	Ref_Al	Tum_Al	Prev	Splice	miRNA	mRNA_L	polyA	3D_Fold	Bind_Site
chr7	KMT2C	BLCA	152265211	152265211	Splice_Site	Intron	SNP	T	C	11.0294	Missed	None	None	None	None	None
chr7	KMT2C	BRCA	152265211	152265211	Splice_Site	Intron	SNP	T	C	11.437	Missed	None	None	None	None	None
chr8	PABPC1	CESC	100703049	100703050	3'UTR	UTR	INS	-	CCTCT	5.2805	None	0:canceled,1:discovered	None	None	None	None
chr3	SRGAP3	CESC	8985094	8985095	3'UTR	UTR	DEL	AT	-	16.5017	None	0:canceled,1:discovered	None	None	None	None
chr7	EGFR	GBM	55020559	55020560	Intron	Intron	INS	-	ACACACAC	0.6849	None	None	-0.7033	None	None	None
chr7	KMT2C	HNSC	152265211	152265211	Splice_Site	Intron	SNP	T	C	11.6601	Missed	None	None	None	None	None
chr19	MUC16	KIRP	8896093	8896093	Splice_Region	Intron	SNP	A	C	1.4134	New	None	None	None	None	None
chr17	TP53	LUSC	7673610	7673610	Splice_Site	Intron	SNP	T	C	0.6897	Missed	None	None	None	None	None
chr7	KMT2C	PRAD	152265211	152265211	Splice_Site	Intron	SNP	T	C	11.9675	Missed	None	None	None	None	None
chr3	SRGAP3	SARC	8985094	8985095	3'UTR	UTR	DEL	AT	-	23.1076	None	0:canceled,1:discovered	None	None	None	None
chr3	SRGAP3	SARC	8985094	8985097	3'UTR	UTR	DEL	ATAT	-	1.1952	None	0:canceled,1:discovered	None	None	None	None
chr8	PABPC1	SARC	100703049	100703050	3'UTR	UTR	INS	-	CCTCT	5.9761	None	0:canceled,1:discovered	None	None	None	None
chr2	LRP1B	THCA	140868264	140868264	Splice_Site	Intron	SNP	C	T	0.823	Missed	None	None	None	None	None
chr2	LRP1B	THCA	140541180	140541180	Intron	Intron	SNP	G	C	0.823	New	None	None	None	None	None
chr2	LRP1B	THCA	140541105	140541105	Splice_Region	Intron	SNP	T	C	0.6173	New	None	None	None	None	None

For each cancer type, we searched for mutations that are in the genomic positions spanned by the top 10 genomic elements (whether they are high, medium or low resolution features) that impact expression regulation. This table lists silent mutations that affected at least 0.5% of patients of a certain cancer type and were found to have an impact on regulation. Columns: Chr – the chromosome in which the mutation occurred. Gene – the gene in which the mutation occurred. Cancer – the patient cohort that was found affected by the mutation. S-Pos – start position of the mutation. E-Pos – end position of the mutation. V_Classification- variant classification (definition explained in the main text). V_Type – variant type (definition explained in the main text). Ref-Al – reference allele. The nucleotide context found in the discussed genomic position in the healthy tissue sample of the patient. Tum-Al- Tumor allele. The nucleotide context found in the discussed genomic position in the cancerous tissue of the patient. Prev – Prevalence. Percent of the patients in the cohort which are positive for the mutation. Splice - effect on splicing. “Missed” implies at least one missed site, “New” implies at least one newly discovered site. Threshold set used is (0.95.0.05). Model used is SpliceAI. miRNA - effect on miRNA target site. <number of canceled target sites>, <number of newly discovered sites>. Threshold used is 0.95. Model used is cnnMirTarget. mRNA_L - percentage of change in mRNA levels. “None” implies that no measurement exists since the mutation position is outside the model range (7000 [3500] US [DS] of TSS). Model used is Xpresso. PolyA - effect on Polyadenylation. Model used is SANPolyA. Threshold used is 0.9. 3D_Fold - effect on 3D DNA folding. Showing the p-value of the L2 difference if it is less than 0.05, otherwise “None”. We used the model only for 'large' INS and DEL mutations. Model used is Akita. Bind_Site - effect on the binding sites for the proteins TDP-43, hnRNPA1, and ELAVL1. Model used is DeepCLIP. Threshold used is 0.9.

Supplementary Table 6: Mutations within genomic positions spanned by the non-silent features in the 10 top ranked features list of the all-features model that were found to have an impact on regulation

Chr	Gene	Cancer	S_Pos	E_Pos	V_Classification	Mut	V_Type	Ref_Al	Tum_Al	Prev	Splice	miRNA	mRNA_L	polyA	3D_Fold	Bind_Site
chr12	KRAS	COAD	25227343	25227343	Missense_Mutation	Non_Synon	SNP	G	T	0.7463	Missed+New	None	None	None	None	None
chr2	IDH1	LGG	208248389	208248389	Missense_Mutation	Non_Synon	SNP	G	T	1.8145	New	None	None	None	None	None

For each cancer type, we searched for mutations that are in the genomic positions spanned by the top 10 genomic elements (whether they are high, medium or low resolution features) that impact expression regulation. This table lists non-silent mutations that affected at least 0.5% of patients of a certain cancer type and were found to have an impact on regulation. Columns are as defined for Supplementary Table 6.

Supplementary Table 7: Performance evaluations for the classification task

Cancer	Mutation Type	Accuracy	Precision	Recall	F1
BRCA	Non_Silent	0.937888	0.704544	0.849511	0.769981
	Intron	0.922749	0.654019	0.784039	0.712902
	UTR	0.828358	0.37717	0.611513	0.46635
	Flank	0.829786	0.412496	0.666776	0.509443
	Synonymous	0.859363	0.453658	0.727687	0.558721
	All	0.956335	0.795073	0.867101	0.829315
	Null	0.789641	0.130435	0.127036	0.128713
UCEC	Non_Silent	0.973904	0.802617	0.786335	0.794136
	Intron	0.9451	0.579184	0.531677	0.553973
	UTR	0.909762	0.367134	0.543125	0.437673
	Flank	0.861884	0.276652	0.605	0.379452
	Synonymous	0.86745	0.250468	0.534783	0.341057
	All	0.976016	0.848071	0.763975	0.80338
	Null	0.876892	0.04878	0.049689	0.049231
HNSC	Non_Silent	0.931275	0.439981	0.482895	0.460007
	Intron	0.917331	0.179814	0.1	0.127344
	UTR	0.691771	0.097277	0.489404	0.16226
	Flank	0.648888	0.101661	0.638806	0.175396
	Synonymous	0.853187	0.138949	0.275	0.184151
	All	0.938645	0.493147	0.375658	0.425865
	Null	0.886454	0.047619	0.046053	0.046823
LGG	Non_Silent	0.985976	0.917313	0.839597	0.876538
	Intron	0.905857	0.250749	0.287248	0.266849
	UTR	0.696168	0.113882	0.639716	0.193332
	Flank	0.714086	0.114297	0.668067	0.19516
	Synonymous	0.821036	0.17737	0.55302	0.268438
	All	0.983426	0.927123	0.78255	0.84832
	Null	0.883267	0.044304	0.04698	0.045603
PRAD	Non_Silent	0.937211	0.476957	0.65	0.549459
	Intron	0.911275	0.291977	0.35	0.317291
	UTR	0.712747	0.1182	0.626241	0.198848
	Flank	0.705669	0.103838	0.651327	0.179072
	Synonymous	0.823068	0.179502	0.560135	0.271737
	All	0.945339	0.535696	0.560135	0.546281
	Null	0.886056	0.027397	0.027027	0.027211
LUAD	Non_Silent	0.960797	0.694768	0.583562	0.633304
	Intron	0.925219	0.244942	0.139041	0.176895

	UTR	0.69161	0.094456	0.497241	0.158721
	Flank	0.66328	0.100228	0.63125	0.172958
	Synonymous	0.865378	0.140926	0.257534	0.181935
	All	0.963187	0.750786	0.55	0.633931
	Null	0.890837	0.067568	0.068493	0.068027
THCA	Non_Silent	0.964462	0.712246	0.654795	0.681673
	Intron	0.904821	0.204717	0.220548	0.211994
	UTR	0.704921	0.099928	0.509028	0.167024
	Flank	0.664893	0.103868	0.662205	0.17956
	Synonymous	0.833466	0.162624	0.44863	0.238598
	All	0.966773	0.754053	0.637671	0.690373
	Null	0.88247	0.048485	0.054795	0.051447
SKCM	Non_Silent	0.972749	0.760282	0.748571	0.754151
	Intron	0.961474	0.743645	0.473571	0.57747
	UTR	0.884147	0.239175	0.486331	0.320397
	Flank	0.862582	0.217401	0.535115	0.308788
	Synonymous	0.943108	0.489728	0.525	0.505752
	All	0.977092	0.83123	0.74	0.782825
	Null	0.889243	0.063291	0.071429	0.067114
STAD	Non_Silent	0.938566	0.418279	0.403788	0.408131
	Intron	0.947131	0.495697	0.234091	0.317424
	UTR	0.760266	0.114449	0.522901	0.187651
	Flank	0.741256	0.101751	0.555856	0.171875
	Synonymous	0.835299	0.119194	0.333333	0.175394
	All	0.950717	0.563853	0.289394	0.380185
	Null	0.899203	0.045113	0.045455	0.045283
LUSC	Non_Silent	0.940518	0.428643	0.41145	0.41909
	Intron	0.944861	0.424263	0.142748	0.212656
	UTR	0.696208	0.07937	0.452308	0.135017
	Flank	0.68321	0.080825	0.513043	0.139589
	Synonymous	0.904382	0.169199	0.212977	0.187997
	All	0.952112	0.565177	0.365649	0.443343
	Null	0.896414	0.048951	0.053435	0.051095
BLCA	Non_Silent	0.956932	0.573842	0.454098	0.504942
	Intron	0.93498	0.199184	0.111475	0.14176
	UTR	0.710286	0.089957	0.541322	0.154239
	Flank	0.678631	0.087791	0.626168	0.153955
	Synonymous	0.818088	0.10905	0.381148	0.169406
	All	0.96239	0.699819	0.4	0.507879
	Null	0.905578	0.03252	0.032787	0.032653
COAD	Non_Silent	0.984382	0.856971	0.812397	0.833233

	Intron	0.977131	0.811269	0.68843	0.743827
	UTR	0.951835	0.499927	0.701681	0.583307
	Flank	0.953685	0.550804	0.66	0.599686
	Synonymous	0.930398	0.350549	0.519008	0.41796
	All	0.988008	0.926984	0.816529	0.867704
	Null	0.906773	0.017094	0.016529	0.016807
LIHC	Non_Silent	0.969681	0.657982	0.667857	0.662515
	Intron	0.970717	0.713465	0.577679	0.637305
	UTR	0.943889	0.427466	0.705357	0.531883
	Flank	0.920497	0.337032	0.663063	0.446327
	Synonymous	0.934781	0.356302	0.571429	0.43881
	All	0.97757	0.77274	0.705357	0.737185
	Null	0.915139	0.053097	0.053571	0.053333
OV	Non_Silent	0.983665	0.822397	0.811607	0.816137
	Intron	0.979124	0.790378	0.727679	0.756882
	UTR	0.915651	0.282834	0.575676	0.378783
	Flank	0.874051	0.191711	0.53619	0.28142
	Synonymous	0.932311	0.352854	0.613393	0.447241
	All	0.985857	0.863324	0.8125	0.836788
	Null	0.917928	0.039216	0.035714	0.037383
KIRC	Non_Silent	0.982789	0.812534	0.692391	0.74684
	Intron	0.972191	0.766277	0.344565	0.474916
	UTR	0.904115	0.188997	0.479348	0.270844
	Flank	0.88273	0.139699	0.402299	0.207035
	Synonymous	0.94494	0.290481	0.351087	0.317242
	All	0.985936	0.8863	0.708696	0.786285
	Null	0.920319	0.042373	0.054348	0.047619
CESC	Non_Silent	0.958725	0.433989	0.438462	0.435522
	Intron	0.95753	0.379163	0.261538	0.308398
	UTR	0.930335	0.259546	0.48022	0.336199
	Flank	0.907327	0.200561	0.466292	0.280348
	Synonymous	0.920398	0.179818	0.334066	0.233495
	All	0.963984	0.509118	0.428571	0.462837
	Null	0.929084	0.042105	0.043956	0.043011
GBM	Non_Silent	0.974303	0.729894	0.425	0.535835
	Intron	0.968486	0.62707	0.25	0.354338
	UTR	0.861718	0.105846	0.389655	0.165602
	Flank	0.766245	0.064036	0.429487	0.111219
	Synonymous	0.945179	0.246653	0.277273	0.260476
	All	0.974422	0.786253	0.372727	0.504347
	Null	0.935857	0.060241	0.056818	0.05848

KIRP	Non_Silent	0.964024	0.455122	0.296471	0.358384
	Intron	0.967052	0.5459	0.192941	0.283395
	UTR	0.910891	0.162886	0.382353	0.228101
	Flank	0.884038	0.134347	0.412195	0.202509
	Silent	0.941594	0.168519	0.187059	0.176377
	All	0.968048	0.573311	0.236471	0.332414
	Dummy	0.930677	0.031579	0.035294	0.033333
SARC	Non_Silent	0.963984	0.408504	0.450667	0.427206
	Intron	0.967171	0.420645	0.258667	0.31931
	UTR	0.956232	0.351003	0.522667	0.419432
	Flank	0.939686	0.28468	0.556	0.375932
	Synonymous	0.930757	0.174332	0.352	0.232866
	All	0.972311	0.551	0.462667	0.500076
	Null	0.939841	0.012821	0.013333	0.013072

Accuracy, Precision, Recall and F1 scores of the six models (Non-Silent, Intron, UTR, Flank, Synonymous, All-Features) for classifying the 19 cancer types participating in the cancer type classification task, compared to a null model.

Supplementary Data

Supplementary Data 1 (an xlsx file): Feature importance rankings of the cancer type classification task. Every feature with importance higher than zero is listed. Each sub-table (a-s) holds the feature importance rank of the six models (non-silent, UTR, intron, synonymous, flank and all-features) for a single cancer type.

Supplementary Data 2 (an xlsx file): Gene Ontology and pathway enrichment. a Non-redundant gene ontology terms that were found significantly enriched (p-value < 0.001, FDR< 0.05) for the different combinations of mutation types and cancer types. **b** Gene ontology terms that were found significantly enriched by only a single model. The terms are not split according to the cancer types for which they were found significant. If a term was found significant for one or more cancer types by a single model it will appear the model's GO terms list. **c** Pathways found significantly enriched (FDR<0.05) by the REACTOME pathway analysis for the different combinations of mutation types and cancer types. **d** Pathways found significantly enriched by the REACTOME pathway analysis by only a single model. The terms are not split according to the cancer types for which they were found significant. If a pathway was found significant for one or more cancer types by a single model it will appear the model's pathway list.

Supplementary Data 3 (an xlsx file): Feature importance rankings of the survival estimation task. Every feature with importance higher than zero is listed. The table holds the feature importance rank of the six models (non-silent, UTR, intron, synonymous, flank and all-features).
