# Supplementary material for: Estimating the predictive power of silent mutations on cancer classification and prognosis
Source: NPJ Genom Med. 2021 Aug 12;6:67. doi: 10.1038/s41525-021-00229-1 (PMC8361094; doi:10.1038/s41525-021-00229-1)
Supplement: Supplementary file 5 — Reporting Summary [file 41525_2021_229_MOESM5_ESM.pdf]

nature research

Corresponding author(s): Tamir Tuller

Last updated by author(s): Apr 5, 2021

Reporting Summary

Nature Research wishes to improve the reproducibility of the work that we publish. This form provides structure for consistency and transparency in reporting. For further information on Nature Research policies, see our Editorial Policies and the Editorial Policy Checklist.

Statistics

For all statistical analyses, confirm that the following items are present in the figure legend, table legend, main text, or Methods section.

n/a	Confirmed	
☐	☒	The exact sample size (n) for each experimental group/condition, given as a discrete number and unit of measurement
☐	☒	A statement on whether measurements were taken from distinct samples or whether the same sample was measured repeatedly
☐	☒	The statistical test(s) used AND whether they are one- or two-sided<br>*Only common tests should be described solely by name; describe more complex techniques in the Methods section.*
☐	☒	A description of all covariates tested
☐	☒	A description of any assumptions or corrections, such as tests of normality and adjustment for multiple comparisons
☐	☒	A full description of the statistical parameters including central tendency (e.g. means) or other basic estimates (e.g. regression coefficient) AND variation (e.g. standard deviation) or associated estimates of uncertainty (e.g. confidence intervals)
☐	☒	For null hypothesis testing, the test statistic (e.g. F, t, r) with confidence intervals, effect sizes, degrees of freedom and P value noted<br>*Give P values as exact values whenever suitable.*
☐	☒	For Bayesian analysis, information on the choice of priors and Markov chain Monte Carlo settings
☐	☒	For hierarchical and complex designs, identification of the appropriate level for tests and full reporting of outcomes
☐	☒	Estimates of effect sizes (e.g. Cohen's d, Pearson's r), indicating how they were calculated

Our web collection on statistics for biologists contains articles on many of the points above.

Software and code

Policy information about availability of computer code

Data collection	The data was downloaded from the genomic data commons (https://portal.gdc.cancer.gov/).<br>No software was needed to do so.
Data analysis	The data analysis was performed with a custom code in Python. Many Python packages were used, including LightGBM and Pysurvival among other basic packages such as Pandas, Numpy, Matplotlib and Scikit-learn.

For manuscripts utilizing custom algorithms or software that are central to the research but not yet described in published literature, software must be made available to editors and reviewers. We strongly encourage code deposition in a community repository (e.g. GitHub). See the Nature Research guidelines for submitting code & software for further information.

Data

Policy information about availability of data

All manuscripts must include a data availability statement. This statement should provide the following information, where applicable:

- Accession codes, unique identifiers, or web links for publicly available datasets
- A list of figures that have associated raw data
- A description of any restrictions on data availability

The data used in this study was generated by The Cancer Genome Atlas (https://www.cancer.gov/tcga) and can be downloaded from the genomic data commons (https://portal.gdc.cancer.gov/).

Field-specific reporting

Please select the one below that is the best fit for your research. If you are not sure, read the appropriate sections before making your selection.

☒ Life sciences ☐ Behavioural & social sciences ☐ Ecological, evolutionary & environmental sciences

For a reference copy of the document with all sections, see nature.com/documents/nr-reporting-summary-flat.pdf

Life sciences study design

All studies must disclose on these points even when the disclosure is negative.

Sample size	The samples size of the patients' cohorts were chosen by the maximal number of patients in the TCGA database that had both genomic and clinical data. The results of our prediction models demonstrate that the sample size is enough to enable a fair training of the models.
Data exclusions	We excluded patients with missing genomic and clinical data becuase we need those features for our predictive models.
Replication	All findings can be reproduced by running the code on the data downloaded from TCGA. The only differences that could occur are due to randomizations within the models, but these are expected to have a small effect sense we performed multiple random splits to train and test sets.
Randomization	Patients are randomly split to train and test sets (70%/30%) in a stratified fashion. Meaning that we consider the label distribution. For example, if we have 90 BRCA negative patients and 10 BRCA positive examples, The model will randomly split the patients to a training set with 70 patients, about 10% of which will be BRCA positive, and a test set with 30 patients, about 10% of which will also be BRCA positive.
Blinding	The models were "blind" and did not have the labels of the test set patients when they had to predict them, so their decision was unbiased.

Reporting for specific materials, systems and methods

We require information from authors about some types of materials, experimental systems and methods used in many studies. Here, indicate whether each material, system or method listed is relevant to your study. If you are not sure if a list item applies to your research, read the appropriate section before selecting a response.

Materials & experimental systems

n/a	Involved in the study	
☒	☐	Antibodies
☒	☐	Eukaryotic cell lines
☒	☐	Palaeontology and archaeology
☒	☐	Animals and other organisms
☒	☐	Human research participants
☒	☐	Clinical data
☒	☐	Dual use research of concern

Methods

n/a	Involved in the study	
☒	☐	ChIP-seq
☒	☐	Flow cytometry
☒	☐	MRI-based neuroimaging
